# Relationship of Biochemical Response as Surrogate Endpoints with Long-Term Outcomes in Primary Biliary Cholangitis: A Global Systematic Review and Meta-Analysis

**DOI:** 10.36469/001c.170737

**Published:** 2026-10-30

**Authors:** Dilip Makhija, Marvin Rock, Chong H. Kim, Ryan Thaliffdeen, Pankaj Rai, Barinder Singh

**Affiliations:** 1 Global Value and Access Gilead Sciences, Inc., Foster City, California, USA; 2 HEOR Pharmacoevidence, Mohali, India

**Keywords:** composite response, bilirubin, biochemical response, primary biliary cholangitis, alkaline phosphatase, surrogate endpoints, liver transplantation

## Abstract

**Background:**

Primary biliary cholangitis (PBC) is a chronic autoimmune liver disease in which long-term clinical outcomes are difficult to assess within conventional trial durations. Alkaline phosphatase (ALP) and bilirubin are established biochemical endpoints in regulatory and clinical practice, and their relationship with long-term clinical outcomes has been demonstrated through a robust literature of analyses across different datasets. However, the magnitude and consistency of their association with long-term clinical outcomes have not been systematically quantified through meta-analysis.

**Objective:**

This systematic literature review and meta-analysis aimed to identify, evaluate, and summarize evidence on the correlation between surrogate biochemical endpoints and long-term clinical outcomes in patients with PBC.

**Methods:**

A comprehensive search of key biomedical databases and reporting was conducted, in accordance with Cochrane, NICE and PRISMA guidelines. Meta-analysis was performed using Stata 19.5 SE software, with the DerSimonian and Laird (DL) random-effects model employed in the base-case, inverse-variance fixed-effect and DL + Hartung-Knapp-Sidik-Jonkman random effects model were used in sensitivity analyses. Other sensitivity analyses were performed based on study/follow-up duration, study quality, publication type, and removal of heterogeneous studies.

**Results:**

Overall, 129 studies were included in the systematic literature review, among which 89 studies met criteria for meta-analysis. Failure to achieve ALP normalization was associated with significantly increased risks of composite clinical outcomes (HR; 95% CI: 1.46; 1.26-1.69), liver-related events (1.82; 1.41-2.34), and overall death and/or liver transplantation (1.52; 1.30-1.79). Similar findings were reported for other ALP response definitions. Nonresponse to composite criteria was also associated with significantly worse long-term outcomes. Sensitivity analyses yielded directionally consistent results.

**Discussion:**

To our knowledge, this research represents the first comprehensive meta-analytic evaluation integrating multiple surrogate biochemical endpoints with a broad spectrum of long-term clinical outcomes in PBC across a global study population. Associations were directionally consistent across ALP-, bilirubin-, and composite-based definitions and across all analyses.

**Conclusion:**

This meta-analysis provides robust evidence that ALP normalization, ALP-based response criteria, and composite biochemical response measures (comprising ALP and total bilirubin), were consistently associated with long-term clinical outcomes in PBC and may inform HTA, reimbursement, payer decision-making, and economic evaluation when mature long-term outcome data are unavailable.

## INTRODUCTION

Primary biliary cholangitis (PBC) is a rare, long-term autoimmune cholestatic liver disease in which clinically meaningful outcomes such as liver transplantation (LT), hepatic decompensation, and death may take many years to occur.^1,2^ The global prevalence and incidence of PBC are estimated at approximately 14.6 and 1.76 per 100 000 persons, respectively, underscoring both its rarity and its implications for long-term disease assessment.^3,4^ Ursodeoxycholic acid (UDCA) is the established first-line therapy for PBC and is recommended at a dose of 13 to 15 mg/kg/day.^5^ However, up to 40% of patients treated with UDCA have a persistently elevated alkaline phosphatase (ALP) level, defined as ≥1.67 × the upper limit of normal (ULN), abnormal serum total bilirubin, or both, which is associated with disease progression.^6,7^ Importantly, an even greater proportion of patients do not achieve complete ALP normalization (≤1 × ULN), which is increasingly recognized as an optimal treatment goal in PBC.^8^ Obeticholic acid (OCA) received US Food and Drug Administration (FDA) approval as a second-line therapy for patients with an inadequate response or intolerance to UDCA.^9^ However, its marketing authorization was revoked due to mechanism-specific safety concerns, including the risk of serious liver injury identified by the FDA,^10^ and the product was subsequently withdrawn from the US market in 2025.^11^ More recently, seladelpar and elafibranor received accelerated FDA approval in 2024^12,13^ for use in PBC patients with an inadequate response or intolerance to UDCA, based primarily on their ability to reduce ALP levels in the RESPONSE^6^ and ELATIVE^14^ trials, respectively. In addition to biochemical effects, seladelpar significantly improved pruritus in the pivotal study.^6^

A major challenge for healthcare providers, drug developers, and health technology assessment (HTA) bodies is the slow progression of PBC, which precludes the evaluation of long-term clinical outcomes over a 1-year period, as has been used in registrational studies for PBC.^15,16^ Long-term outcome studies are underway for seladelpar (NCT06051617)^17^ and elafibranor (NCT06016842),^18^ but require longer follow-up and pose inherent challenges.^15,19^ This challenge is particularly relevant in the era of widespread UDCA use, which has substantially improved patient outcomes and slowed disease progression,^20^ further extending the time required for clinically meaningful events such as LT and death to occur.^15,21^ In 2026, the European Association for the Study of the Liver (EASL) and American Association for the Study of Liver Diseases (AASLD) jointly convened a multi-stakeholder Delphi consensus panel that formally evaluated the role of liver biochemistry and non-invasive fibrosis measures as surrogate endpoints in PBC, concluding with high panel agreement that ALP and total bilirubin after 12 months of therapy are associated with clinical outcomes and recommended complete (normal ALP and bilirubin) or adequate biochemical response (ALP <1.5-1.67 × ULN and normal bilirubin) as ideal or acceptable primary endpoints in phase III trials.^15^ This emphasis on complete biochemical response reflects the growing recognition that ALP normalization is strongly and consistently associated with favorable long-term clinical outcomes in PBC. As a marker closely linked to the underlying cholestatic disease process, sustained reductions in ALP correlate with a lower risk of hepatic decompensation, LT, and liver-related mortality. Moreover, evidence suggests that more stringent biochemical responses, particularly complete ALP normalization, provide greater prognostic discrimination than partial reductions in ALP. Collectively, these findings support ALP normalization as a clinically meaningful measure of treatment response and a potentially informative early indicator of subsequent disease outcomes, especially in clinical trials where hard clinical endpoints require prolonged follow-up.^15^

The EUnetHTA guidelines acknowledge the acceptability of surrogate endpoints where there is biological plausibility for the endpoint, empirical evidence of association with final outcomes, and consideration of the wider risk-benefit profile and decision uncertainty.^22^ Importantly, several HTA bodies, including the National Institute for Health and Care Excellence (NICE UK), Federal Joint Committee (G-BA Germany), and Canada’s Drug Agency (CDA-AMC) have developed more prescriptive guidance outlining validation requirements for surrogate endpoints, typically emphasizing consistency of association with clinical outcomes supported by epidemiological data and, where possible, meta-analyses of randomized controlled trials (RCT).^22^ Surrogate biochemical markers, including ALP response, composite response, and bilirubin, have been accepted in prior NICE appraisals of PBC therapies such as obeticholic acid,^23^ elafibranor,^24^ and seladelpar.^25^

Consequently, biochemical endpoints such as ALP and bilirubin have become established surrogate markers for assessing treatment response and are already accepted by regulatory authorities, such as the FDA and European Medicines Agency (EMA)^26,27^ for accelerated approval and incorporated into clinical practice guidelines. Although these biomarkers are widely used in clinical trials, regulatory decision-making, and clinical care, the overall strength and consistency of their association with long-term clinical outcomes have not been comprehensively quantified across the available evidence base. Therefore, the objective of this systematic literature review (SLR) and meta-analysis is to identify, evaluate, and summarize evidence on the correlation between surrogate biochemical endpoints and long-term clinical outcomes in patients with PBC.

## METHODS

The SLR was conducted according to a prespecified internal protocol and followed NICE,^28^ Cochrane Handbook,^29^ and the Preferred Reporting Items for Systematic Reviews and Meta-Analysis (PRISMA)^30^ guidelines for systematic review conduct and reporting.

### Search Strategy and Selection Criteria

Key biomedical databases, including Embase, PubMed, and Cochrane, were extensively searched from inception through 3 June 2026, followed by the bibliography of relevant reviews. Further, conference proceedings, including the Professional Society for Health Economics and Outcomes Research, AASLD, EASL, Digestive Disease Week, and the International Liver Congress were searched for the last three years (2023 to 3 June 2026) to retrieve the latest studies that have not yet been published in journals as full-text articles. A detailed search strategy has been provided in **Supplementary Table S1**.

Study selection was conducted in two stages based on predefined eligibility criteria (**Supplementary Table S2**): (1) title and abstract screening and (2) full-text review of potentially relevant studies. Two reviewers independently performed both stages, with any conflicts resolved by a third reviewer. Where multiple publications were identified from the same cohort, registry, database, or clinical-study population, records were linked and assessed together.

Similarly, for data extraction, two investigators independently extracted the data for the final list of selected eligible studies, with conflict resolution and quality-checking conducted by a third independent reviewer. The most comprehensive, recent, and/or longest follow-up publication was preferentially included in the quantitative synthesis to minimize double-counting of participants.

### Assessment of Methodological Quality

Quality assessment for observational studies and RCTs was conducted using the Newcastle-Ottawa Scale (NOS)^31^ and Cochrane Collaboration Risk of Bias Tool (RoB 2.0),^32^ respectively, to characterize the evidence base, and for additional sensitivity analyses.

### Statistical Analysis

Meta-analysis was conducted using Stata 19.5 (StataCorp). Statistical heterogeneity was evaluated using Cochran’s Q, tau-squared (τ^2^), and I-squared (I^2^) statistics, where I^2^ values <25%, 25%-75%, and >75% indicated low, moderate, and high statistical heterogeneity, respectively.^33^

When a single study reported multiple eligible effect estimates for the same outcome, these estimates were combined into a single study-level estimate prior to meta-analysis, so that each study contributed only once to a given pooled analysis.^34^ Most studies reported effect estimates for non-responders vs responders and were pooled directly; estimates reported in the reverse direction were mathematically transformed accordingly. This harmonization allowed pooled estimates to be interpreted consistently and avoided potential misclassification of effect direction.

For studies reporting time-to-event outcomes, hazard ratios (HRs) were derived and analyzed on the log scale. Pooled estimates were derived using the DerSimonian and Laird (DL) 2-step estimator random-effects model (REM) as the base-case approach. Model-based sensitivity analyses used the inverse-variance (IV) fixed-effect model (FEM) and DL + Hartung-Knapp-Sidik-Jonkman (HKSJ) random-effects approaches.^35,36^

For categorical data, odds ratios (ORs) with corresponding 95% confidence intervals (95% CI) were computed using available event counts and sample sizes. A DL REM was applied in the base-case analysis to account for between-study heterogeneity when variability across studies was anticipated or observed.^33,37^ The Mantel-Haenszel (MH) FEM and HKSJ model were employed for the sensitivity analysis.

Sensitivity analyses were conducted to evaluate the robustness of the findings by excluding low-quality studies, conference abstracts, and studies with unreported follow-up, and by restricting analyses to studies with reported durations or follow-up periods >12 months. Additional analyses examined adjusted vs unadjusted effect estimates, and influence diagnostics were performed to assess the impact of studies contributing substantially to heterogeneity.

Estimates (HR/OR) were synthesized on the log scale with back-transformation for reporting. For OR analyses, studies containing zero-event cells were retained in the meta-analysis where appropriate. A continuity correction of 0.5 was automatically applied by Stata to zero cells to enable estimation of odds ratios and variances. This approach ensured that eligible studies were not unnecessarily excluded from the quantitative synthesis while maintaining consistency across analyses.

Publication bias was assessed using funnel plots, where asymmetry may suggest potential bias, such as selective reporting. Egger’s test was used to determine the statistical significance of asymmetry.^38^

## RESULTS

### Search Results

A PRISMA diagram for the screening process is presented in **Supplementary Figure S1**. A total of 3770 publications were identified from the searches conducted in key biomedical databases. After removing 133 duplicate publications, a total of 3637 publications were screened based on title and abstract, of which 3143 publications did not meet the inclusion criteria. The full-text screening for 494 publications was completed, of which 339 publications did not meet the review criteria and were excluded. Additionally, 28 publications were identified from conference and bibliography searching. Finally, a total of 129 studies from 183 publications were included in the SLR (**Supplementary Table S3**). Of these, 89 studies from 139 publications were eligible for meta-analysis, while 44 publications were ineligible for quantitative synthesis (**Supplementary Table S16**).

Although several types of surrogate markers and clinical outcomes were reported across studies, the current analysis focuses on ALP normalization, ALP response, composite response, other surrogate markers, and their association with composite clinical outcomes, any liver-related events, and overall death and/or LT.

All-cause mortality was defined as death from any cause, liver-

related mortality as death due to liver disease or its complications, and death/LT as the occurrence of death or LT. As transplant-free survival represents the inverse of death/LT, it was classified within the death/LT category.

Any liver-related event was harmonized by including ≥1 liver-

related clinical outcomes, comprising hospitalization, bile duct loss, hepatic decompensation, disease progression, esophageal or gastric variceal bleeding, cirrhosis, hepatic encephalopathy, hepatocellular carcinoma (HCC), spontaneous bacterial peritonitis, LT, death or ascites. Composite clinical outcomes were defined as death due to any cause and/or LT with ≥1 major liver-related event.

### Study and Population Characteristics

**Supplementary Table S3** summarizes the characteristics of the 129 studies included in this SLR. Of these, 75 were peer-reviewed journal articles, while 54 were conference abstracts. The sample sizes ranged from 35^39^ to 22 487.^40^ Most of the observational studies (n = 93) were rated as high quality (NOS score, 6-9), followed by 32 studies with fair quality (NOS score, 3-5), while the single remaining study was rated as low quality (NOS score, 0-2).^41^ The domain-level assessment showed that limitations were not evenly distributed across NOS items. Comparability for additional factors like age, gender, and comorbidities was frequently not met, and several conference abstracts had insufficient information to assess outcome ascertainment or adequacy and duration of follow-up (**Supplementary Table S5; Supplementary Figure S2**). All 3 RCTs were judged to have some concerns regarding risk of bias, arising from the randomization process and deviations from intended interventions (**Supplementary Table S6; Supplementary Figure S3**).

Among the 129 included studies, 111 studies provided both age-specific and gender-specific information. The mean age across studies ranged from 46.1 years^42^ to 68.4 years,^43^ whereas all studies except two^44,45^ reported a higher proportion of female patients (>50%), consistent with the recognized female predominance of PBC (**Supplementary Table S4**).

### ALP Response

Overall, 62 studies reported an association between ALP response and long-term clinical outcomes and were considered for meta-analysis. The most commonly used ALP response criteria were Toronto I (ALP ≤1.67 × ULN; n = 27), Barcelona (ALP reduction ≥40% or ALP ≤1 × ULN; n = 19), other ALP cut-offs (n = 18), ALP normalization (n = 16), followed by Rochester II (ALP ≥2 × ULN; n = 7) and Toronto II (ALP ≤1.76 × ULN; n = 4).

### ALP Normalization

Overall, 16 studies were eligible for meta-analysis, which reported an association of ALP normalization and long-term clinical outcomes. Among different long-term clinical outcomes, 14 studies reported an association of ALP normalization with composite clinical outcomes. Further, 12 studies reported an association of ALP normalization with overall death and/or LT, and 8 studies reported an association of ALP normalization with any liver-related events. The pooled estimates (non-responders vs responders) from these analyses showed that responders had a significantly lower risk of composite clinical outcomes (HR, 1.46; 95% CI, 1.26-1.69), any liver-related events (HR, 1.82; 95% CI, 1.41-2.34), and overall death and/or LT (HR, 1.52; 95% CI, 1.30-1.79) compared with non-responders (**Figure 1A**). Similar findings were reported for the studies reporting categorical data (**Figure 1B**).

**Figure 1.**
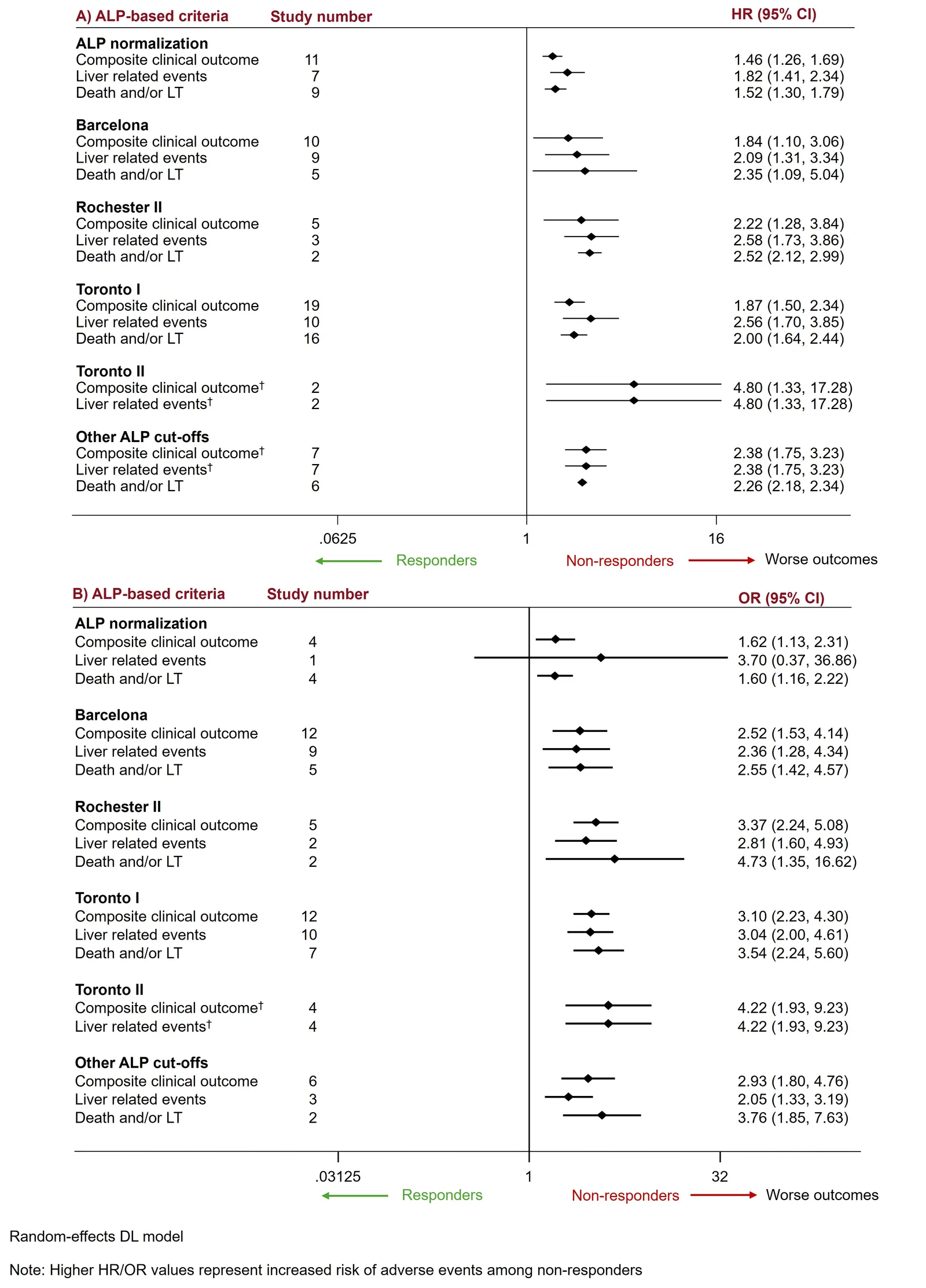
Association Between Failure to Achieve ALP Response and Adverse Long-Term Clinical Outcomes Using Random-Effects Model: (**A**) HR Estimates, (**B**) OR Estimates Abbreviations: ALP, alkaline phosphatase; CI, confidence interval; HR, hazard ratio; LT, liver transplantation; OR, odds ratio. Forest plots showing the pooled association between nonresponse and adverse clinical outcomes according to different ALP-based response criteria. **Panel A** presents hazard ratios (HRs) with 95% confidence intervals (CIs), and **Panel B** presents odds ratios (ORs) with 95% CIs for the association between nonresponse and the risk of adverse outcomes. Diamonds represent pooled effect estimates and horizontal lines indicate 95% CIs. Values to the right of the line of no effect (HR/OR = 1) indicate a greater risk of adverse outcomes among non-responders compared with responders. The number of studies contributing to each estimate is shown in the left column. †The same studies are contributing to the meta-analysis of composite clinical outcomes and liver-related events.

### Other ALP Response Criteria

Further, analysis was also conducted for other ALP response criteria including Toronto I, Barcelona, Rochester II and Toronto II. At the level of individual long-term clinical outcomes, 18 studies reported an association between Barcelona criteria and composite clinical outcomes, 14 studies assessed any liver-related events, and 9 studies evaluated overall death and/or LT. The analyses (non-responders vs responders) reported that responders had a significantly lower risk of composite clinical outcomes (HR, 1.84; 95% CI, 1.10-3.06), any liver-related events (HR, 2.09; 95% CI, 1.31-3.34), and overall death and/or LT (HR, 2.35; 95% CI, 1.09-5.04) compared with non-responders (**Figure 1A**). Consistent findings were reported for the Rochester II, Toronto I, and Toronto II criteria, where patients who achieved response were at a significantly lower risk of long-term clinical outcomes compared with non-responders (**Figure 1A**). Similar findings were reported for studies with categorical data (**Figure 1B**).

Furthermore, 18 studies reported the association between alternative ALP cut-offs and long-term clinical outcomes and were considered eligible for meta-analysis. Compared with responders, non-responders had a significantly higher risk of composite clinical outcomes (HR, 2.38; 95% CI, 1.75-3.23), any liver-related events (HR, 2.38; 95% CI, 1.75-3.23), and overall death and/or LT (HR, 2.26; 95% CI, 2.18- 2.34) (**Figure 1A; Figure 1B**).

### Composite Response

In all, 58 studies were considered for meta-analysis, which reported an association between composite response and long-term clinical outcomes. The most commonly used composite response criteria were Paris I (ALP ≤3 × ULN, aspartate aminotransferase (AST) ≤2 × ULN, and normal bilirubin; n = 31), Paris II (ALP and AST ≤1.5 × ULN and normal bilirubin; n = 29), followed by Rotterdam criteria (normal bilirubin and albumin concentrations; n = 20), Poise (ALP <1.67 × ULN, ≥15% ALP reduction, bilirubin ≤1 × ULN; n = 10), ALP + other composite response (n = 8), Momah/Lindor (ALP < 1.67 × ULN and normal bilirubin; n = 7), modified Toronto criteria (ALP ≤ 1.67 × ULN and total bilirubin ≤ 2 × ULN; n = 2), and Rochester criteria (ALP ≤ 2 x ULN or Mayo score ≤ 4.5 after 6 months of treatment; n = 2).

Among different long-term clinical outcomes, 31 studies reported an association between Paris I criteria and composite clinical outcomes, 21 studies assessed any liver-related events, and 13 studies evaluated overall death and/or LT. The analyses (non-responders vs responders) reported that Paris I responders had a significantly lower risk of composite clinical outcomes (HR, 4.11; 95% CI, 3.08-5.48), any liver-related events (HR, 4.04; 95% CI, 2.79-5.84), and overall death and/or LT (HR, 5.24; 95% CI, 4.02-6.82) compared to non-responders (**Figure 2A**). Consistent findings were reported for Paris II, Poise, modified Toronto, Momah/Lindor, ALP + composite, and Rotterdam criteria where patients who achieved response were at a significantly lower risk of long-term clinical outcomes compared to non-responders (**Figure 2A**).

**Figure 2.**
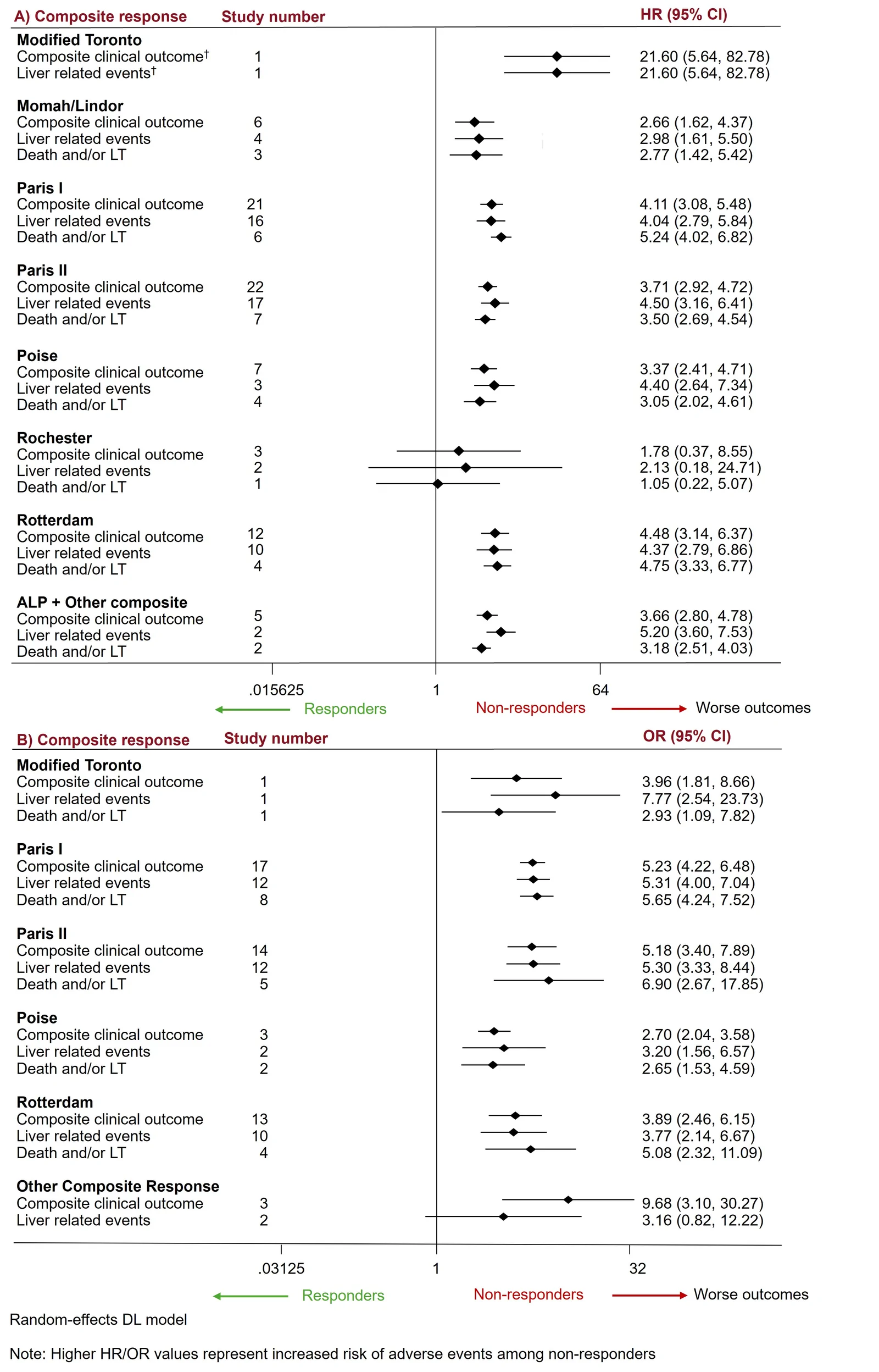
Association Between Failure to Achieve Composite Response and Adverse Long-Term Clinical Outcomes Using Random-Effects Model: (**A**) HR Estimates, (**B**) OR Estimates Abbreviations: ALP, alkaline phosphatase; CI, confidence interval; HR, hazard ratio; LT, liver transplantation; OR, odds ratio. Forest plots showing the pooled association between nonresponse and adverse clinical outcomes according to different composite response criteria. **Panel A** presents hazard ratios (HRs) with 95% confidence intervals (CIs), and **Panel B** presents odds ratios (ORs) with 95% CIs for the association between nonresponse and the risk of adverse outcomes. Diamonds represent pooled effect estimates and horizontal lines indicate 95% CIs. Values to the right of the line of no effect (HR/OR = 1) indicate a greater risk of adverse outcomes among non-responders compared with responders. The number of studies contributing to each estimate is shown in the left column. †The same studies are contributing in the meta-analysis of composite clinical outcomes and liver-related events.

Further, compared with responders according to the Poise criteria, non-responders had a significantly higher risk of composite clinical outcomes (HR, 3.37; 95% CI, 2.41-4.71), any liver-related events (HR, 4.40; 95% CI, 2.64-7.34), and overall death and/or LT (HR, 3.05; 95% CI, 2.02-4.61) (**Figure 2A**).

### Other Surrogate Endpoints

Overall, 18 studies were eligible for meta-analysis, which reported associations of gamma-glutamyl transferase (GGT)-based surrogate endpoints, specifically Ehime’s criteria (70% decrease from baseline level or a normal level of GGT after 6 months of UDCA treatment) and Nara criteria (up to 69% decrease in gamma-glutamyl transpeptidase level from baseline after 1 year of UDCA treatment), bilirubin normalization (1.0 × ULN), Paris IIa (ALP and AST ≤2 x ULN, with normal bilirubin level), Paris IIc (both normal ALP, AST and bilirubin levels), Xi’an 6_a (total bilirubin ≤ 1 × ULN, ALP ≤ 2 × ULN, and AST ≤ 1.5 × ULN), Xi’an 6_b (total bilirubin ≤ 1 × ULN, ALP ≤ 2 × ULN, and AST ≤ 2 × ULN), Xi’an 6_c (total bilirubin ≤ 1 × ULN, ALP ≤ 2.5 × ULN, and AST ≤ 1.5 × ULN) and Xi’an 6_d criteria (ALP ≤ 2.5 × ULN and AST ≤ 2 × ULN, and total bilirubin ≤ 1 × ULN).

Among different long-term clinical outcomes, eight studies reported an association of other surrogate endpoints with composite clinical outcomes. The surrogate endpoints included bilirubin normalization (n = 6), Ehime criteria (n = 2), followed by Paris IIa, Paris IIc, Xi’an criteria, and GGT normalization (n = 1 each). In addition, 13 studies reported associations between these surrogate endpoints and overall death and/or LT.

Pooled estimates (non-responders vs responders) showed that patients who achieved response according to these surrogate endpoints were at a significantly lower risk of both composite clinical outcomes (HR, 3.57; 95% CI, 2.21-5.76) and overall death and/or LT (HR, 3.11; 95% CI, 2.11-4.58) compared with non-responders (**Figure 3A**).

**Figure 3.**
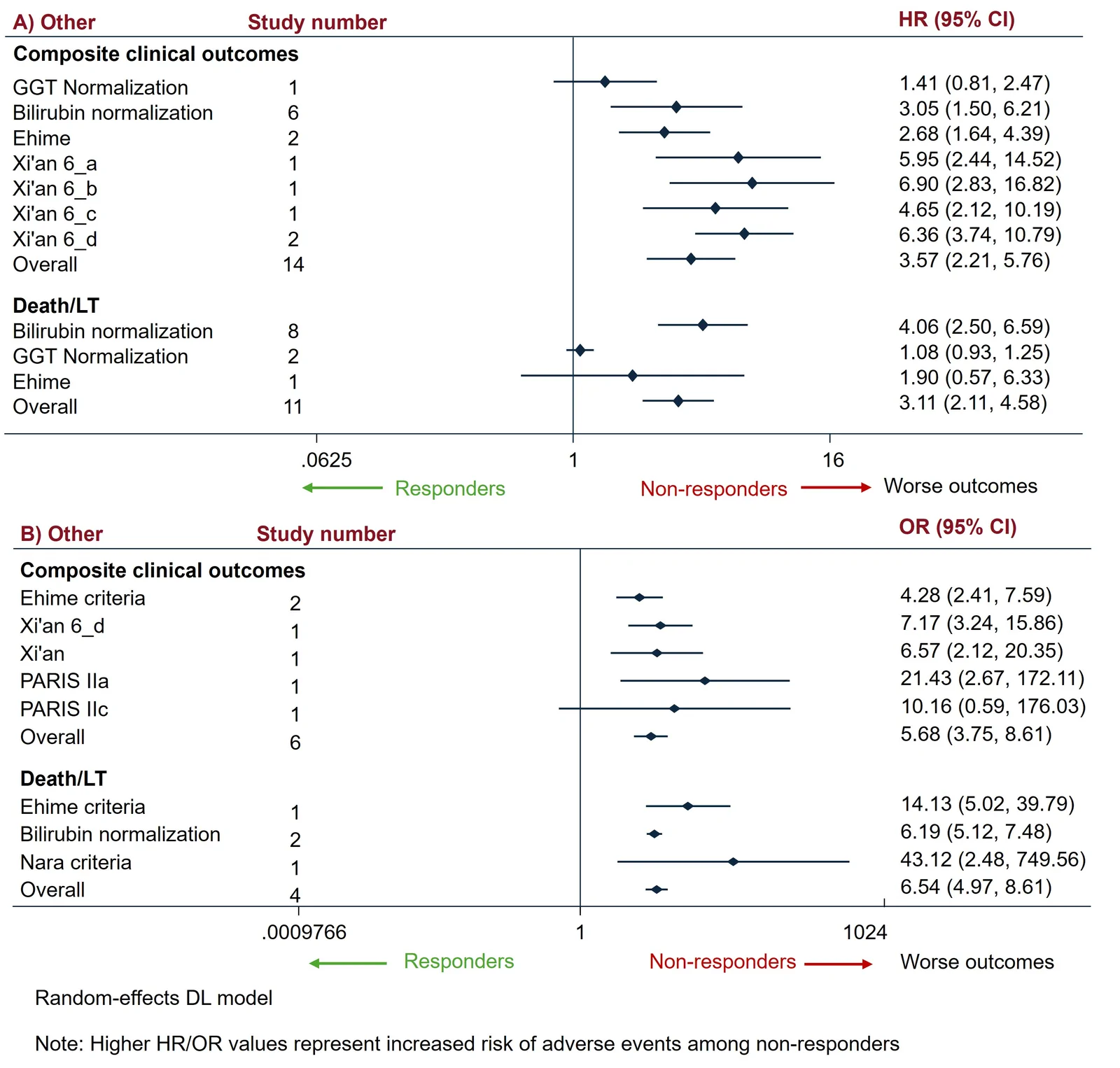
Association Between Failure to Achieve Response (Based on GGT/Ehime/Nara/Bilirubin or other Criteria) and Adverse Long-Term Clinical Outcomes Using Random-Effects Model: (A) HR Estimates, (B) OR Estimates Abbreviations: CI, confidence interval; GGT, gamma-glutamyl transferase; HR, hazard ratio; LT, liver transplantation; OR, odds ratio. Forest plots showing pooled associations between nonresponse and adverse outcomes according to response criteria other than conventional ALP-based and composite response definitions. **Panel A** presents hazard ratios (HRs) with 95% confidence intervals (CIs) for time-to-event analyses, whereas **Panel B** presents odds ratios (ORs) with 95% CIs for binary outcome analyses. Diamonds represent pooled effect estimates and horizontal lines indicate 95% CIs. Values to the right of the line of no effect (HR/OR = 1) indicate a greater risk of adverse outcomes among non-responders compared with responders. The number of studies contributing to each estimate is shown in the left column.

Further, analyses were conducted to examine the association of other surrogate endpoints specifically in relation to liver-related death (**Figure S7 and S8)**.

### Sensitivity Analyses

Detailed results of the sensitivity analyses are provided in **Supplementary Figures S4-S6 and Supplementary Tables S9-S15**. The findings were generally consistent with the base-case analysis, showing a numerically lower risk of adverse long-term clinical outcomes among patients who achieved ALP response, ALP normalization, composite response, or other response criteria than among non-responders. Most analyses demonstrated statistically significant associations, although significance was not reached in some comparisons.

### Publication Bias

Overall, visual inspection of the funnel plots demonstrated no asymmetrical distributions across analyses (**Supplementary Figures S9 and S10).** Evidence of publication bias was not detected in the majority of analyses (*P* ≥ .5), except for the association between composite response and any liver-related event (*P* = .0031) and association of composite response with composite clinical outcomes (*P* = .0002).

## DISCUSSION

This SLR and meta-analysis provides a comprehensive global assessment of the association between biochemical surrogate endpoints and long-term clinical outcomes in PBC, supporting their prognostic value and relevance for clinical and HTA decision-making. Although UDCA is the standard first-line therapy for PBC, approximately 40% of patients have an inadequate biochemical response (ALP ≥1.67× ULN and/or abnormal serum total bilirubin) to therapy, and consequently remain at increased risk of disease progression.^6,7^ Notably, an even larger proportion of patients fail to achieve complete ALP normalization, which is increasingly recognized as a stringent treatment goal in PBC.^8^ Given the slow and heterogeneous progression of PBC, reliance on hard clinical endpoints such as LT or mortality poses significant challenges for timely evaluation of treatment efficacy. This creates an important unmet evidence need, as regulatory, HTA, reimbursement, and clinical decision-making often must occur before mature long-term outcome data become available. In this context, surrogate endpoints help address an important evidence gap in PBC by enabling earlier assessment of therapeutic benefit, thereby supporting clinical, regulatory, HTA, and reimbursement decision-making when mature long-term outcome data are not yet available.^2,46^

Although individual studies have suggested associations between markers such as ALP, bilirubin, and composite biochemical response criteria and outcomes including LT, mortality, and disease progression, the strength, consistency, and generalizability of these relationships have not been comprehensively evaluated across diverse populations and study designs. A rigorous SLR and meta-analysis was therefore warranted to identify, evaluate, and summarize the totality of available evidence, quantify the magnitude of these associations, and assess the robustness of surrogate endpoints as predictors of long-term clinical outcomes in PBC.

In the base-case analysis, statistically significant associations were identified between ALP normalization, ALP response, composite response, bilirubin normalization and long-term clinical outcomes. Sensitivity analyses were consistent with the base-case results in most analyses, supporting the robustness and reliability of the primary findings. These findings matter because they directly address one of the central evidence challenges in PBC, where definitive clinical outcomes such as LT, hepatic decompensation, and death often require many years to occur, making them difficult to assess within the time frame of conventional clinical trials.

The novelty of the current meta-analysis lies in its comprehensive, quantitative synthesis of multiple biochemical surrogate endpoints across a broad spectrum of long-term clinical outcomes and a global evidence base. Prior evidence has established the prognostic relevance of ALP and bilirubin, particularly for transplant-free survival and death/LT; however, earlier analyses were more focused on selected endpoints, specific cohorts, or narrower clinical outcome definitions.^47^ By contrast, this SLR and meta-analysis integrates evidence for ALP normalization, other ALP response criteria, bilirubin normalization, and composite biochemical response definitions across composite clinical outcomes, any liver-related events, overall death and/or LT, and other secondary outcomes. This broader synthesis supports the conclusion that these measures are not simply laboratory markers, but clinically interpretable predictors of downstream disease progression. By consolidating evidence across diverse populations, study designs, and outcome definitions, the analysis reduces uncertainty for decision-makers who must evaluate treatments in the absence of mature long-term outcome data.

Our findings are aligned with those reported in the meta-analysis conducted by the Global PBC Study Group, which demonstrated that baseline ALP and bilirubin levels are strongly associated with risk of LT and death, with higher levels associated with worse clinical outcomes.^2^ However, the Global PBC Study Group analysis was limited to long-term follow-up cohorts from North America and Europe, whereas the present meta-analysis incorporates evidence from a global population, thereby enhancing the generalizability of the findings. Further, similar to our analysis, Lammers et al demonstrated that ALP and bilirubin are robust surrogate endpoints in PBC, showing strong associations with long-term transplant-free survival in a large cohort of 4845 patients followed for a median of 7.3 years. This study demonstrated that ALP and bilirubin levels, assessed at enrolment and annually over a 5-year period, were strongly associated with long-term clinical outcomes. The combined assessment of both ALP and bilirubin levels was the strongest predictor of transplant-free survival duration. In the same study, ALP and bilirubin were also shown to be individually predictive of transplant-free survival.^2^

For regulatory bodies, the current findings are consistent with, but do not independently establish, the validation of ALP- and bilirubin-based endpoints as formal regulatory surrogates for treatment effect; such validation additionally requires trial-level evidence that treatment-induced changes in these measures reliably predict the corresponding treatment effect on clinical outcomes. Regulatory authorities already recognize these biomarkers as components of composite endpoints that can support accelerated approval, while ongoing long-term confirmatory outcome studies remain essential to establish long-term clinical benefit and support full regulatory approval. In this context, the present meta-analysis should be viewed as complementary to ongoing post-approval and confirmatory evidence generation, rather than as a substitute for these studies.

In addition to established biochemical response criteria, increasing attention has been placed on achieving complete ALP normalization as a treatment goal in PBC. Recent expert consensus recommendations^15^ have highlighted ALP normalization as a stringent therapeutic goal, given accumulating evidence demonstrating its association with improved long-term clinical outcomes, including reduced risks of LT and death. The findings of the present meta-analysis further support the clinical relevance of ALP normalization, with patients achieving normalization consistently demonstrating lower risk of adverse outcomes than those with persistently elevated ALP levels. The consistency of the present pooled estimates with these positions reinforces the rationale for using ALP normalization as an endpoint in phase III trials, while also emphasizing the importance of continued post-approval evidence generation.

For HTA bodies and payers, the findings provide a more decision-relevant evidence base for interpreting biochemical response as an indicator of long-term clinical and economic value; this relevance lies in reducing uncertainty in the interpretation and modeling of biochemical response as a link to longer-term outcomes, rather than in providing definitive evidence that biochemical improvements can be directly converted into a specific magnitude of long-term clinical or economic benefit.

HTA decisions often require judgment about whether short-term trial outcomes are sufficiently linked to final patient-relevant outcomes, particularly in rare or slowly progressive diseases. By quantifying the association between biochemical nonresponse and higher risks of composite clinical outcomes, liver-related events, overall death and/or LT, this meta-analysis helps reduce uncertainty around the extent to which improvements in ALP, bilirubin, and composite response criteria can be used to inform, rather than substitute for, assumptions about long-term benefit in economic and decision models. This is especially important for reimbursement assessments of emerging second-line PBC therapies, where available trial evidence commonly relies on changes in ALP, bilirubin, and composite biochemical response rather than long-term clinical events; in this setting, the present findings can help contextualize and support, but should not replace, therapy-specific evidence and modeling assumptions. For clinicians, these results reinforce the practical value of routine biochemical monitoring for risk stratification and treatment optimization.

To our knowledge, this research represents the first comprehensive meta-analytic evaluation integrating multiple surrogate biochemical endpoints with a broad spectrum of long-term clinical outcomes in PBC across a global study population. By synthesizing evidence from diverse geographic regions, study designs, and follow-up durations, this analysis provides evidence supporting the prognostic value of established biochemical measures as predictors of clinically meaningful outcomes. However, given the predominantly observational evidence base, heterogeneity in response and outcome definitions across studies, inclusion of conference abstracts, and the possibility of overlapping patient cohorts, prognostic association alone is not sufficient to establish these measures as validated surrogates for treatment effect; such validation would require confirmatory evidence linking treatment-induced changes in the surrogate with treatment effects on the corresponding clinical outcome. Importantly, this meta-analysis encompasses surrogate endpoints endorsed by regulatory authorities such as the EMA and FDA, enhancing the clinical and regulatory relevance of the findings. The strengths of this meta-analysis include rigorous adherence to PRISMA guidelines, ensuring a systematic and transparent approach to data synthesis. Application of the DL random-effects model accommodates heterogeneity by allowing true effect sizes to vary across studies and by incorporating between-study variance (τ^2^), which is particularly important in the presence of clinical and methodological diversity. Additionally, the MH method provides a well-established, robust estimator for fixed-effect pooling of OR, particularly when studies vary in sample size.^15^ Model-based sensitivity analyses using the IV FEM and DL + HKSJ random-effects approaches confirmed the base-case findings.

The current meta-analysis has certain limitations. First, the evidence base was predominantly observational; only three RCTs were identified, with the remainder comprising prospective or retrospective observational cohorts, which limits causal inference and means that residual confounding and selection bias cannot be excluded. Second, it included studies with differences in design, sample size, geographic setting, patient characteristics, composite endpoint definitions, and follow-up duration, which might introduce heterogeneity and limit generalizability. Third, the data quality and reporting standards across the included studies varied, potentially impacting the reliability of the meta-analysis results. Further, some surrogate definitions (eg, Poise, Rochester, Rotterdam) were assessed in only a few studies, which reduced the robustness and statistical power of the pooled estimates. The majority of included studies involved patients treated with first-line UDCA alone or in combination with fibrates. Direct long-term outcome data for recently approved PPAR-targeted therapies remain limited because of their relatively short duration of clinical use^15^; however, this does not negate the relevance of the findings for contemporary therapies. Additionally, although study quality was assessed using the NOS checklist, this tool was developed primarily for case-control and cohort studies of exposure-outcome associations and has recognized limitations when applied to prognostic-factor studies, as it may not fully capture time-varying exposure, immortal-time bias, residual confounding, missing data, or the validity of the analytic model used.^48,49^ The surrogate endpoints evaluated in this analysis are treatment-agnostic biochemical markers of cholestatic disease activity and hepatic function, and they are the same endpoints used in modern PBC trials and regulatory assessments. Lastly, the current analysis focused on routine biochemical parameters that have long been used in the diagnosis and management of PBC, as these measures are likely to be the most readily applicable in routine clinical practice. However, future studies should also evaluate other potential markers, including liver stiffness measures (eg, transient elastography or magnetic resonance elastography) and other validated non-invasive biomarkers for assessing fibrosis and long-term disease progression in PBC.

## CONCLUSION

This SLR and meta-analysis underscores the prognostic role of surrogate endpoints, such as ALP normalization, ALP response, composite response criteria, and bilirubin levels, in predicting significant clinical outcomes in PBC. The consistent associations observed across diverse studies support the prognostic and decision-relevant value of these measures as indicators of risk for adverse outcomes, including LT, death, and disease progression. Importantly, these endpoints are also being used in clinical trials of second-line PBC therapies such as elafibranor and seladelpar, where changes in ALP and bilirubin and related composite response definitions commonly serve as key efficacy outcomes. Collectively, these findings highlight the potential for these markers to support clinical, regulatory, and HTA decision-making by reducing uncertainty in the interpretation and modeling of biochemical response in relation to long-term clinical and economic outcomes, and to improve the management of PBC by providing early insights into patient prognosis.

### Disclosures

D.M., M.R., C.K., and R.T. are employees of Gilead Sciences, Inc., and may own stock in Gilead Sciences, Inc. P.R. and B.S. are employees of Pharmacoevidence.

## Supporting information

Online Supplementary Material

Graphical Abstract

## Data Availability

The data supporting the findings are available in the supplementary material accompanying the article.
