# Supplementary material for: Relationship of Biochemical Response as Surrogate Endpoints with Long-Term Outcomes in Primary Biliary Cholangitis: A Global Systematic Review and Meta-Analysis
Source: J Health Econ Outcomes Res. 2026 Oct 30;13(2):203–12. doi: 10.36469/001c.170737 (PMC13724029; doi:10.36469/001c.170737)
Supplement: Supplementary file 1 [file jheor_2026_13_2_170737_368318.pdf]

Relationship of Biochemical Response as Surrogate Endpoints with Long-Term Outcomes in Primary Biliary Cholangitis: A Global Systematic Review and Meta-Analysis

Supplementary Material

Figure S1: PRISMA Diagram for the SLR

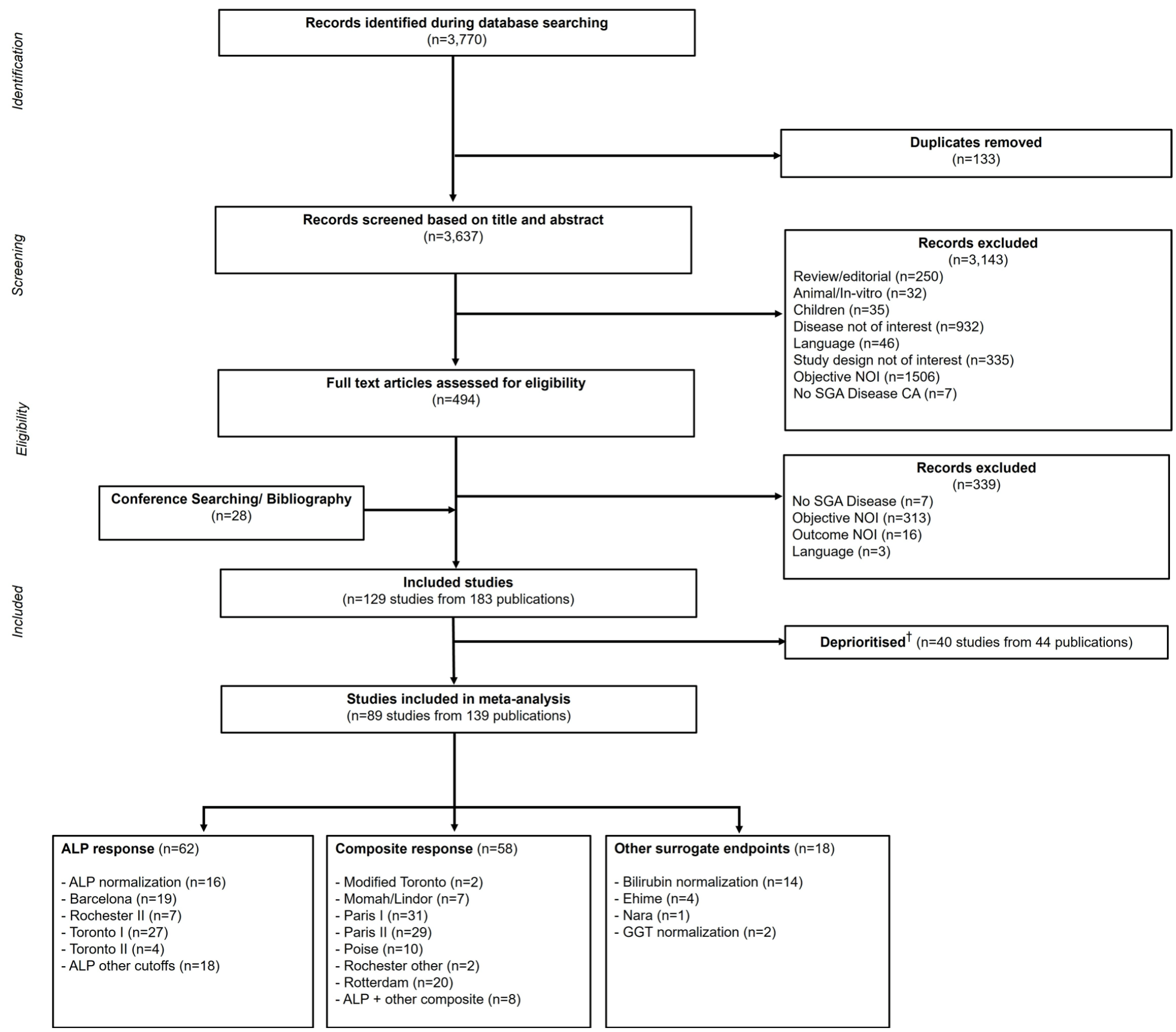

NOI: Not of interest; PRISMA: Preferred Reporting Items for Systematic Reviews and Meta-Analysis; SGA: Subgroup available; SLR: Systematic Literature review
†Not meeting meta-analysis eligibility criteria
Note: The endpoint categories are not mutually exclusive.

Table S1: Search Strategy for Embase® and MEDLINE® Using Embase.com†

#	SEARCH SYNTAX
1	'primary biliary cirrhosis'/exp OR 'primary biliary cirrhosis'/syn OR 'primary biliary cholangitis' OR 'primary biliary cirrhosis' OR 'biliary cirrhosis, primary' OR 'primary biliary cholestasis' OR 'biliary liver cirrhosis' OR pbc:ab,ti
2	'liver cell carcinoma'/exp OR 'liver transplant-free survival' OR 'liver transplant free survival' OR 'liver transplant*' OR 'disease progression' OR 'event-free survival' OR 'event free survival' OR 'cirrhosis':ab,ti OR 'compensat* cirrhosis':ab,ti OR death:ab,ti OR mortality:ab,ti OR 'liver outcome*':ab,ti OR 'liver related outcome*':ab,ti OR 'liver related complication*':ab,ti OR 'liver stiffness':ab,ti OR 'liver fibrosis':ab,ti
3	progn*:ab,ti OR 'risk factor*':ab,ti OR 'natural histor*' OR progress*:ab,ti OR 'surrogate':ab,ti
4	'alp level*':ab,ti OR 'biochemical respons*':ab,ti OR 'alp normali*':ab,ti OR 'alkaline phosphatase level*':ab,ti OR 'alkaline phosphatase normali*':ab,ti OR 'alkaline phosphatase respons*':ab,ti OR 'alkaline phosphatase estima*':ab,ti OR 'alp estima*':ab,ti OR 'alt level*':ab,ti OR 'alt normali*':ab,ti OR 'alanine transaminase level*':ab,ti OR 'alanine transaminase normali*':ab,ti OR 'alanine transaminase respons*':ab,ti OR 'alanine transaminase estima*':ab,ti OR 'alt estima*':ab,ti OR 'ast level*':ab,ti OR 'ast normali*':ab,ti OR 'aspartate transaminase level*':ab,ti OR 'aspartate transaminase normali*':ab,ti OR 'aspartate transaminase respons*':ab,ti OR 'aspartate transaminase estima*':ab,ti OR 'ast estima*':ab,ti OR 'serum bilirubin level':ab,ti OR bilirubin:ab,ti OR 'bilirubin level':ab,ti OR hyperbilirubi*:ab,ti OR hypobilirubi*:ab,ti OR 'igm level*':ab,ti OR 'igm estima*':ab,ti OR 'fibrosis markers' OR 'markers of fibrosis' OR 'fibrotest':ab,ti OR 'elastography'
5	'cohort study' OR cohort* OR (('follow up' OR 'followup') NEXT/1 ('study' OR 'studies')) OR 'retrospective study'/exp OR 'cohort analysis'/exp OR 'longitudinal study'/exp OR 'prospective study'/exp OR 'observational study' OR (cohort NEXT/1 stud*) OR (cohort NEXT/1 analy*) OR 'register' OR 'registry' OR ('database' NEAR/2 'study') OR ('real' NEXT/1 'world') OR ('healthcare' NEXT/1 'record') OR 'pragmatic trial' OR 'real-world clinical trial' OR 'pragmatic clinical trial' OR 'retrospective' OR 'prospective' OR 'database' OR 'retrospective observational' OR 'prospective observational' OR 'non-random' OR 'nonrandom' OR 'nrct' OR 'n rct' OR n?rct OR (('non' OR 'not') NEAR/3 ('randomised' OR 'randomized'))

#	SEARCH SYNTAX
6	#2 OR #3 OR #4
7	#1 AND #5 AND #6

[†]Similar search strategy was followed for PubMed and Cochrane

Table S2: Participants, Interventions, Comparators, Outcomes, Timeframe, and Study Design for Inclusion in the SLR

INCLUSION CRITERIA	EXCLUSION CRITERIA
**POPULATION**	
Adult patients (aged 18 to 85 years) diagnosed with PBC, irrespective of diagnostic criteria and measures, gender, race, and ethnicity	Patients with diseases other than PBC<br>Patients <18 years old<br>Mixed populations for which outcomes for adults with PBC are not presented separately<br>Assessment of patients with PBC with other comorbidities (e.g., HIV, cancer, etc.)
**INTERVENTION/ COMPARATOR**	
No restriction	NA
**OUTCOMES**	
Surrogate endpoints:<br>• ALP response, ALP normalization, other ALP cut-offs<br>• Composite response<br>• Other surrogate endpoints<br>Long-term clinical outcomes:<br>• Composite clinical outcomes[†]<br>• Overall death and/or LT<br>• Any liver-related events<br>• Other secondary outcomes (graft loss after LT, overall mortality after LT, recompensation,	Studies that do not report data of interest

INCLUSION CRITERIA	EXCLUSION CRITERIA
disease progression)	
**STUDY DESIGN**	
• Clinical trials<br>• Observational studies<br>• Case-control studies<br>• Real-world cohort studies<br>• Database studies	• Case reports<br>• Letters<br>• Case series
**LANGUAGE**	
• English language only. English abstracts of non-English language studies were considered for inclusion	• Studies published in languages other than English

ALP: Alkaline Phosphatase; HCC: Hepatocellular Carcinoma; LT: Liver Transplantation; NA: Not Applicable; PBC: Primary Biliary Cholangitis; SLR: Systematic Literature Review

[†]Includes major clinical events, disease-related complications, overall death and/or LT

Table S3: Study Characteristics of the Included Evidence

S.No.	Study name	Publication Type	Study design	Country	Sample size	Treatment details	Study duration (months)
1	Abbas 2026[γ]	Journal article	Prospective observational study	Global	336	1L: UDCA (91) 2L: OCA (100)	24; Follow-up: 48
2	Ampuero 2024	Journal article	Prospective and retrospective observational	Spain	388	1L: UDCA (100) 2L: OCA + UDCA (26.8), Fibrates + UDCA (24)	Follow-up for UDCA: 114‡; for OCA: 25.2‡; for fibrates-32.4‡
3	Angulo 1999	Journal article	RCT	USA	180	1L: UDCA (49.4) Placebo (50.6)	51; Extended follow-up for UDCA: 55.2†; for placebo: 27.6†
4	Arvaniti 2025	Conference Abstract	Retrospective observational study	Spain	505	1L: UDCA (NR) 2L: Bezafibrate or OCA (100)	Follow-up: 110†
5	Azemoto 2009	Journal article	Retrospective observational	Japan	83	1L: UDCA (100) 2L: Bezafibrate + UDCA (18)	216; Follow-up: 62.1‡

S.No.	Study name	Publication Type	Study design	Country	Sample size	Treatment details	Study duration (months)
6	Azemoto 2011a[g]	Journal article	Retrospective observational	Japan	138	1L: UDCA (100)	382; Follow-up: 55.5†
7	Bonnand 1999	Journal article	RCT	Global	548	1L: UDCA (49.8) Placebo (50.2)	NR
8	Carbone 2013	Conference abstract	Retrospective observational	UK	3567	1L: UDCA (100)	NR
9	Carbone 2015a	Conference abstract	Prospective observational	Global	8867	1L: UDCA (77.7)	Follow-up for UK-PBC cohort: 79.2†; for global PBC cohort: 87.6†
10	Carbone 2016	Journal article	Prospective observational	UK	3919	1L: UDCA (80.7)	79; Follow-up: 75.6†
11	Chang 2023	Journal article	Retrospective observational	South Korea	173	1L: UDCA (100)	120; Follow-up: 78†
12	Chen 2026[β]	Journal article	Retrospective observational study	China	553	1L: UDCA (100)	252; Follow-up: 65†

S.No.	Study name	Publication Type	Study design	Country	Sample size	Treatment details	Study duration (months)
13	Cheung 2016	Journal article	Retrospective observational	Canada	120	1L: UDCA (100) 2L: Fenofibrate + UDCA (38.3)	300; Follow-up for fenofibrate: 58.8‡; for UDCA: 46.8‡
14	Cheung 2017	Conference abstract	Retrospective observational	China	144	1L: UDCA (100)	192; Follow-up: 84†
15	Copaci 2011	Conference abstract	Prospective observational	Romania	62	1L: UDCA (100)	NR
16	Corpechot 2008[a]	Journal article	Prospective observational	France	292	1L: UDCA (100)	NR
17	Corpechot 2016	Conference abstract	Retrospective observational	Global	176	1L: UDCA (100)	Follow-up: 67.2
18	Corpechot 2022[c]	Journal article	Prospective observational	Global	3308	1L: UDCA (98.3) 2L: OCA (7.6), Fibrates (13.7)	25; Follow-up for derivation cohort: 60‡; for validation cohort: 60‡
19	Couto 2026	Conference abstract	Retrospective observational study	Global	2182	1L: UDCA (96.9) 2L: Fibrates (27.8), OCA (0.3)	Follow-up: 88.8

S.No.	Study name	Publication Type	Study design	Country	Sample size	Treatment details	Study duration (months)
20	de Tijera 2025[θ]	Conference abstract	Case-control study	Mexico	329	1L: UDCA (100) 2L: Fibrate (17), Statin (15.2)	NR
21	de Veer 2022	Journal article	Retrospective observational	Global	3774	1L: UDCA (100)	Follow-up: 86.4†
22	De Vincentis 2024[j]	Conference abstract	Retrospective observational	Italy	631	1L: UDCA (NR) 2L: OCA (100)	Follow-up: 30†
23	Ding 2025a	Journal article	Retrospective observational study	China	526	1L: UDCA (100) 2L: UDCA + Fenofibrate (19.4)	100; Follow-up: 34†
24	Ding 2025b	Journal article	Retrospective observational study	China	712	1L: UDCA (100) 2L: Fenofibrate + UDCA (24)	192; Follow-up: 68‡
25	Ding 2025c	Journal article	Retrospective observational study	China	170	1L: UDCA (100)	208; Follow-up: 39†
26	Dorizas 2025	Conference Abstract	Retrospective observational study	USA	84	1L: UDCA (100)	NR

S.No.	Study name	Publication Type	Study design	Country	Sample size	Treatment details	Study duration (months)
27	Fujinaga 2017	Conference abstract	Retrospective observational	Unclear	35	1L: UDCA (100)	NR
28	Fujinaga 2019a[f]	Journal article	Retrospective observational	Japan	35	1L: UDCA (100)	252; Follow-up: 165.6‡
29	Fujinaga 2025	Journal article	Retrospective observational study	Japan	226	1L: UDCA (100)	129; Follow-up: 114‡
30	Gatselis 2026	Conference Abstract	Retrospective observational study	Greece	1385	1L: UDCA (100)	Follow-up: 84†
31	Gazda 2021[d]	Journal article	Retrospective observational	Global	249	1L: UDCA (100)	241; Follow-up: 60†
32	Gerussi 2020[l]	Conference abstract	Prospective observational	Global	2129	1L: UDCA (93.1)	Follow-up: 120
33	Gish 2025	Conference Abstract	Retrospective observational study	USA	726	1L: UDCA (100)	73
34	Gish 2026	Conference Abstract	Retrospective observational study	USA	4013	1L: UDCA (100)	54

S.No.	Study name	Publication Type	Study design	Country	Sample size	Treatment details	Study duration (months)
35	Haddadin 2019	Conference abstract	Retrospective observational	UK	68	1L: UDCA (100)	Follow-up: 35‡
36	Haldar 2021[k]	Journal article	Retrospective observational	UK	499	1L: UDCA (NR)	204
37	Harms 2015	Conference abstract	Retrospective observational	Global	2326	1L: UDCA (100)	Follow-up: 109.2†
38	Harms 2019[q]	Journal article	Retrospective observational	Global	3902	1L: UDCA (90.4)	Follow-up: 93.6†
39	Hayashi 2024	Journal article	Retrospective observational study	Japan	119	1L: UDCA (100) 2L: Bezafibrate + UDCA (27.7)	372; Follow-up: 84†
40	Hernández 2024a	Conference abstract	Retrospective observational study	Spain	400	1L: UDCA (NR) 2L: Bezafibrate (100) 3L: OCA (15.5)	NR
41	Hernández 2024b	Conference Abstract	Retrospective observational study	Spain	215	1L: UDCA (NR) 2L: Fibrates (90.6), OCA (9.3)	Follow-up: 56.4†
42	Hofer 2024	Journal article	Retrospective observational	Austria	42	1L: UDCA (95.2) 2L: Fibrates (14.3)	216; Follow-up: 41.9†

S.No.	Study name	Publication Type	Study design	Country	Sample size	Treatment details	Study duration (months)
43	Huang 2017	Conference abstract	Prospective observational	China	74	1L: UDCA (100)	Follow-up: 36†
44	Huang 2018[i]	Conference abstract	Prospective observational	China	119	1L: UDCA (100)	Follow-up: 45.6†
45	Huet 2008	Journal article	Prospective observational	Canada	132	1L: UDCA (100)	154; Follow-up: 103.68‡
46	Iwadare 2024	Journal article	Retrospective observational study	Japan	190	1L: UDCA (100)	344; Follow-up: 116.4†
47	John 2021a	Journal article	Retrospective observational	USA	105	1L: UDCA (100) 2L: OCA + UDCA (20)	108; Follow-up: 39
48	John 2021b	Journal article	Retrospective observational	USA	532	1L: UDCA (94.2)	108; Follow-up: 42
49	Jorgensen 1995	Journal article	RCT	USA	180	1L: UDCA (49.4) Placebo (50.6)	Follow-up: 24
50	Kimura 2021	Journal article	Retrospective observational	Japan	196	1L: UDCA (100) 2L: Bezafibrate (8.2), Prednisolone (9.7)	432; Follow-up: 12

S.No.	Study name	Publication Type	Study design	Country	Sample size	Treatment details	Study duration (months)
51	Koky 2025a[α]	Journal article	Retrospective observational study	Slovakia	155	1L: UDCA (100) 2L: Fenofibrate (25.5), OCA (4.1)	312; Follow-up: 120†
52	Kowdley 2024[w]	Conference abstract	Retrospective observational study	USA	22487	1L: UDCA (84.5) 2L: OCA (8.4), UDCA + OCA (7)	73
53	Kowdley 2025	Journal article	Retrospective observational study	USA	3974	1L: UDCA (49.9)	92; Follow-up: 30†
54	Kugiyama 2025	Journal article	Prospective observational study	Japan	1283	NR	Follow-up: 46.8†
55	Kuiper 2009a[b]	Journal article	Prospective observational	Netherlands	375	1L: UDCA (100)	208; Follow-up: 116.4†
56	Kumagi 2010a	Journal article	Retrospective observational	Canada	69	1L: UDCA (95.7)	Follow-up: 98.3‡
57	Kumagi 2010b	Conference abstract	Retrospective observational	USA	683	1L: UDCA (100)	420; Follow-up: 98.28‡
58	Lam 2023	Conference abstract	Retrospective observational	Global	2244	1L: UDCA (100)	Follow-up: 180

S.No.	Study name	Publication Type	Study design	Country	Sample size	Treatment details	Study duration (months)
59	Lammers 2013a	Conference abstract	Retrospective observational	Global	2111	1L: UDCA (89)	92.4; Follow-up: 9‡
60	Lammers 2013b	Conference abstract	Retrospective observational	Global	3895	1L: UDCA (87)	Follow-up: 84†
61	Lammers 2016a	Journal article	Retrospective observational	Netherlands	687	1L: UDCA (100)	278; Follow-up: 105.6†
62	Lammers 2016b	Conference abstract	Retrospective observational	Global	1820	1L: UDCA (100)	Follow-up: 88.8†
63	Lammers 2018	Conference abstract	Retrospective observational	Global	4820	1L: UDCA (84.9)	NR
64	Lammert 2014	Journal article	Retrospective observational	USA	398	1L: UDCA (100)	75; Follow-up for Toronto responders: 84†; for Toronto non-responders: 99†
65	Leeman 2014	Conference abstract	Prospective and retrospective observational	Netherlands	851	1L: UDCA (100)	Follow-up: 106.8†

S.No.	Study name	Publication Type	Study design	Country	Sample size	Treatment details	Study duration (months)
66	Li 2025	Journal article	Prospective observational study	China	63	1L: UDCA (100) 2L: Fenofibrate + UDCA (100)	109; Follow-up: 45†
67	Lim 2025	Journal article	Prospective observational study	Korea	4882	1L: UDCA (94.6) 1L + : Fibrates (0.9), Glucocorticoid (13.1), Non-glucocorticoid immunosuppressive agent (6.9)	Nationwide cohort: 168; Validation cohort: 202; Follow-up for nationwide cohort: 65.04†; for validation cohort: 66†
68	Lin 2019	Journal article	Retrospective observational	Taiwan	150	1L: UDCA (100)	Follow-up: 360
69	Liu 2016	Journal article	Retrospective observational	China	455	1L: UDCA (95.3)	84
70	Meloni 2024	Conference abstract	Cross sectional	USA	822	1L: UDCA (100)	290

S.No.	Study name	Publication Type	Study design	Country	Sample size	Treatment details	Study duration (months)
71	Miura 2025	Conference abstract	Retrospective observational study	Japan	226	1L: UDCA (100) 2L: Bezafibrate + UDCA (100)	24; After bezafibrate initiation Follow-up: 104.4‡
72	Momah 2012[s]	Journal article	Retrospective observational	USA	73	1L: UDCA (100)	Follow-up: 36
73	Montano-Loza 2024[u]	Journal article	Retrospective observational	Global	332	1L: UDCA (94) 2L: Fibrates (6), OCA (1)	439; Follow-up: 104.4†
74	Murillo 2018	Conference abstract	Retrospective observational	Global	2081	1L: UDCA (100)	Follow-up: 84†
75	Murillo 2019[e]	Journal article	Retrospective observational	Global	1828	1L: UDCA (100)	420; Follow-up: 100.8†
76	Murillo 2020a[o]	Journal article	Retrospective observational	Global	2555	1L: UDCA (91.7)	Follow-up: 87.6†
77	Murillo 2020b	Conference abstract	Prospective observational	Global	234	1L: UDCA (100)	NR
78	Nakamura 2015	Journal article	Prospective and	Japan	287	1L: UDCA (100)§ 2L: Bezafibrate + UDCA (20.7)§	50; Follow-up: 61.5†

S.No.	Study name	Publication Type	Study design	Country	Sample size	Treatment details	Study duration (months)
			retrospective observational			Other: Prednisolone + UDCA ± bezafibrate (9.1)§	
79	Namisaki 2017	Journal article	Retrospective observational	Japan	99	1L: UDCA (100) 2L: Bezafibrate + UDCA (NR)	288; Follow-up: 133.2‡
80	Ohfuji 2026	Conference Abstract	Retrospective observational study	Japan	558	1L: UDCA (100) 2L: Bezafibrate (61.6), Pemafibrate (38.3)	276; Follow-up: 59†
81	Okumura 2025	Journal Article	Retrospective observational study	Japan	274	1L: UDCA (NR) 2L: Bezafibrate (17.1)	517; Follow-up: 105.6
82	Örnolfsson 2019[h]	Journal article	Retrospective observational	Iceland	222	1L: UDCA (60.8) 2L: OCA (2.3)	300
83	Osman 2021[n]	Journal article	Retrospective observational	USA	538	1L: UDCA (80.35)	145
84	Padilla-Lopez 2026	Conference Abstract	Cross-sectional observational study	Global	126	NR	NR

S.No.	Study name	Publication Type	Study design	Country	Sample size	Treatment details	Study duration (months)
85	Papastergiou 2013	Journal article	Prospective observational	UK	215	1L: UDCA (40)	300; Follow-up: 96†
86	Parés 2006	Journal article	Prospective observational	Spain	192	1L: UDCA (100)	NR
87	Pariente 2021	Journal article	Retrospective observational	Global	436	1L: UDCA (96) 2L: Fibrates (11.2), OCA (0.5), Immunosuppressive and/or steroids (14.9)	21; Follow-up: 62.4†
88	Patanwala 2010a[m]	Conference abstract	Prospective observational	UK	94	NR	Follow-up: 120
89	Perez 2018[t]	Conference abstract	Retrospective observational	Global	Baseline: 2795; 1 year: 2967; 3 years: 1596; 5 years: 1274	1L: UDCA (NR)	Follow-up: 60

S.No.	Study name	Publication Type	Study design	Country	Sample size	Treatment details	Study duration (months)
90	Perez 2021	Conference Abstract	Retrospective observational study	Global	573	1L: UDCA (100)	252
91	Pinyopornpanish 2022[p]	Journal article	Retrospective observational	USA	292	1L: UDCA (100) 2L: OCA (NR)	240; Follow-up: 85.2†
92	Qian 2020	Journal article	Prospective observational	China	80	1L: UDCA (100) 2L: UDCA (47.5), UDCA + Prednisolone + Immunosuppressant (52.5)	61; Follow-up: 41.3‡
93	Qu 2019	Journal article	Retrospective observational study	China	65	NR	120; Follow-up: 35†
94	Rachel 2026	Conference Abstract	Retrospective observational study	USA	1137	1L: UDCA (100)	124.5
95	Reddy 2024[x]	Conference abstract	Retrospective observational study	USA	232	1L: UDCA (100)	Follow-up: 60

S.No.	Study name	Publication Type	Study design	Country	Sample size	Treatment details	Study duration (months)
96	Reiberger 2019	Conference abstract	Prospective observational	Austria	382	NR	166
97	Reig 2018	Journal article	Prospective observational	Spain	48	1L: UDCA (100) 2L: Bezafibrate + UDCA (100)	38†
98	Reig 2019	Conference abstract	Prospective observational	Spain	54	1L: UDCA (100) 2L: UDCA + Bezafibrate (100)	Follow-up: 70.8†
99	Renteria 2024a[y]	Conference abstract	Retrospective observational study	Mexico	132	1L: UDCA (100)	111
100	Ritter 2025	Journal article	Retrospective observational study	USA	2402	1L: UDCA (68.2) 2L: OCA/Fenofibrate (3.6)	165; Follow-up: 26.4†
101	Roberts 2024	Journal article	Prospective observational study	Canada	823	1L: UDCA (100) 2L: OCA (7.3), Bezafibrate (2.6), Fenofibrate (5)	Follow-up for primary cohort: 78†; for validation cohort: 85.2†

S.No.	Study name	Publication Type	Study design	Country	Sample size	Treatment details	Study duration (months)
102	Ronca 2026[ε]	Conference Abstract	Retrospective observational study	Global	866	1L: UDCA (100) 2L: Fibrates/OCA (100)	Follow-up: 37†
103	Shen 2026[δ]	Journal article	Retrospective and prospective observational study	China	240	1L: UDCA (100)	114; Follow-up: 50.0†
104	Shi 2013	Journal article	Prospective observational	China	262	1L: UDCA (100)	192; Follow-up: 75.2†
105	Su 2026	Journal article	Retrospective observational study	China	161	1L: UDCA (100) 2L: UDCA + fenofibrate (100)	170; Follow-up: 42.5†
106	Tababi 2024	Journal article	Retrospective observational study	Tunisia	50	1L: UDCA (100)	Follow-up: 76†
107	Tadros 2024	Conference abstract	Retrospective observational study	USA	160	1L: UDCA (69.4) 2L: OCA (7.5), Statin (25.6)	252

S.No.	Study name	Publication Type	Study design	Country	Sample size	Treatment details	Study duration (months)
108	Takano 2013	Conference abstract	Retrospective observational	Japan	329	1L: UDCA (100) 2L: UDCA + Bezafibrate (32.2)	Follow-up: 240
109	Takano 2020	Journal article	Retrospective observational	Japan	150	1L: UDCA (100) 2L: UDCA + Bezafibrate (100)	268
110	Takeuchi 2023	Conference abstract	Retrospective observational	Japan	799	1L: UDCA (100) 2L: UDCA + Bezafibrate (22.5)	Follow-up: 74.4†
111	Tanaka 2015	Journal article	Retrospective observational	Japan	1121	1L: UDCA (100) 2L: Bezafibrate + UDCA (25.3)	120; Follow-up: 73.2‡
112	Teh 2014	Conference abstract	Prospective observational	Singapore	121	1L: UDCA (NR)	108
113	Tkachuk 2026a[n]	Conference Abstract	Retrospective observational study	Canada	1470	1L: UDCA (96)	204
114	Trivedi 2014a	Journal article	Prospective observational	UK	1015	1L: UDCA (84.5)	Derivation cohort: 198; Follow-up for derivation

S.No.	Study name	Publication Type	Study design	Country	Sample size	Treatment details	Study duration (months)
							cohort: 79†; for validation cohort:87†
115	Trivedi 2014b[r]	Conference abstract	Retrospective observational	Global	3546	1L: UDCA (NR)	Follow-up: 103.2†
116	Van 1999	Journal article	Prospective observational	Netherlands	203	1L: UDCA (100)	70; Follow-up: 47.3†
117	Wang 2024	Journal article	Retrospective observational study	China	117	1L: UDCA (100)	168; Follow-up: 68‡
118	Werner 2025a[z]	Conference abstract	Retrospective observational study	Netherlands	3089	1L: UDCA (100)	408; Follow-up: 99.6†
119	Wong 2025	Conference abstract	Retrospective observational study	Global	1793	NR	Follow-up: 22†
120	Wong 2026	Journal article	Retrospective observational study	Global	1793	1L: UDCA (97.3) 2L: OCA (9.7), Fibrate (17.2)	228; Follow-up: 22†

S.No.	Study name	Publication Type	Study design	Country	Sample size	Treatment details	Study duration (months)
121	Yang 2016	Journal article	Retrospective observational	China	323	1L: UDCA (100) 2L: Fenofibrate, prednisolone, immunosuppressant (NR)	113; Follow-up: 39‡
122	Yang 2023	Journal article	Retrospective observational	China	569	1L: UDCA (100)	216; Follow-up: 59‡
123	Yoo 2018	Journal article	Retrospective observational	South Korea	271	1L: UDCA (100)	204; Follow-up: 120
124	Yoon 2025	Conference Abstract	Retrospective observational study	USA	312	1L: UDCA (100)	192; Follow-up: 48†
125	Zhang 2013	Journal article	Prospective observational	China	187	1L: UDCA (100)	204; Follow-up: 69.6†
126	Zhang 2015[v]	Conference abstract	Retrospective case-control	China	630	1L: UDCA (NR)	144
127	Zhang 2025	Journal article	Retrospective observational study	China	180	1L: UDCA (100)	112; Follow-up: 61.2†

S.No.	Study name	Publication Type	Study design	Country	Sample size	Treatment details	Study duration (months)
128	Zhu 2024	Journal article	Retrospective observational study	China	257	1L: UDCA (100)	96; Follow-up: 60‡
129	Zhu 2025	Journal article	Retrospective observational study	China	232	1L: UDCA (100)	131; Follow-up: 41.8‡

1L: First-line; 2L: Second-line; 3L: Third line; NR: Not reported; OCA: Obeticholic acid; RCT: Randomized controlled trial; UDCA: Ursodeoxycholic acid; UK: United Kingdom; USA: United States of America.
§ Treatment details available only for N = 164 patients. † Median data. ‡ Mean data

Linked publications: [a]Corpechot 2011, [b]Kuiper 2010; Kuiper 2009b; Hansen 2011, [c]Corpechot 2024; Corpechot 2021, [d]Drazilova 2024, [e]Murillo 2021, [f]Fujinaga 2018; Fujinaga 2019b, [g]Azemoto 2011b, [h]Örnolfsson 2018, [i]Lau 2018, [j]De Vincentis 2024b; Terracciani 2024; Antonio 2025; De Vincentis 2024c, [k]Haldar 2018, [l]Gerussi 2020b, [m]Patanwala 2010b, [n]Osman 2020, [o]Perez 2019, [p]Pinyopornpanish 2020, [q]Lammers 2014; Lammers 2015; Lammers 2014; Goet 2021, [r]Trivedi 2014c, [s]Momah 2011, [t]Perez 2017, [u]Montano-Loza 2022, [v]Li 2016, [w]Kowdley 2026, [x]Reddy 2026, [y]Renteria 2024b; Renteria 2024c; Pena-Arellano 2024, [z]Weijsters 2025; Weijsters 2026; Werner 2025b; Werner 2026a; Werner 2026b; van Hooff 2025a; van Hooff 2025b; Werner 2026c, [α]Koky 2025b, [β]Chen 2025, [γ]Trivedi 2025; Abbas 2024, [δ]Shen 2025, [ε]Ronca 2025, [η]Tkachuk 2026b, [θ]Cançado 2025; Couto 2025

Table S4: Patient Characteristics of Included Studies

S.No.	Study name	Mean age, years	Gender	Race			
			Females, n (%)	Black	White	Asian	Other
1	Abbas 2026	45[†]<br>53[$]	302 (89)	-	-	-	-
2	Ampuero 2024	60.7	345 (89.1)	-	-	-	-
3	Arvaniti 2025	50.3 [†]	394 (78)	-	-	-	-
4	Azemoto 2009	55.6	70 (84.3)	-	-	-	-
5	Azemoto 2011a	58.0[†]	114 (85.1)	-	-	134 (100.0)[¶]	-
6	Bonnand 1999	55.3	250 (91.5)	-	-	-	-
7	Carbone 2016	54.9[†]	3527 (90.0)	-	-	-	-
8	Chang 2023	55.2[†]	152 (87.9)	-	-	173 (100.0)	-
9	Chen 2026	53.9[‡]	474 (85.7)	-	-	-	-
10	Cheung 2016	46.1	113 (94.2)	-	-	-	-
11	Copaci 2011	53	57 (92.0)	-	-	-	-
12	Corpechot 2008	53	269 (92.0)	-	-	-	-
13	Corpechot 2022	56.6	3005 (90.8)	-	-	-	-
14	Couto 2026	53[†]	2036 (93.3)	-	-	-	-
15	de Veer 2022	52.3[‡]	3423 (90.9)	-	-	-	-
16	de Vincentis 2024a	58.0[†]	562 (89.0)	-	-	-	-
17	de Tijera 2025	52.5	314 (95.4)	-	-	-	-

S.No.	Study name	Mean age, years	Gender	Race			
			Females, n (%)	Black	White	Asian	Other
18	Ding 2025a	54	381 (90.0)	-	-	-	-
19	Ding 2025b	53	613 (86.0)	-	-	-	-
20	Ding 2025c	57	151 (89.0)	-	-	-	-
21	Fujinaga 2019a	53.5	35 (100.0)	-	-	-	-
22	Fujinaga 2025	59	194 (85.8)	-	-	-	-
23	Gatselis 2026	56.4†	1186 (85.6)	-	-	-	-
24	Gazda 2021	56.0†	234 (94.0)	-	-	-	-
25	Gerussi 2020a	53.0†	1938 (91.0)	-	-	-	-
26	Gish 2025	60.7	592 (81.5)	-	372 (51.2)	-	-
27	Gish 2026	63.5	876 (86.3)	65 (6.4)	542 (53.4)	26 (2.6)	44 (4.3)
28	Haddadin 2019	68.4	61 (89.0)	-	-	-	-
29	Haldar 2021	51.5‡	441 (88.4)	-	-	-	-
30	Harms 2019	54.3	3551 (91.0)	-	-	-	-
31	Hernández 2024a	56.0†	356 (89.0)	-	-	-	-
32	Hernández 2024b	57†	193 (90.0)	-	-	-	-
33	Hofer 2024	63.5†	37 (88.1)	-	-	-	-
34	Huang 2017	47	61 (82.4)	-	-	74 (100.0)§	
35	Huang 2018	46.0†	99 (83.2)	-	-	119 (100.0)§	

S.No.	Study name	Mean age, years	Gender	Race			
			Females, n (%)	Black	White	Asian	Other
36	Huet 2008	50.3	118 (89.4)	-	-	-	-
37	Iwadare 2024	59.0†	159 (88.3)				
38	John 2021a	59.5†	48 (45.7)	6 (5.7)	91 (86.7)	-	8 (7.6)
39	John 2021b	65.0†	114 (21.4)	30 (5.6)	386 (72.6)	-	62 (11.7)
40	Kimura 2021	62.0‡	167(85.2)	-	-	-	-
41	Koky 2025a	57	147 (94.8)	-	-	-	-
42	Kowdley 2024	62	19563 (87.0)	-	-	-	-
43	Kowdley 2025	59.4	3505 (88.2)	218 (5.5)	1964 (49.4)	150 (3.8)	138 (3.5)
44	Kugiyama 2025	62.2	1036 (80.7)	-	-	-	-
45	Kuiper 2009a	54.7	335 (89.0)	-	-	-	-
46	Kumagi 2010a	47.4	65 (94.2)	-	-	-	-
47	Kumagi 2010b	52.5‡	621 (91.0)	-	-	-	-
48	Lam 2023	58.4	2037 (90.8)	-	-	-	-
49	Lammers 2013a	54	1921 (91.0)	-	-	-	-
50	Lammers 2013b	51.5	3544 (91.0)	-	-	-	-
51	Lammers 2016a	55.4	597 (86.9)	-	-	-	-
52	Lammers 2016b	55.5	1675 (92.0)	-	-	-	-
53	Lammers 2018	53.6‡	4317 (89.6)	-	-	-	-

S.No.	Study name	Mean age, years	Gender	Race			
			Females, n (%)	Black	White	Asian	Other
54	Lammert 2014	50.0†	344 (86.5)	-	-	-	-
55	Leeman 2014	54	741 (87.0)	-	-	-	-
56	Li 2025	50.6	52 (82.5)				
57	Lim 2025	57.6	4087 (83.7)	-	-	-	-
58	Lin 2019	53.8	128 (85.3)	-	-	150 (100.0)	-
59	Liu 2016	55.0†	400 (87.9)	-	-	432 (94.9)	23 (5.1)
60	Miura 2025	57.7	180 (79.6)	-	-	-	-
61	Momah 2012	54.5	67 (91.8)	-	-	-	-
62	Montano-Loza 2024	58	206 (89.0)	-	-	-	-
63	Murillo 2018	56.1	1900 (91.3)	-	-	-	-
64	Murillo 2019	52.4	1649 (90.2)	-	-	-	-
65	Murillo 2020a	54.6	2354 (92.1)	-	-	-	-
66	Murillo 2020b	56.1	211 (90.2)	-	-	-	-
67	Nakamura 2015	55.5†	255 (88.8)	-	-	-	-
68	Namisaki 2017	56.0†	90 (90.9)	-	-	-	-
69	Ohfuji 2026	60†	460 (82.0)	-	-	-	-
70	Okumura 2025	57.5†	234 (85.4)	-	-	-	-
71	Örnolfsson 2019	62	182 (82.0)		-	-	-

S.No.	Study name	Mean age, years	Gender	Race			
			Females, n (%)	Black	White	Asian	Other
72	Osman 2021	60.6†	483 (89.8)	-	-	-	-
73	Padilla-Lopez 2026	64 †	110 (87)	-	-	-	-
74	Papastergiou 2013	56.8	207 (96.3)	-	-	-	-
75	Parés 2006	54.1	181 (94.0)	-	-	-	-
76	Pariente 2021	57	385 (88.3)	-	-	-	-
77	Perez 2021	55.2$	510 (89.0)	-	-	-	-
78	Pinyopornpanish 2022	58.5‡	254 (87.0)	14 (4.8)	270 (92.5)	-	-
79	Qian 2020	49.0†	75 (93.75)	-	-	-	-
80	Qu 2019	61.1	58 (89.2)	-	-	-	-
81	Rachel 2026	56.5	1013 (89.1)	-	-	-	-
82	Reddy 2024	63.5†	217 (93.5)	-	-	-	-
83	Reig 2018	53.1†	45 (94.0)	-	-	-	-
84	Reig 2019	53.0†	51 (94.5)	-	-	-	-
85	Renteria 2024a	57.0†	127 (96.3)	-	-	-	-
86	Ritter 2025	63.3	2049 (85.3)	-	-	-	-
87	Roberts 2024	53	749 (91.0)	-	-	-	-
88	Ronca 2026	56.0†	761 (87.9)	-	-	-	-
89	Shen 2026	66.0†	202 (84.15)	-	-	-	-

S.No.	Study name	Mean age, years	Gender	Race			
			Females, n (%)	Black	White	Asian	Other
90	Shi 2013	51.5	240 (91.6)	-	-	-	-
91	Su 2026	52†	131 (81.3)	-	-	-	-
92	Tababi 2024	56.0†	49 (98.0)	-	-	-	-
93	Tadros 2024	55.5	148 (92.5)	8 (5.0)	91 (56.8)	2 (1.2)	
94	Takano 2013	54	278 (84.5)	-	-	329 (100.0)¶	
95	Takano 2020	54	129 (86.0)	-	-	-	-
96	Takeuchi 2023	58.9	679 (85.0)	-	-	799 (100.0)¶	-
97	Tanaka 2015	56.1‡	975 (87.0)	-	-	-	-
98	Teh 2014	63	106 (87.6)	-	-	105 (87.0)§	-
99	Tkachuk 2026a	58†	1,291 (88.0)	-	-	-	-
100	Trivedi 2014a	52.5†	924(91.0)	-	-	-	-
101	Van 1999	56.3	179 (88.2)	-	-	-	-
102	Wang 2024	62	101 (86.3)	-	-	-	-
103	Werner 2025	57.7†	2705 (87.6)	-	-	-	-
104	Wong 2026	51†	1641 (91.5)	-	-	-	-
105	Yang 2016	50	294 (91.0)	-	-	323 (100.0)§	-
106	Yang 2023	50.0†	476 (84.0)	-	-	569 (100.0)§	-
107	Yoo 2018	56.4	236 (87.1)	-	-	271 (100.0)	-

S.No.	Study name	Mean age, years	Gender	Race			
			Females, n (%)	Black	White	Asian	Other
108	Zhang 2013	51	176 (94.0)	-	-	-	-
109	Zhang 2025	55.7	153 (85.0)	-	-	-	-
110	Zhu 2024	56.2	223 (86.8)	-	-	-	-
111	Zhu 2025	58.9	191 (82.3)				

†Median data

‡Age at diagnosis

$Age at treatment initiation

§Chinese patients

¶Japanese patients

Table S5: Newcastle-Ottawa Scale (NOS) for Cohort Studies (n = 126)

Study	S1	S2	S3	S4	C1	C2	O1	O2	O3	Score
Abbas 2026	Yes	Yes	Yes	Yes	Yes	Yes	Yes	Yes	No	**8**
Ampuero 2024	Yes	NA	Yes	Yes	NA	No	Yes	Yes	Yes	**6**
Arvaniti 2025	Yes	Yes	No	No	Yes	No	Yes	Yes	No	**5**
Azemoto 2009	Yes	NA	Yes	Yes	NA	No	Yes	Yes	Yes	**6**
Azemoto 2011a	Yes	NA	Yes	Yes	NA	No	Yes	Yes	Yes	**6**
Carbone 2013	Yes	NA	Yes	Yes	No	No	No	Yes	Yes	**5**
Carbone 2015a	Yes	NA	Yes	Yes	No	No	No	Yes	Yes	**5**
Carbone 2016	Yes	Yes	Yes	Yes	NA	No	Yes	Yes	Yes	**7**
Chang 2023	Yes	NA	Yes	Yes	NA	No	Yes	Yes	Yes	**6**
Chen 2026	Yes	Yes	Yes	Yes	Yes	Yes	Yes	No	No	**7**
Cheung 2016	Yes	NA	Yes	Yes	NA	No	Yes	Yes	Yes	**6**
Cheung 2017	No	NA	Yes	Yes	No	No	No	Yes	Yes	**4**
Copaci 2011	No	NA	Yes	Yes	No	No	No	Yes	Yes	**4**
Corpechot 2008	Yes	NA	Yes	Yes	NA	No	Yes	Yes	Yes	**6**
Corpechot 2016	No	NA	Yes	Yes	No	No	No	Yes	Yes	**4**
Corpechot 2022	Yes	NA	Yes	Yes	NA	No	Yes	Yes	Yes	**6**
Couto 2026	Yes	Yes	Yes	No	Yes	No	Yes	Yes	No	**6**
de Tijera 2025	Yes	Yes	Yes	No	Yes	Yes	Yes	No	No	**6**
de Veer 2022	Yes	NA	Yes	Yes	NA	No	Yes	Yes	Yes	**6**
De Vincentis 2024a	Yes	NA	Yes	Yes	No	No	No	Yes	Yes	**5**
Ding 2025a	Yes	No	Yes	Yes	No	No	Yes	Yes	Yes	**6**
Ding 2025b	Yes	No	Yes	Yes	No	No	Yes	Yes	Yes	**6**
Ding 2025c	Yes	Yes	Yes	Yes	Yes	Yes	Yes	No	No	**7**
Dorizas 2025	Yes	Yes	Yes	Yes	Yes	No	Yes	No	No	**6**
Fujinaga 2017	No	NA	Yes	Yes	No	No	No	Yes	Yes	**4**
Fujinaga 2019a	Yes	NA	Yes	Yes	NA	No	Yes	Yes	Yes	**6**
Fujinaga 2025	Yes	No	Yes	Yes	No	No	Yes	Yes	Yes	**6**
Gatselis 2026	Yes	Yes	Yes	Yes	Yes	No	Yes	No	No	**6**

Study	S1	S2	S3	S4	C1	C2	O1	O2	O3	Score
Gazda 2021	Yes	NA	Yes	Yes	NA	No	Yes	Yes	Yes	**6**
Gerussi 2020a	Yes	NA	Yes	Yes	No	No	No	Yes	Yes	**5**
Gish 2025	Yes	Yes	Yes	Yes	Yes	No	Yes	No	No	**6**
Gish 2026	Yes	Yes	Yes	Yes	Yes	No	Yes	No	No	**6**
Haddadin 2019	Yes	NA	Yes	Yes	No	No	No	Yes	Yes	**5**
Haldar 2021	Yes	NA	Yes	Yes	NA	No	Yes	Yes	Yes	**6**
Harms 2015	No	NA	Yes	Yes	No	No	No	Yes	Yes	**4**
Harms 2019	Yes	NA	Yes	Yes	NA	No	Yes	Yes	Yes	**6**
Hayashi 2024	Yes	No	Yes	Yes	No	No	Yes	Yes	Yes	**6**
Hernández 2024a	Yes	No	Yes	Yes	No	No	Yes	NA	NA	**4**
Hernández 2024b	Yes	Yes	Yes	Yes	Yes	Yes	Yes	No	No	**7**
Hofer 2024	Yes	NA	Yes	Yes	NA	No	Yes	Yes	Yes	**6**
Huang 2017	Yes	NA	Yes	Yes	No	No	No	Yes	Yes	**5**
Huang 2018	Yes	NA	Yes	Yes	No	No	No	Yes	Yes	**5**
Huet 2008	Yes	NA	Yes	Yes	NA	No	Yes	Yes	Yes	**6**
Iwadare 2024	Yes	No	Yes	Yes	No	No	Yes	Yes	Yes	**6**
John 2021a	Yes	NA	Yes	Yes	NA	No	Yes	Yes	Yes	**6**
John 2021b	Yes	NA	Yes	Yes	NA	No	Yes	Yes	Yes	**6**
Kimura 2021	Yes	NA	Yes	Yes	NA	No	Yes	Yes	Yes	**6**
Koky 2025a	Yes	No	Yes	Yes	No	No	Yes	Yes	Yes	**6**
Kowdley 2024	Yes	Yes	Yes	Yes	Yes	No	Yes	NA	NA	**6**
Kowdley 2025	Yes	No	Yes	Yes	No	No	Yes	Yes	Yes	**6**
Kugiyama 2025	Yes	No	Yes	Yes	No	No	Yes	NA	NA	**4**
Kuiper 2009a	Yes	NA	Yes	Yes	NA	No	Yes	Yes	Yes	**6**
Kumagi 2010a	Yes	NA	Yes	Yes	NA	No	Yes	Yes	Yes	**6**
Kumagi 2010b	No	NA	Yes	Yes	No	No	No	Yes	Yes	**4**
Lam 2023	Yes	NA	Yes	Yes	No	No	No	Yes	Yes	**5**
Lammers 2013a	No	NA	Yes	Yes	No	No	No	Yes	Yes	**4**
Lammers 2013b	Yes	NA	Yes	Yes	No	No	No	Yes	Yes	**5**

Study	S1	S2	S3	S4	C1	C2	O1	O2	O3	Score
Lammers 2016a	Yes	NA	Yes	Yes	NA	No	Yes	Yes	Yes	**6**
Lammers 2016b	Yes	NA	Yes	Yes	No	No	No	Yes	Yes	**5**
Lammers 2018	No	NA	Yes	Yes	No	No	No	Yes	Yes	**4**
Lammert 2014	Yes	NA	Yes	Yes	NA	No	Yes	Yes	Yes	**6**
Leeman 2014	Yes	NA	Yes	Yes	No	No	No	Yes	Yes	**5**
Li 2025	Yes	No	Yes	Yes	No	No	Yes	Yes	Yes	**6**
Lim 2025	Yes	Yes	Yes	Yes	Yes	Yes	Yes	Yes	Yes	**9**
Lin 2019	Yes	NA	Yes	Yes	NA	No	Yes	Yes	Yes	**6**
Liu 2016	Yes	Yes	Yes	Yes	Yes	No	Yes	Yes	Yes	**8**
Meloni 2024	Yes	No	Yes	Yes	No	No	Yes	NA	NA	**4**
Miura 2025	Yes	No	Yes	Yes	No	No	Yes	Yes	Yes	**6**
Momah 2012	Yes	NA	Yes	Yes	NA	No	Yes	Yes	Yes	**6**
Montano-Loza 2024	Yes	No	Yes	Yes	No	No	Yes	Yes	Yes	**6**
Murillo 2018	Yes	NA	Yes	Yes	No	No	No	Yes	Yes	**5**
Murillo 2019	Yes	NA	Yes	Yes	NA	No	Yes	Yes	Yes	**6**
Murillo 2020a	Yes	NA	Yes	Yes	NA	No	Yes	Yes	Yes	**6**
Murillo 2020b	Yes	NA	Yes	Yes	No	No	No	Yes	Yes	**5**
Nakamura 2015	Yes	No	Yes	Yes	No	No	Yes	Yes	Yes	**6**
Namisaki 2017	Yes	NA	Yes	Yes	NA	No	Yes	Yes	Yes	**6**
Ohfuji 2026	Yes	Yes	Yes	Yes	Yes	Yes	Yes	No	No	**7**
Okumura 2025	Yes	Yes	Yes	Yes	Yes	Yes	Yes	Yes	No	**8**
Örnolfsson 2019	Yes	NA	Yes	Yes	NA	No	Yes	Yes	Yes	**6**
Osman 2021	Yes	NA	Yes	Yes	NA	No	Yes	Yes	Yes	**6**
Padilla-Lopez 2026	Yes	No	Yes	No	No	No	Yes	No	No	**3**
Papastergiou 2013	Yes	NA	Yes	Yes	NA	No	Yes	Yes	Yes	**6**
Parés 2006	Yes	No	Yes	Yes	No	No	Yes	Yes	Yes	**6**
Pariente 2021	Yes	NA	Yes	Yes	NA	No	Yes	Yes	Yes	**6**
Patanwala 2010a	No	NA	Yes	Yes	No	No	No	Yes	Yes	**4**

Study	S1	S2	S3	S4	C1	C2	O1	O2	O3	Score
Perez 2018	NA	NA	NA	NA	NA	No	NA	NA	NA	**0**
Perez 2021	Yes	Yes	Yes	Yes	Yes	Yes	Yes	No	No	**7**
Pinyopornpanish 2022	Yes	NA	Yes	Yes	NA	No	Yes	Yes	Yes	**6**
Qian 2020	Yes	NA	Yes	Yes	NA	No	Yes	Yes	Yes	**6**
Qu 2019	Yes	No	Yes	Yes	No	No	Yes	Yes	Yes	**6**
Rachel 2026	Yes	Yes	Yes	Yes	Yes	No	Yes	Yes	No	**7**
Reddy 2024	Yes	Yes	Yes	Yes	Yes	Yes	Yes	Yes	Yes	**9**
Reiberger 2019	Yes	NA	Yes	Yes	No	No	No	Yes	Yes	**5**
Reig 2018	Yes	NA	Yes	Yes	NA	No	Yes	Yes	Yes	**6**
Reig 2019	No	NA	Yes	Yes	No	No	No	Yes	Yes	**4**
Renteria 2024a	Yes	Yes	Yes	Yes	Yes	No	Yes	NA	NA	**6**
Ritter 2025	Yes	No	Yes	Yes	Yes	Yes	Yes	Yes	Yes	**8**
Roberts 2024	Yes	No	Yes	Yes	No	No	Yes	Yes	Yes	**6**
Ronca 2026	Yes	Yes	Yes	Yes	Yes	No	Yes	Yes	No	**7**
Shen 2026	Yes	Yes	Yes	Yes	Yes	Yes	Yes	Yes	No	**8**
Shi 2013	Yes	No	Yes	Yes	No	No	Yes	Yes	Yes	**6**
Su 2026	Yes	Yes	Yes	Yes	Yes	Yes	Yes	No	No	**7**
Tababi 2024	Yes	No	Yes	Yes	No	No	Yes	Yes	Yes	**6**
Tadros 2024	Yes	Yes	Yes	Yes	Yes	No	Yes	NA	NA	**6**
Takano 2013	No	NA	Yes	Yes	No	No	No	Yes	Yes	**4**
Takano 2020	Yes	NA	Yes	Yes	NA	No	Yes	Yes	Yes	**6**
Takeuchi 2023	Yes	NA	Yes	Yes	NA	No	Yes	Yes	Yes	**6**
Tanaka 2015	Yes	NA	Yes	Yes	NA	No	Yes	Yes	Yes	**6**
Teh 2014	Yes	NA	Yes	Yes	No	No	No	Yes	Yes	**5**
Tkachuk 2026a	Yes	Yes	Yes	No	Yes	No	Yes	Yes	No	**6**
Trivedi 2014a	Yes	Yes	Yes	Yes	Yes	No	Yes	Yes	Yes	**8**
Trivedi 2014b	Yes	NA	Yes	Yes	No	No	No	Yes	Yes	**5**
Van 1999	Yes	NA	Yes	Yes	NA	No	Yes	Yes	Yes	**6**
Wang 2024	Yes	No	Yes	Yes	No	No	Yes	Yes	Yes	**6**

Study	S1	S2	S3	S4	C1	C2	O1	O2	O3	Score
Werner 2025	Yes	No	Yes	Yes	No	No	Yes	Yes	Yes	**6**
Wong 2025	Yes	No	Yes	Yes	No	No	Yes	Yes	Yes	**6**
Wong 2026	Yes	Yes	Yes	Yes	Yes	Yes	Yes	Yes	Yes	**9**
Yang 2016	Yes	NA	Yes	Yes	NA	No	Yes	Yes	Yes	**6**
Yang 2023	Yes	NA	Yes	Yes	NA	No	Yes	Yes	Yes	**6**
Yoo 2018	Yes	NA	Yes	Yes	NA	No	Yes	Yes	Yes	**6**
Yoon 2025	Yes	Yes	Yes	Yes	Yes	No	Yes	No	No	**6**
Zhang 2013	Yes	No	Yes	Yes	NA	No	Yes	Yes	Yes	**6**
Zhang 2015	Yes	Yes	Yes	Yes	No	No	No	Yes	Yes	**6**
Zhang 2025	Yes	Yes	Yes	Yes	Yes	No	Yes	Yes	Yes	**8**
Zhu 2024	Yes	No	Yes	Yes	No	No	Yes	Yes	Yes	**6**
Zhu 2025	Yes	No	Yes	Yes	No	No	Yes	NA	NA	**4**

S1: Representativeness of the exposed cohort; S2: Selection of the non-exposed cohort; S3: Ascertainment of exposure; S4: Demonstration that the outcome was not present at baseline; C1: Comparability – main factor; C2: Comparability – additional factors; O1: Assessment of outcome; O2: Was follow-up long enough; O3: Adequacy of follow-up of cohorts. "Yes" = star awarded (criterion met); "No" = star not awarded; "NA" = not applicable / not assessed. Maximum score = 9.

Table S6: Cochrane Risk-of-Bias Tool (RoB 2.0) – Randomized Controlled Trials (n = 3)

Study	D1. Randomization process	D2. Deviations from intended interventions	D3. Missing outcome data	D4. Measurement of the outcome	D5. Selection of the reported result	Overall judgement
Bonnand 1999	Some concerns	Some concerns	Low risk	Low risk	Low risk	Some concerns
Angulo 1999	Some concerns	Some concerns	Low risk	Low risk	Low risk	Some concerns
Jorgensen 1995	Some concerns	Some concerns	Low risk	Low risk	Low risk	Some concerns

Figure S2: Risk-of-Bias Assessment of Included Cohort Studies Using the Newcastle-Ottawa Scale (NOS)

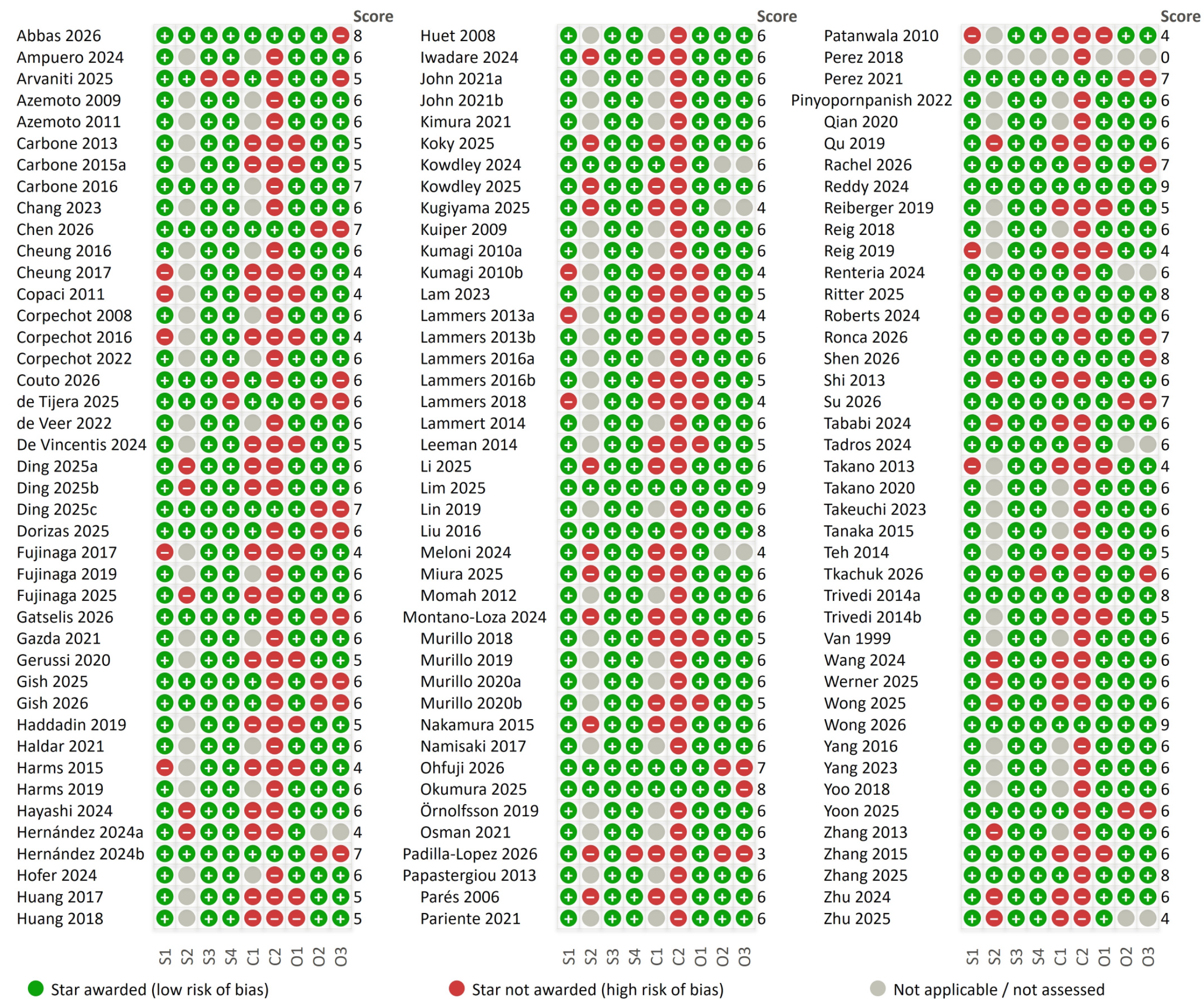

S1: Representativeness of exposed cohort; S2: Selection of non-exposed cohort; S3: Ascertainment of exposure; S4: Outcome not present at baseline; C1: Comparability - main factor; C2: Comparability - additional factors; O1: Assessment of outcome; O2: Follow-up long enough; O3: Adequacy of follow-up

Figure S3: Risk-of-Bias Assessment of Included Randomized Controlled Trials Using the Cochrane Risk-of-Bias Tool for Randomized Trials, Version 2 (RoB 2.0)

	D1. Randomization process	D2. Deviations from intended interventions	D3. Missing outcome data	D4. Measurement of the outcome	D5. Selection of the reported result	Overall
Bonnand 1999	?	?	+	+	+	?
Angulo 1999	?	?	+	+	+	?
Jorgensen 1995	?	?	+	+	+	?

Low risk   Some concerns   High risk

Table S7: Association of Failure to Achieve Response with Secondary Outcomes

Study name	Long-term clinical outcome	Response definition	Sample size	Effect estimate reporting	Effect estimates, HR/OR (95% CI) Non-responders vs responders
**ALP normalization**					
Montano-Loza 2024	Graft loss after LT	ALP normalization	284	HR	1.59 (1.26-2.01)
Montano-Loza 2024	Overall mortality after LT	ALP normalization	284	HR	1.52 (1.29-1.78)
**ALP response**					
Shen 2026	Recompensation	Barcelona	240	HR	0.46 (0.12-1.70)
Montano-Loza 2024	Graft loss after LT	Toronto I	284	HR	2.85 (1.11-7.32)
Montano-Loza 2024	Overall mortality after LT	Toronto I	284	HR	3.28 (1.86-5.77)
Montano-Loza 2024	Graft loss after LT	Rochester II	284	HR	2.79 (1.09-7.15)
Montano-Loza 2024	Overall mortality after LT	Rochester II	284	HR	3.47 (1.97-6.10)
**Composite response**					
Hofer 2024	Recompensation	Paris II	33	HR	0.20 (0.04-0.93)
Hofer 2024	Recompensation	Paris II	33	OR	0.15 (0.02-0.94)
Shen 2026	Recompensation	Paris II	240	HR	0.05 (0.01-0.37)
Shen 2026	Recompensation	Paris I	240	HR	0.06 (0.01-0.49)

Study name	Long-term clinical outcome	Response definition	Sample size	Effect estimate reporting	Effect estimates, HR/OR (95% CI) Non-responders vs responders
Shen 2026	Recompensation	Momah/Lindor	240	HR	0.05 (0.01-0.40)
Ding 2025	Recompensation	Rotterdam	170	HR	0.41 (0.23-0.74)
Montano-Loza 2024	Graft loss after LT	Paris II	284	HR	4.14 (1.57-10.92)
Montano-Loza 2024	Overall mortality after LT	Paris II	284	HR	3.35 (1.90-5.92)
**Other surrogate markers**					
Fujinaga 2025	Liver-related symptoms	Nara criteria	226	HR	1.17 (0.94-1.46)
Hofer 2024	Recompensation	Bilirubin (> 1.2 mg/dL)	42	HR	0.19 (0.04-0.87)
Azemoto 2009	Liver-related symptoms	Ehime	83	OR	5.33 (1.40-20.22)
Kumagi 2010	Disease progression	Bilirubin (> 13.5 μmol/L)	69	OR	5.56 (1.25-25.00)
Fujinaga 2017	Disease improvement	Nara criteria	35	OR	0.65 (0.04-11.33)
Fujinaga 2017	Disease progression	Nara criteria	35	OR	5.45 (0.97-30.60)
Chen 2026	Hepatic events†	Bilirubin normalization	453	HR	5.13 (3.21-8.19)
Montano-Loza 2024	Graft loss after LT	Bilirubin normalization	284	HR	1.40 (1.20-1.63)
Montano-Loza 2024	Overall mortality after LT	Bilirubin normalization	284	HR	1.14 (1.04-1.25)

CI: Confidence Interval; HR: Hazard Ratio; OR: Odds Ratio

†Ascites, variceal bleeding, encephalopathy, or HCC

Figure S4: Association Between Failure to Achieve ALP Response and Adverse Long-Term Clinical Outcomes Using Fixed-Effect Model (A) HR Estimates (B) OR Estimates

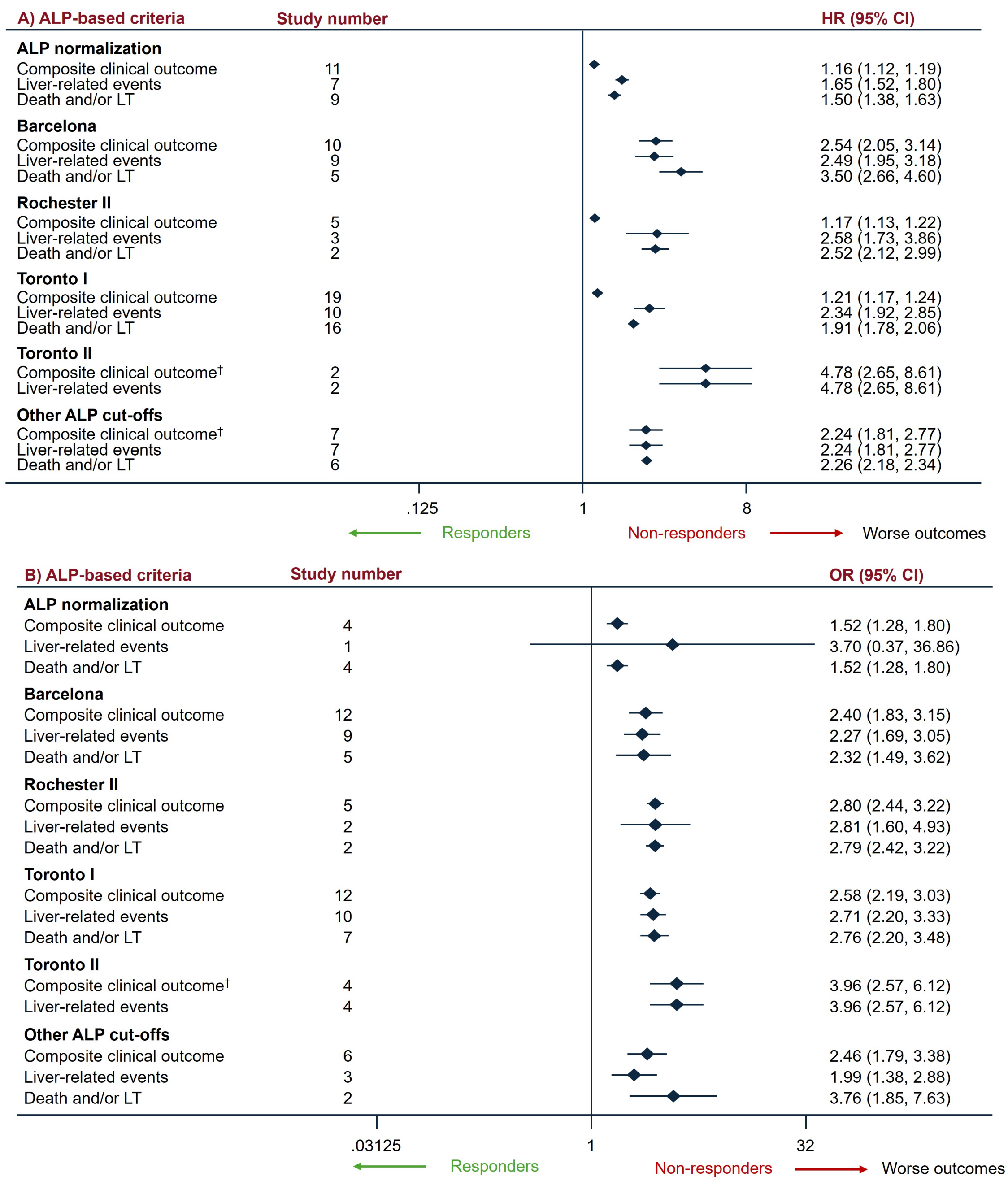

Fixed-effects IV model

Note: Higher HR/OR values represent increased risk of adverse events among non-responders

ALP: Alkaline Phosphatase; CI: Confidence Interval; HR: Hazard Ratio; IV: Inverse-variance; LT: Liver Transplantation; OR: Odds Ratio

†Same studies are contributing to the meta-analysis of composite clinical outcomes and liver-related events

Figure S5: Association Between Failure to Achieve Composite Response and Adverse Long-Term Clinical Outcomes Using Fixed-Effect Model (A) HR Estimates (B) OR Estimates

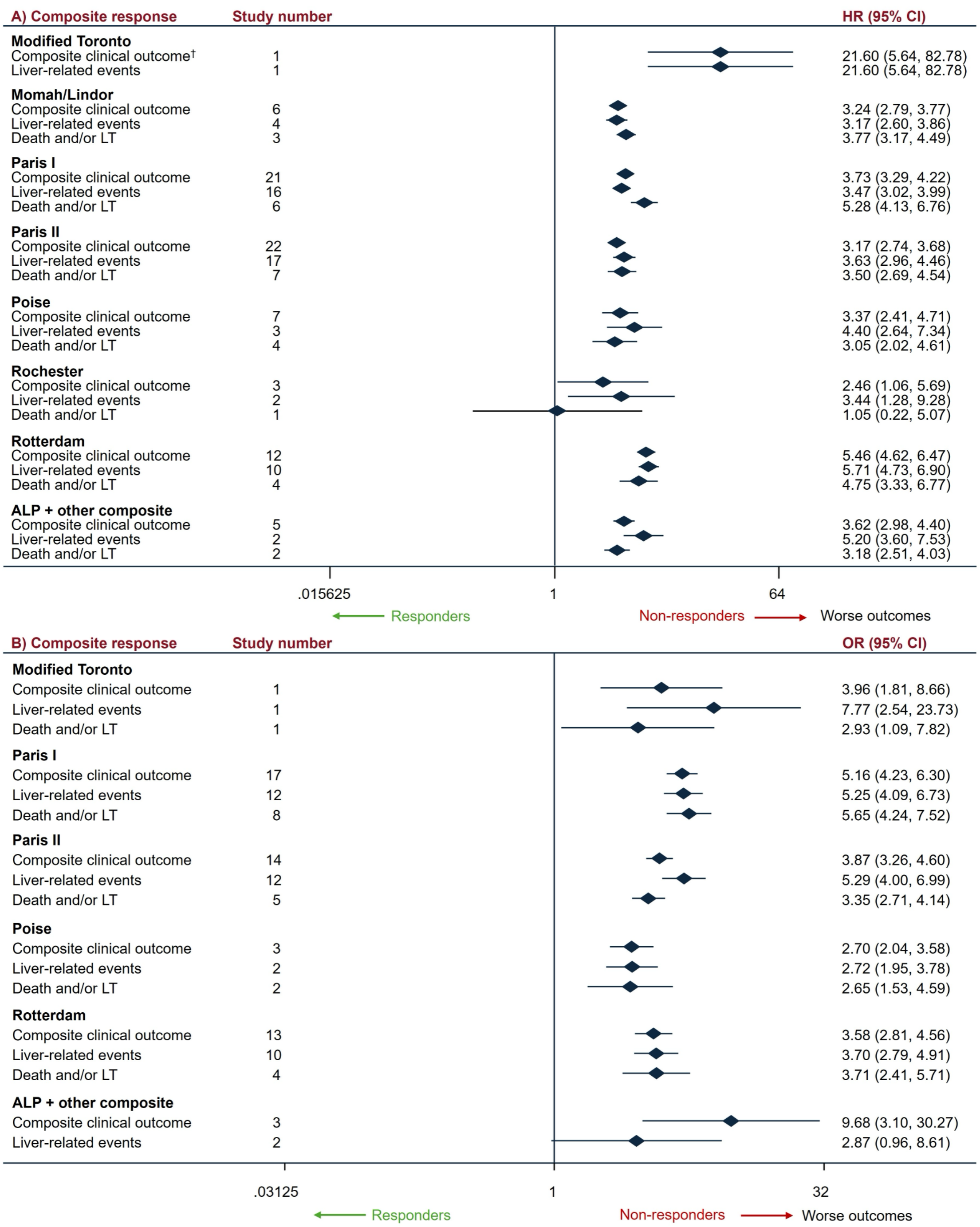

Fixed-effects IV model

Note: Higher HR/OR values represent increased risk of adverse events among non-responders

CI: Confidence Interval; HR: Hazard Ratio; IV: Inverse-variance; LT: Liver Transplantation; OR: Odds Ratio

[†]Same studies are contributing to the meta-analysis of composite clinical outcomes and liver-related events

Figure S6: Association Between Failure to Achieve Response (based on GGT/Ehime/Nara/Bilirubin or Other Criteria) and Adverse Long-Term Clinical Outcomes Using Fixed-Effect Model (A) HR Estimates (B) OR Estimates

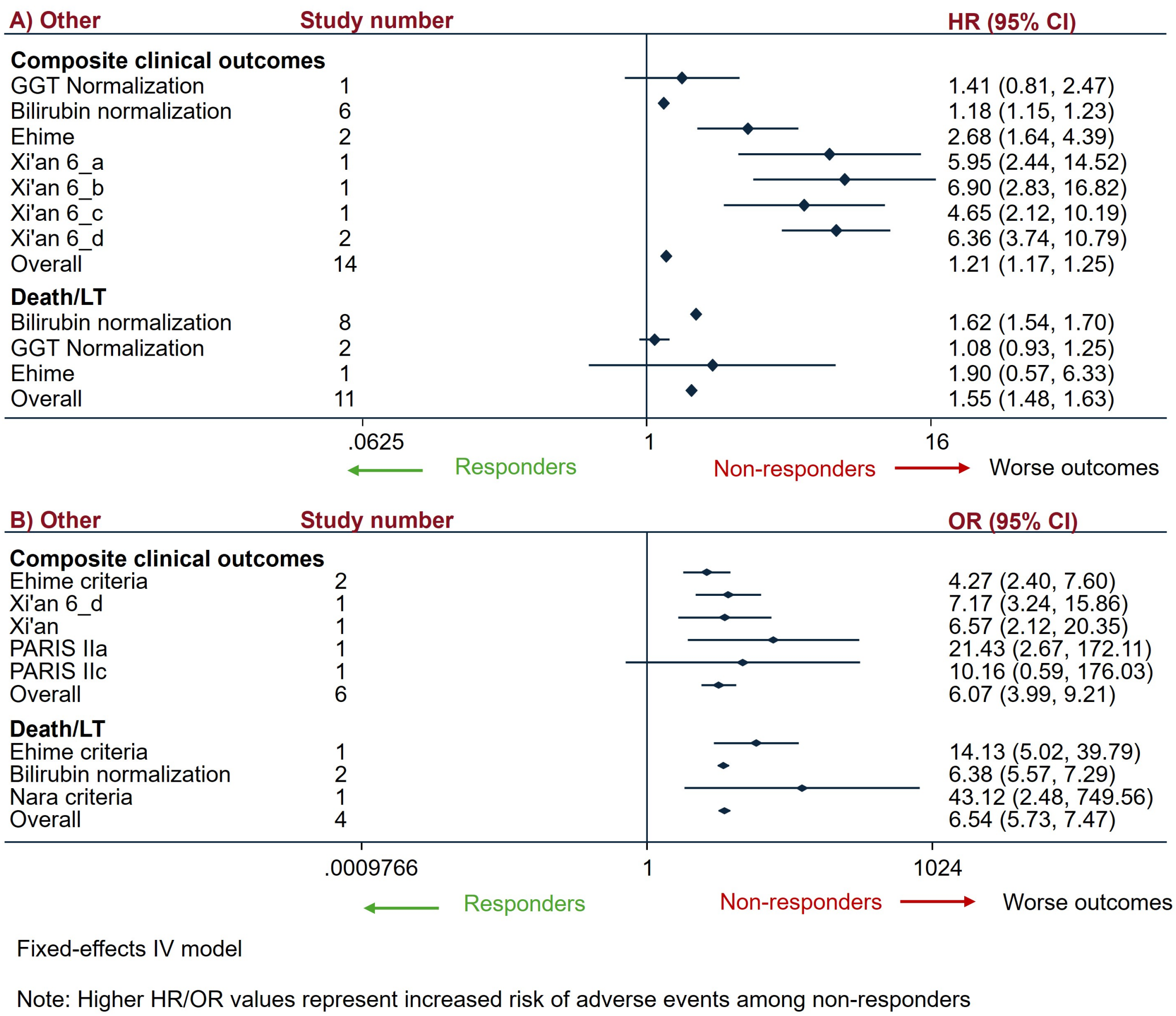

CI: Confidence Interval; GGT: Gamma-Glutamyl Transferase; HR: Hazard Ratio; IV: Inverse-variance; LT: Liver Transplantation; OR: Odds Ratio

Figure S7: Association Between Failure to Achieve Response (based on GGT/Ehime/Nara/Bilirubin or Other Criteria) and Adverse Long-Term Clinical Outcomes in Relation to Liver-Related Death Using Random-Effects Model (A) HR Estimates (B) OR Estimates

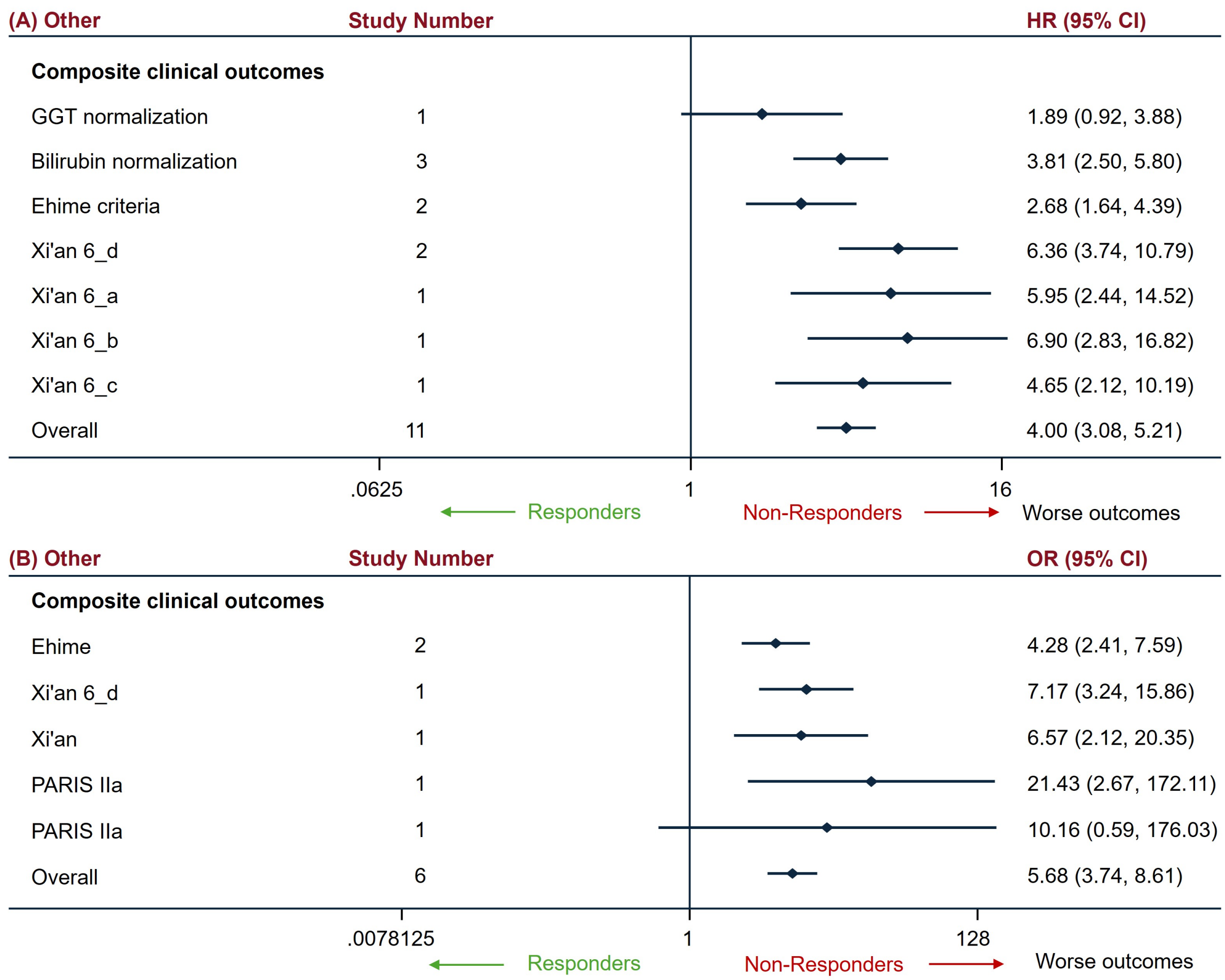

Random-effects DL model

Note: Higher HR/OR values represent increased risk of adverse events among non-responders

CI: Confidence Interval; DL: DerSimonian and Laird; GGT: Gamma-Glutamyl Transferase; HR: Hazard Ratio; LT: Liver Transplantation; OR: Odds Ratio

Figure S8: Association Between Failure to Achieve Response (based on GGT/Ehime/Nara/Bilirubin or Other Criteria) and Adverse Long-Term Clinical Outcomes in Relation to Liver-Related Death Using Fixed-Effect Model (A) HR Estimates (B) OR Estimates

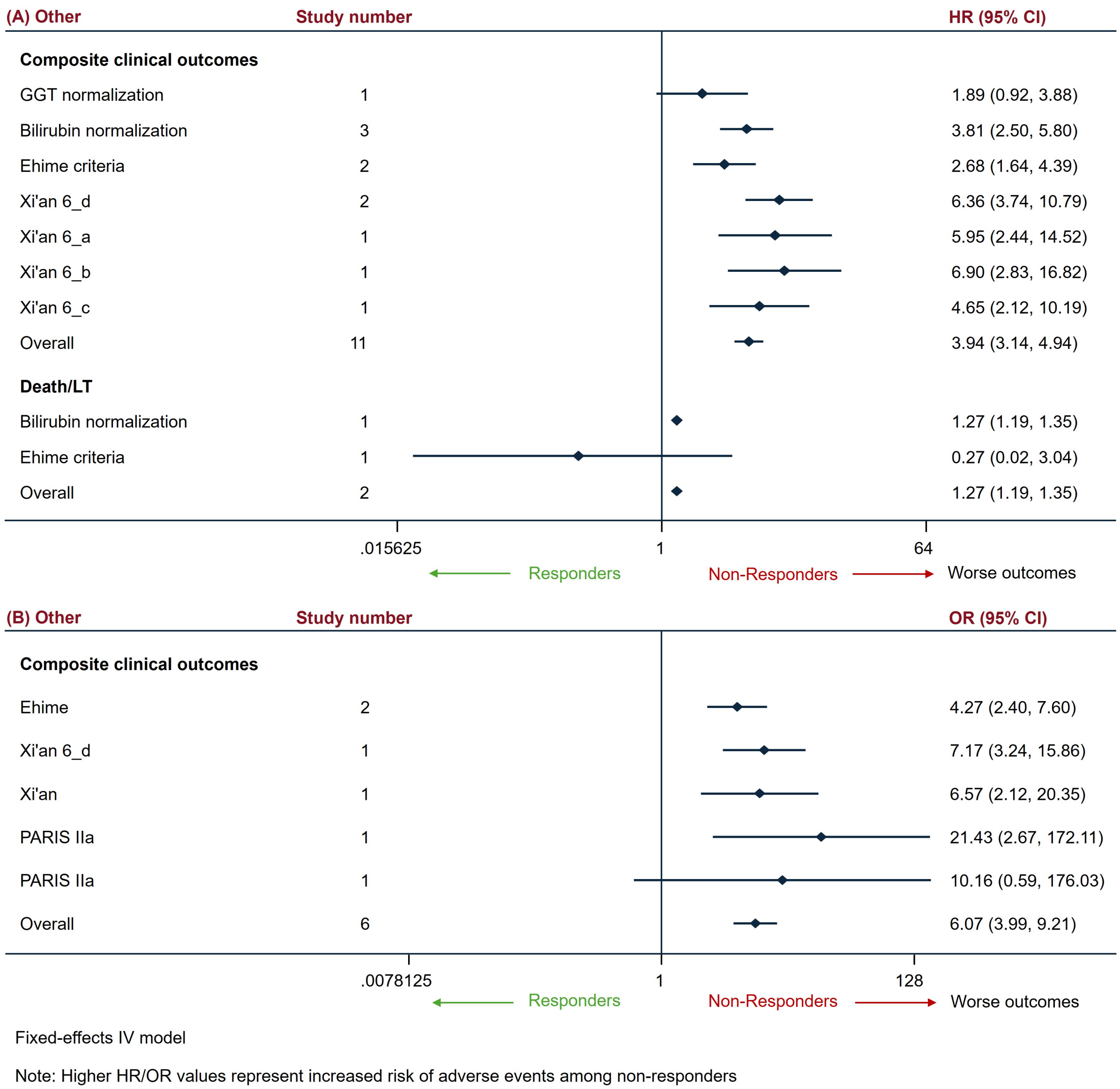

CI: Confidence Interval; GGT: Gamma-Glutamyl Transferase; HR: Hazard Ratio; IV: Inverse-variance; LT: Liver Transplantation; OR: Odds Ratio

Table S8: Base-Case Results Using Random-Effects DL Model

Response	Outcomes	Number of studies	Estimate	LCI	UCI	I^2	p-value†	CocQ	df
**ALP response (HR estimates)**									
ALP normalization	Composite clinical outcome	11	1.46	1.26	1.69	88.7	<0.001	88.86	10
ALP normalization	Liver-related events	7	1.82	1.41	2.34	79.5	<0.001	29.32	6
ALP normalization	Overall death and/or LT	9	1.52	1.3	1.79	62.7	0.006	21.47	8
Barcelona	Composite clinical outcome	10	1.84	1.1	3.06	80.2	<0.001	45.38	9
Barcelona	Liver-related events	9	2.09	1.31	3.34	68.1	0.001	25.11	8
Barcelona	Overall death and/or LT	5	2.35	1.09	5.04	85.1	<0.001	26.76	4
Rochester II	Composite clinical outcome	5	2.22	1.28	3.84	95.8	<0.001	94.88	4
Rochester II	Liver-related events	3	2.58	1.73	3.86	0	0.856	0.31	2
Rochester II	Overall death and/or LT	2	2.52	2.12	2.99	0	0.516	0.42	1
Toronto I	Composite clinical outcome	19	1.87	1.5	2.34	93.1	<0.001	259.27	18
Toronto I	Liver-related events	10	2.56	1.7	3.85	69.8	<0.001	29.78	9
Toronto I	Overall death and/or LT	16	2	1.64	2.44	81	<0.001	78.82	15
Toronto II	Composite clinical outcome	2	4.8	1.33	17.28	78.8	0.03	4.73	1
Toronto II	Liver-related events	2	4.8	1.33	17.28	78.8	0.03	4.73	1
Other ALP cut-offs	Composite clinical outcome	7	2.38	1.75	3.23	37.3	0.144	9.57	6
Other ALP cut-offs	Liver-related events	7	2.38	1.75	3.23	37.3	0.144	9.57	6

Response	Outcomes	Number of studies	Estimate	LCI	UCI	I^2	p-value†	CocQ	df
Other ALP cut-offs	Overall death and/or LT	6	2.26	2.18	2.34	0	0.552	3.98	5
**ALP response (OR estimates)**									
ALP normalization	Composite clinical outcome	4	1.62	1.13	2.31	56.6	0.075	6.92	3
ALP normalization	Liver-related events	1	3.7	0.37	36.86	0	<0.001	0	0
ALP normalization	Overall death and/or LT	4	1.6	1.16	2.22	50.8	0.107	6.1	3
Barcelona	Composite clinical outcome	12	2.52	1.53	4.14	65.5	<0.001	31.87	11
Barcelona	Liver-related events	9	2.36	1.28	4.34	72.1	<0.001	28.67	8
Barcelona	Overall death and/or LT	5	2.55	1.42	4.57	26.8	0.243	5.46	4
Rochester II	Composite clinical outcome	5	3.37	2.24	5.08	45	0.122	7.28	4
Rochester II	Liver-related events	2	2.81	1.6	4.93	0	0.895	0.02	1
Rochester II	Overall death and/or LT	2	4.73	1.35	16.62	85.6	0.009	6.92	1
Toronto I	Composite clinical outcome	12	3.1	2.23	4.3	64.4	0.001	30.9	11
Toronto I	Liver-related events	10	3.04	2	4.61	66.9	0.001	27.19	9
Toronto I	Overall death and/or LT	7	3.54	2.24	5.6	63.4	0.012	16.4	6
Toronto II	Composite clinical outcome	4	4.22	1.93	9.23	59	0.063	7.31	3
Toronto II	Liver-related events	4	4.22	1.93	9.23	59	0.063	7.31	3
Other ALP cut-offs	Composite clinical outcome	6	2.93	1.8	4.76	47	0.093	9.43	5
Other ALP cut-offs	Liver-related events	3	2.05	1.33	3.19	22.2	0.277	2.57	2

Response	Outcomes	Number of studies	Estimate	LCI	UCI	I^2	p-value†	CocQ	df
Other ALP cut-offs	Overall death and/or LT	2	3.76	1.85	7.63	0	0.725	0.12	1
**Composite response (HR estimates)**									
Modified Toronto	Composite clinical outcome	1	21.6	5.64	82.78	0	<0.001	0	0
Modified Toronto	Liver-related events	1	21.6	5.64	82.78	0	<0.001	0	0
Momah/Lindor	Composite clinical outcome	6	2.66	1.62	4.37	88.6	<0.001	43.76	5
Momah/Lindor	Liver-related events	4	2.98	1.61	5.5	85.5	<0.001	20.66	3
Momah/Lindor	Overall death and/or LT	3	2.77	1.42	5.42	92	<0.001	25.09	2
Paris I	Composite clinical outcome	21	4.11	3.08	5.48	78.8	<0.001	94.13	20
Paris I	Liver-related events	16	4.04	2.79	5.84	82.9	<0.001	87.62	15
Paris I	Overall death and/or LT	6	5.24	4.02	6.82	9.6	0.354	5.53	5
Paris II	Composite clinical outcome	22	3.71	2.92	4.72	54.4	0.001	46.09	21
Paris II	Liver-related events	17	4.5	3.16	6.41	59.7	<0.001	39.68	16
Paris II	Overall death and/or LT	7	3.5	2.69	4.54	0	0.871	2.48	6
Poise	Composite clinical outcome	7	3.37	2.41	4.71	0	0.897	2.23	6
Poise	Liver-related events	3	4.4	2.64	7.34	0	0.997	0.01	2
Poise	Overall death and/or LT	4	3.05	2.02	4.61	0	0.942	0.39	3
Rochester	Composite clinical outcome	3	1.78	0.37	8.55	68.1	0.043	6.28	2
Rochester	Liver-related events	2	2.13	0.18	24.71	78.8	0.03	4.72	1

Response	Outcomes	Number of studies	Estimate	LCI	UCI	I^2	p-value†	CocQ	df
Rochester	Overall death and/or LT	1	1.05	0.22	5.07	0	<0.001	0	0
Rotterdam	Composite clinical outcome	12	4.48	3.14	6.37	72.2	<0.001	39.63	11
Rotterdam	Liver-related events	10	4.37	2.79	6.86	76.8	<0.001	38.73	9
Rotterdam	Overall death and/or LT	4	4.75	3.33	6.77	0	0.922	0.48	3
ALP + Other composite	Composite clinical outcome	5	3.66	2.8	4.78	35.4	0.185	6.19	4
ALP + Other composite	Liver-related events	2	5.2	3.6	7.53	0	0.349	0.88	1
ALP + Other composite	Overall death and/or LT	2	3.18	2.51	4.03	0	0.776	0.08	1
**Composite response (OR estimates)**									
Modified Toronto	Composite clinical outcome	1	3.96	1.81	8.66	0	<0.001	0	0
Modified Toronto	Liver-related events	1	7.77	2.54	23.73	0	<0.001	0	0
Modified Toronto	Overall death and/or LT	1	2.93	1.09	7.82	0	<0.001	0	0
Paris I	Composite clinical outcome	17	5.23	4.22	6.48	8.4	0.356	17.47	16
Paris I	Liver-related events	12	5.31	4.00	7.04	16.6	0.281	13.19	11
Paris I	Overall death and/or LT	8	5.65	4.24	7.52	0	0.454	6.76	7
Paris II	Composite clinical outcome	14	5.18	3.4	7.89	66.4	<0.001	38.7	13
Paris II	Liver-related events	12	5.3	3.33	8.44	58	0.006	26.2	11
Paris II	Overall death and/or LT	5	6.9	2.67	17.85	56.5	0.056	9.19	4
Poise	Composite clinical outcome	3	2.7	2.04	3.58	0	0.448	1.61	2

Response	Outcomes	Number of studies	Estimate	LCI	UCI	I^2	p-value†	CocQ	df
Poise	Liver-related events	2	3.2	1.56	6.57	53.9	0.141	2.17	1
Poise	Overall death and/or LT	2	2.65	1.53	4.59	0	0.816	0.05	1
Rotterdam	Composite clinical outcome	13	3.89	2.46	6.15	65.5	<0.001	34.83	12
Rotterdam	Liver-related events	10	3.77	2.14	6.67	69.9	<0.001	29.91	9
Rotterdam	Overall death and/or LT	4	5.08	2.32	11.09	54.7	0.085	6.62	3
Other Composite Response	Composite clinical outcome	3	9.68	3.1	30.27	0	0.627	0.93	2
Other Composite Response	Liver-related events	2	3.16	0.82	12.22	23.7	0.252	1.31	1

ALP: Alkaline Phosphatase; CocQ: Cochran's Q; df: Degrees of freedom; DL: DerSimonian and Laird; HR: Hazard ratio; LCI: Lower confidence interval; LT: Liver transplantation; OR: Odds ratio; τ^2: Tau-square; UCI: Upper confidence interval

†p-values corresponding to I^2

For ALP response analyses, τ^2 values for composite clinical outcome, liver-related events, and overall death and/or LT were 0.056267, 0.137020, and 0.078815, respectively, for HR estimates, and 0.177862, 0.244718, and 0.159368, respectively, for OR estimates. For composite response analyses, the corresponding τ^2 values were 0.232641, 0.327592, and 0.06677084 for HR estimates, and 0.187721, 0.260306, and 0.113778 for OR estimates. τ^2 values relate to the overall pooled analyses within each outcome and effect-measure category

Table S9: Sensitivity analysis Results Using Fixed-Effect IV Model

ALP Response (HR Estimates)									
**Response**	**Outcomes**	**Number of studies**	**HR**	**LCI**	**UCI**	**I^2**	**p-value†**	**CocQ**	**df**
ALP normalization	Composite clinical outcome	11	1.16	1.12	1.19	88.7	<0.001	88.86	10
ALP normalization	Liver-related events	7	1.65	1.52	1.8	79.5	<0.001	29.32	6
ALP normalization	Overall death and/or LT	9	1.5	1.38	1.63	62.7	0.006	21.47	8
Barcelona	Composite clinical outcome	10	2.54	2.05	3.14	80.2	<0.001	45.38	9
Barcelona	Liver-related events	9	2.49	1.95	3.18	68.1	0.001	25.11	8
Barcelona	Overall death and/or LT	5	3.5	2.66	4.6	85.1	<0.001	26.76	4
Rochester II	Composite clinical outcome	5	1.17	1.13	1.22	95.8	<0.001	94.88	4
Rochester II	Liver-related events	3	2.58	1.73	3.86	0	0.856	0.31	2
Rochester II	Overall death and/or LT	2	2.52	2.12	2.99	0	0.516	0.42	1
Toronto I	Composite clinical outcome	19	1.21	1.17	1.24	93.1	<0.001	259.27	18
Toronto I	Liver-related events	10	2.34	1.92	2.85	69.8	<0.001	29.78	9
Toronto I	Overall death and/or LT	16	1.91	1.78	2.06	81	<0.001	78.82	15
Toronto II	Composite clinical outcome	2	4.78	2.65	8.61	78.8	0.03	4.73	1
Toronto II	Liver-related events	2	4.78	2.65	8.61	78.8	0.03	4.73	1
Other ALP cut-offs	Composite clinical outcome	7	2.24	1.81	2.77	37.3	0.144	9.57	6
Other ALP cut-offs	Liver-related events	7	2.24	1.81	2.77	37.3	0.144	9.57	6
Other ALP cut-offs	Overall death and/or LT	6	2.26	2.18	2.34	0	0.552	3.98	5
**ALP Response (OR Estimates)**									
**Response**	**Outcomes**	**Number of studies**	**OR**	**LCI**	**UCI**	**I^2**	**p-value†**	**CocQ**	**df**
ALP normalization	Composite clinical outcome	4	1.52	1.28	1.8	56.6	0.075	6.92	3
ALP normalization	Liver-related events	1	3.7	0.37	36.86	0	<0.001	0	0
ALP normalization	Overall death and/or LT	4	1.52	1.28	1.8	50.8	0.107	6.1	3
Barcelona	Composite clinical outcome	12	2.4	1.83	3.15	65.5	<0.001	31.87	11
Barcelona	Liver-related events	9	2.27	1.69	3.05	72.1	<0.001	28.67	8

Barcelona	Overall death and/or LT	5	2.32	1.49	3.62	26.8	0.243	5.46	4
Rochester II	Composite clinical outcome	5	2.8	2.44	3.22	45	0.122	7.28	4
Rochester II	Liver-related events	2	2.81	1.6	4.93	0	0.895	0.02	1
Rochester II	Overall death and/or LT	2	2.79	2.42	3.22	85.6	0.009	6.92	1
Toronto I	Composite clinical outcome	12	2.58	2.19	3.03	64.4	0.001	30.9	11
Toronto I	Liver-related events	10	2.71	2.2	3.33	66.9	0.001	27.19	9
Toronto I	Overall death and/or LT	7	2.76	2.2	3.48	63.4	0.012	16.4	6
Toronto II	Composite clinical outcome	4	3.96	2.57	6.12	59	0.063	7.31	3
Toronto II	Liver-related events	4	3.96	2.57	6.12	59	0.063	7.31	3
Other ALP cut-offs	Composite clinical outcome	6	2.46	1.79	3.38	47	0.093	9.43	5
Other ALP cut-offs	Liver-related events	3	1.99	1.38	2.88	22.2	0.277	2.57	2
Other ALP cut-offs	Overall death and/or LT	2	3.76	1.85	7.63	0	0.725	0.12	1
**Composite Response (HR Estimates)**									
**Response**	**Outcomes**	**Number of studies**	**HR**	**LCI**	**UCI**	**I^2**	**p-value†**	**CocQ**	**df**
Modified Toronto	Composite clinical outcome	1	21.6	5.64	82.78	0	<0.001	0	0
Modified Toronto	Liver-related events	1	21.6	5.64	82.78	0	<0.001	0	0
Momah/Lindor	Composite clinical outcome	6	3.24	2.79	3.77	88.6	<0.001	43.76	5
Momah/Lindor	Liver-related events	4	3.17	2.6	3.86	85.5	<0.001	20.66	3
Momah/Lindor	Overall death and/or LT	3	3.77	3.17	4.49	92	<0.001	25.09	2
Paris I	Composite clinical outcome	21	3.73	3.29	4.22	78.8	<0.001	94.13	20
Paris I	Liver-related events	16	3.47	3.02	3.99	82.9	<0.001	87.62	15
Paris I	Overall death and/or LT	6	5.28	4.13	6.76	9.6	0.354	5.53	5
Paris II	Composite clinical outcome	22	3.17	2.74	3.68	54.4	0.001	46.09	21
Paris II	Liver-related events	17	3.63	2.96	4.46	59.7	<0.001	39.68	16
Paris II	Overall death and/or LT	7	3.5	2.69	4.54	0	0.871	2.48	6
Poise	Composite clinical outcome	7	3.37	2.41	4.71	0	0.897	2.23	6
Poise	Liver-related events	3	4.4	2.64	7.34	0	0.997	0.01	2
Poise	Overall death and/or LT	4	3.05	2.02	4.61	0	0.942	0.39	3
Rochester	Composite clinical outcome	3	2.46	1.06	5.69	68.1	0.043	6.28	2

Rochester	Liver-related events	2	3.44	1.28	9.28	78.8	0.03	4.72	1
Rochester	Overall death and/or LT	1	1.05	0.22	5.07	0	<0.001	0	0
Rotterdam	Composite clinical outcome	12	5.46	4.62	6.47	72.2	<0.001	39.63	11
Rotterdam	Liver-related events	10	5.71	4.73	6.9	76.8	<0.001	38.73	9
Rotterdam	Overall death and/or LT	4	4.75	3.33	6.77	0	0.922	0.48	3
ALP + Other composite	Composite clinical outcome	5	3.62	2.98	4.4	35.4	0.185	6.19	4
ALP + Other composite	Liver-related events	2	5.2	3.6	7.53	0	0.349	0.88	1
ALP + Other composite	Overall death and/or LT	2	3.18	2.51	4.03	0	0.776	0.08	1
**Composite Response (OR Estimates)**									
**Response**	**Outcomes**	**Number of studies**	**OR**	**LCI**	**UCI**	**I^2**	**p-value[†]**	**CocQ**	**df**
Modified Toronto	Composite clinical outcome	1	3.96	1.81	8.66	0	<0.001	0	0
Modified Toronto	Liver-related events	1	7.77	2.54	23.73	0	<0.001	0	0
Modified Toronto	Overall death and/or LT	1	2.93	1.09	7.82	0	<0.001	0	0
Paris I	Composite clinical outcome	17	5.16	4.23	6.3	8.4	0.356	17.47	16
Paris I	Liver-related events	12	5.25	4.09	6.73	16.6	0.281	13.19	11
Paris I	Overall death and/or LT	8	5.65	4.24	7.52	0	0.454	6.76	7
Paris II	Composite clinical outcome	14	3.87	3.26	4.6	66.4	<0.001	38.7	13
Paris II	Liver-related events	12	5.29	4.00	6.99	58	0.006	26.2	11
Paris II	Overall death and/or LT	5	3.35	2.71	4.14	56.5	0.056	9.19	4
Poise	Composite clinical outcome	3	2.7	2.04	3.58	0	0.448	1.61	2
Poise	Liver-related events	2	2.72	1.95	3.78	53.9	0.141	2.17	1
Poise	Overall death and/or LT	2	2.65	1.53	4.59	0	0.816	0.05	1
Rotterdam	Composite clinical outcome	13	3.58	2.81	4.56	65.5	<0.001	34.83	12
Rotterdam	Liver-related events	10	3.7	2.79	4.91	69.9	<0.001	29.91	9
Rotterdam	Overall death and/or LT	4	3.71	2.41	5.71	54.7	0.085	6.62	3
Other Composite Response	Composite clinical outcome	3	9.68	3.1	30.27	0	0.627	0.93	2

Other Composite Response	Liver-related events	2	2.87	0.96	8.61	23.7	0.252	1.31	1

ALP: Alkaline Phosphatase; CocQ: Cochran's Q; df: Degrees of freedom; HR: Hazard ratio; IV: Inverse variance; LCI: Lower confidence interval; LT: Liver transplantation; OR: Odds ratio; τ^2: Tau-square; UCI: Upper confidence interval

†p-values corresponding to I^2

Table S10: Sensitivity Analysis Results Using Random (DL + HKSJ) Method

Response	Outcomes	Number of studies	Estimate	LCI	UCI	I^2	p-value†	CocQ	df
**ALP response (HR estimates)**									
ALP normalization	Composite clinical outcome	11	1.46	1.2	1.77	88.7	<0.001	88.86	10
ALP normalization	Liver-related events	7	1.82	1.21	2.72	79.5	<0.001	29.32	6
ALP normalization	Overall death and/or LT	9	1.52	1.26	1.85	62.7	0.006	21.47	8
Barcelona	Composite clinical outcome	10	1.84	1.03	3.29	80.2	<0.001	45.38	9
Barcelona	Liver-related events	9	2.09	1.23	3.57	68.1	0.001	25.11	8
Barcelona	Overall death and/or LT	5	2.35	0.67	8.16	85.1	<0.001	26.76	4
Rochester II	Composite clinical outcome	5	2.22	1.27	3.89	95.8	<0.001	94.88	4
Rochester II	Liver-related events	3	2.58	1.82	3.65	0	0.856	0.31	2
Rochester II	Overall death and/or LT	2	2.52	1.22	5.17	0	0.516	0.42	1
Toronto I	Composite clinical outcome	19	1.87	1.57	2.23	93.1	<0.001	259.27	18
Toronto I	Liver-related events	10	2.56	1.51	4.34	69.8	<0.001	29.78	9
Toronto I	Overall death and/or LT	16	2	1.67	2.41	81	<0.001	78.82	15
Toronto II	Composite clinical outcome	2	4.8	0	19412.24	78.8	0.03	4.73	1
Toronto II	Liver-related events	2	4.8	0	19412.24	78.8	0.03	4.73	1
Other ALP cut-offs	Composite clinical outcome	7	2.38	1.54	3.67	37.3	0.144	9.57	6
Other ALP cut-offs	Liver-related events	7	2.38	1.54	3.67	37.3	0.144	9.57	6
Other ALP cut-offs	Overall death and/or LT	6	2.26	2.17	2.36	0	0.552	3.98	5
**ALP response (OR estimates)**									
ALP normalization	Composite clinical outcome	4	1.62	0.79	3.32	56.6	0.075	6.92	3

Response	Outcomes	Number of studies	Estima te	LCI	UCI	I^2	p-value†	CocQ	df
ALP normalization	Liver-related events	1	3.7	0.37	36.86	0	<0.001	0	0
ALP normalization	Overall death and/or LT	4	1.6	0.87	2.95	50.8	0.107	6.1	3
Barcelona	Composite clinical outcome	12	2.52	1.38	4.58	65.5	<0.001	31.87	11
Barcelona	Liver-related events	9	2.36	1.06	5.26	72.1	<0.001	28.67	8
Barcelona	Overall death and/or LT	5	2.55	1.12	5.77	26.8	0.243	5.46	4
Rochester II	Composite clinical outcome	5	3.37	1.84	6.17	45	0.122	7.28	4
Rochester II	Liver-related events	2	2.81	1.73	4.54	0	0.895	0.02	1
Rochester II	Overall death and/or LT	2	4.73	0	16280.31	85.6	0.009	6.92	1
Toronto I	Composite clinical outcome	12	3.1	2.01	4.77	64.4	0.001	30.9	11
Toronto I	Liver-related events	10	3.04	1.79	5.14	66.9	0.001	27.19	9
Toronto I	Overall death and/or LT	7	3.54	1.99	6.29	63.4	0.012	16.4	6
Toronto II	Composite clinical outcome	4	4.22	1.2	14.79	59	0.063	7.31	3
Toronto II	Liver-related events	4	4.22	1.2	14.79	59	0.063	7.31	3
Other ALP cut-offs	Composite clinical outcome	6	2.93	1.54	5.58	47	0.093	9.43	5
Other ALP cut-offs	Liver-related events	3	2.05	0.77	5.5	22.2	0.277	2.57	2
Other ALP cut-offs	Overall death and/or LT	2	3.76	0.74	18.97	0	0.725	0.12	1
**Composite response (HR estimates)**									
Modified Toronto	Composite clinical outcome	1	21.6	5.64	82.78	0	<0.001	0	0
Modified Toronto	Liver-related events	1	21.6	5.64	82.78	0	<0.001	0	0
Momah/Lindor	Composite clinical outcome	6	2.66	1.33	5.34	88.6	<0.001	43.76	5
Momah/Lindor	Liver-related events	4	2.98	1.28	6.95	85.5	<0.001	20.66	3
Momah/Lindor	Overall death and/or LT	3	2.77	0.33	23.15	92	<0.001	25.09	2

Response	Outcomes	Number of studies	Estimate	LCI	UCI	I^2	p-value†	CocQ	df
Paris I	Composite clinical outcome	21	4.11	3.25	5.19	78.8	<0.001	94.13	20
Paris I	Liver-related events	16	4.04	2.96	5.5	82.9	<0.001	87.62	15
Paris I	Overall death and/or LT	6	5.24	3.7	7.42	9.6	0.354	5.53	5
Paris II	Composite clinical outcome	22	3.71	2.89	4.77	54.4	0.001	46.09	21
Paris II	Liver-related events	17	4.5	3.15	6.43	59.7	<0.001	39.68	16
Paris II	Overall death and/or LT	7	3.5	2.84	4.31	0	0.871	2.48	6
Poise	Composite clinical outcome	7	3.37	2.61	4.35	0	0.897	2.23	6
Poise	Liver-related events	3	4.4	4.14	4.67	0	0.997	0.01	2
Poise	Overall death and/or LT	4	3.05	2.39	3.88	0	0.942	0.39	3
Rochester	Composite clinical outcome	3	1.78	0.07	47.49	68.1	0.043	6.28	2
Rochester	Liver-related events	2	2.13	0	17023681.58	78.8	0.03	4.72	1
Rochester	Overall death and/or LT	1	1.05	0.22	5.07	0	<0.001	0	0
Rotterdam	Composite clinical outcome	12	4.48	3.09	6.47	72.2	<0.001	39.63	11
Rotterdam	Liver-related events	10	4.37	2.71	7.07	76.8	<0.001	38.73	9
Rotterdam	Overall death and/or LT	4	4.75	3.77	5.99	0	0.922	0.48	3
ALP + Other composite	Composite clinical outcome	5	3.66	2.55	5.25	35.4	0.185	6.19	4
ALP + Other composite	Liver-related events	2	5.2	0.55	48.98	0	0.349	0.88	1
ALP + Other composite	Overall death and/or LT	2	3.18	2.05	4.93	0	0.776	0.08	1
**Composite response (OR estimates)**									
Modified Toronto	Composite clinical outcome	1	3.96	1.81	8.66	0	<0.001	0	0
Modified Toronto	Liver-related events	1	7.77	2.54	23.73	0	<0.001	0	0
Modified Toronto	Overall death and/or LT	1	2.93	1.09	7.82	0	<0.001	0	0

Response	Outcomes	Number of studies	Estimate	LCI	UCI	I^2	p-value†	CocQ	df
Paris I	Composite clinical outcome	17	5.23	4.13	6.62	8.4	0.356	17.47	16
Paris I	Liver-related events	12	5.31	3.83	7.36	16.6	0.281	13.19	11
Paris I	Overall death and/or LT	8	5.65	4.02	7.93	0	0.454	6.76	7
Paris II	Composite clinical outcome	14	5.18	3.09	8.68	66.4	<0.001	38.7	13
Paris II	Liver-related events	12	5.3	3.06	9.17	58	0.006	26.2	11
Paris II	Overall death and/or LT	5	6.9	1.76	27.01	56.5	0.056	9.19	4
Poise	Composite clinical outcome	3	2.7	1.55	4.71	0	0.448	1.61	2
Poise	Liver-related events	2	3.2	0.03	338.77	53.9	0.141	2.17	1
Poise	Overall death and/or LT	2	2.65	1.16	6.05	0	0.816	0.05	1
Rotterdam	Composite clinical outcome	13	3.89	2.15	7.07	65.5	<0.001	34.83	12
Rotterdam	Liver-related events	10	3.77	1.72	8.27	69.9	<0.001	29.91	9
Rotterdam	Overall death and/or LT	4	5.08	1.59	16.24	54.7	0.085	6.62	3
Other Composite Response	Composite clinical outcome	3	9.68	1.75	53.57	0	0.627	0.93	2
Other Composite Response	Liver-related events	2	3.16	0	20322.87	23.7	0.252	1.31	1

ALP: Alkaline Phosphatase; CocQ: Cochran's Q; df: Degrees of freedom; DL: DerSimonian and Laird; HKSJ: Hartung-Knapp-Sidik-Jonkman; HR: Hazard ratio; LCI: Lower confidence interval; LT: Liver transplantation; OR: Odds ratio; τ^2: Tau-square; UCI: Upper confidence interval

†p-values corresponding to I^2

τ^2 values for composite clinical outcome, liver-related events, and overall death and/or LT, respectively, were ALP response HR, 0.056267, 0.1370198, and 0.0788154; ALP response OR, 0.17786168, 0.2447175, and 0.15936784; composite response HR, 0.23264056, 0.32759218, and 0.06677084; and composite response OR, 0.18772134, 0.26030616, and 0.11377812. τ^2 denotes between-study variance under the DL random-effects model with HKSJ-adjusted inference and relates to the overall pooled analyses

Table S11: Influence Diagnostics for Pooled Analyses Exhibiting Substantial Heterogeneity (I^2 >75%)

ALP Response (HR Estimates)							
**Response**	**Outcomes**	**Number of studies**	**HR**	**LCI**	**UCI**	**I^2**	**p-value†**
Barcelona	Composite clinical outcome	10	1.84	1.1	3.06	80.2	<0.001
Barcelona	Overall death and/or LT	5	2.35	1.09	5.04	85.1	<0.001
Toronto I	Composite clinical outcome	18	1.96	1.62	2.36	78.8	<0.001
Toronto I	Overall death and/or LT	15	1.99	1.58	2.49	81.3	<0.001
Toronto II	Composite clinical outcome	2	4.8	1.33	17.28	78.8	0.03
Toronto II	Liver-related events	2	4.8	1.33	17.28	78.8	0.03
**Composite Response (HR Estimates)**							
**Response**	**Outcomes**	**Number of studies**	**HR**	**LCI**	**UCI**	**I^2**	**p-Value†**
Momah/Lindor	Liver-related events	4	2.98	1.61	5.5	85.5	<0.001
Rochester	Liver-related events	2	2.13	0.18	24.71	78.8	0.03

ALP: Alkaline phosphatase; HR: Hazard ratio; LCI: Lower confidence interval; LT: Liver transplantation; UCI: Upper confidence interval

†p-values corresponding to I^2

Table S12: Sensitivity Analysis After Inclusion of Full-Text Journal Articles

**ALP Response (HR Estimates)**							
**Response**	**Outcomes**	**Number of studies**	**HR**	**LCI**	**UCI**	**I^2**	**p-value[†]**
ALP normalization	Composite clinical outcome	7	1.35	1.16	1.57	90.3	<0.001
ALP normalization	Liver-related events	5	1.68	1.26	2.26	75.3	0.003
ALP normalization	Overall death and/or LT	6	1.5	1.23	1.84	70.1	0.005
Barcelona	Composite clinical outcome	9	1.94	1.13	3.33	80.6	<0.001
Barcelona	Liver-related events	8	2.28	1.4	3.72	66.8	0.004
Barcelona	Overall death and/or LT	5	2.35	1.09	5.04	85.1	<0.001
Rochester II	Composite clinical outcome	4	2.06	1.15	3.72	96.7	<0.001
Rochester II	Liver-related events	3	2.58	1.73	3.86	0	0.856
Rochester II	Overall death and/or LT	1	2.49	2.09	2.96	0	<0.001
Toronto I	Composite clinical outcome	13	1.82	1.36	2.44	91.5	<0.001
Toronto I	Liver-related events	9	2.11	1.56	2.96	41.2	0.092
Toronto I	Overall death and/or LT	10	2.14	1.84	2.49	21	0.25
Toronto II	Composite clinical outcome	2	4.8	1.33	17.28	78.8	0.03
Toronto II	Liver-related events	2	4.8	1.33	17.28	78.8	0.03
Other ALP cut-offs	Composite clinical outcome	5	2.38	1.78	3.17	25.7	0.25
Other ALP cut-offs	Liver-related events	5	2.38	1.78	3.17	25.7	0.25
Other ALP cut-offs	Overall death and/or LT	4	2.24	1.89	2.65	22.2	0.278
**ALP Response (OR Estimates)**							
**Response**	**Outcomes**	**Number of studies**	**OR**	**LCI**	**UCI**	**I^2**	**p-value[†]**
ALP normalization	Composite clinical outcome	2	2.94	0.27	32.46	65.9	**0.087**
ALP normalization	Liver-related events	1	3.7	0.37	36.86	0	<0.001
ALP normalization	Overall death and/or LT	2	1.96	0.46	8.35	53.9	0.141
Barcelona	Composite clinical outcome	10	2.16	1.43	3.27	42.2	0.076
Barcelona	Liver-related events	7	1.92	1.19	3.1	46.4	0.083
Barcelona	Overall death and/or LT	5	2.55	1.42	4.57	26.8	0.243

Rochester II	Composite clinical outcome	4	2.73	2.37	3.14	0	0.936
Rochester II	Liver-related events	2	2.81	1.6	4.93	0	0.895
Rochester II	Overall death and/or LT	1	2.71	2.34	3.13	0	<0.001
Toronto I	Composite clinical outcome	10	3.09	2.21	4.31	54.2	0.02
Toronto I	Liver-related events	8	3.47	2.32	5.17	48	0.061
Toronto I	Overall death and/or LT	5	2.98	1.81	4.92	60.1	0.04
Toronto II	Composite clinical outcome	4	4.22	1.93	9.23	59	0.063
Toronto II	Liver-related events	4	4.22	1.93	9.23	59	0.063
Other ALP cut-offs	Composite clinical outcome	4	3.74	2.03	6.87	36.1	0.196
Other ALP cut-offs	Liver-related events	2	2.57	1.51	4.37	0	0.344
Other ALP cut-offs	Overall death and/or LT	1	4.18	1.65	10.58	0	<0.001
**Composite Response (HR Estimates)**							
**Response**	**Outcomes**	**Number of studies**	**HR**	**LCI**	**UCI**	**I^2**	**p-value†**
Modified Toronto	Composite clinical outcome	1	21.6	5.64	82.78	0	<0.001
Modified Toronto	Liver-related events	1	21.6	5.64	82.78	0	<0.01
Momah/Lindor	Composite clinical outcome	4	1.95	1.07	3.55	75.4	0.007
Momah/Lindor	Liver-related events	3	2.5	1.29	4.86	70.4	0.034
Momah/Lindor	Overall death and/or LT	1	0.94	0.53	1.66	0	<0.001
Paris I	Composite clinical outcome	13	3.82	2.55	5.73	82.5	<0.001
Paris I	Liver-related events	11	3.7	2.3	5.95	82.5	<0.001
Paris I	Overall death and/or LT	4	4.69	3.44	6.38	0	0.435
Paris II	Composite clinical outcome	19	3.69	2.85	4.78	60	<0.001
Paris II	Liver-related events	15	4.5	3.07	6.6	64.1	<0.001
Paris II	Overall death and/or LT	6	3.45	2.65	4.49	0	0.835
Poise	Composite clinical outcome	2	3.8	1.8	8.05	0	0.716
Poise	Liver-related events	1	4.35	1.54	12.3	0	<0.001
Poise	Overall death and/or LT	-	-	-	-	-	-
Rochester	Composite clinical outcome	3	1.78	0.37	8.55	68.1	0.043
Rochester	Liver-related events	2	2.13	0.18	24.71	78.8	0.03
Rochester	Overall death and/or LT	1	1.05	0.22	5.07	0	0.835

Rotterdam	Composite clinical outcome	9	3.93	2.51	6.16	66.8	0.002
Rotterdam	Liver-related events	8	3.75	2.21	6.36	70.7	0.001
Rotterdam	Overall death and/or LT	3	5.62	3.07	10.3	0	0.986
ALP + Other composite	Composite clinical outcome	3	4.09	2.74	4.36	57.3	0.096
ALP + Other composite	Liver-related events	2	5.2	3.6	7.53	0	0.349
ALP + Other composite	Overall death and/or LT	1	3.26	2.44	4.36	0	<0.001
**Composite Response (OR Estimates)**							
**Response**	**Outcomes**	**Number of studies**	**OR**	**LCI**	**UCI**	**I^2**	**p-value†**
Modified Toronto	Composite clinical outcome	1	3.96	1.81	8.66	0	<0.001
Modified Toronto	Liver-related events	1	7.77	2.54	23.73	0	<0.001
Modified Toronto	Overall death and/or LT	1	2.93	1.09	7.82	0	<0.001
Paris I	Composite clinical outcome	14	5.5	4.17	7.26	24.8	0.186
Paris I	Liver-related events	10	5.57	3.86	8.02	30.5	0.165
Paris I	Overall death and/or LT	7	5.96	4.26	8.34	10.1	0.352
Paris II	Composite clinical outcome	12	4.21	2.91	6.09	46.9	0.036
Paris II	Liver-related events	10	4.31	2.81	6.6	37.7	0.107
Paris II	Overall death and/or LT	5	6.9	2.67	17.85	56.5	0.056
Rotterdam	Composite clinical outcome	11	3.56	2.15	5.89	66.2	0.001
Rotterdam	Liver-related events	8	3.31	1.71	6.41	71.8	<0.001
Rotterdam	Overall death and/or LT	4	5.08	2.32	11.09	54.7	0.085
Other Composite Response	Composite clinical outcome	3	9.68	3.1	30.27	0	0.627
Other Composite Response	Liver-related events	2	3.16	0.82	12.22	23.7	0.252

ALP: Alkaline phosphatase; HR: Hazard ratio; LCI: Lower confidence interval; LT: Liver transplantation; OR: Odds ratio; UCI: Upper confidence interval

†p-values corresponding to I^2

Table S13: Sensitivity Analysis After Including Only High-Quality Studies

ALP Response (HR Estimates)							
**Response**	**Outcomes**	**Number of studies**	**HR**	**LCI**	**UCI**	**I^2**	**p-value†**
ALP normalization	Composite clinical outcome	10	1.47	1.26	1.72	89.6	<0.001
ALP normalization	Liver-related events	7	1.82	1.41	2.34	79.5	<0.001
ALP normalization	Overall death and/or LT	8	1.55	1.28	1.86	66.7	0.004
Barcelona	Composite clinical outcome	10	1.84	1.1	3.06	80.2	0.001
Barcelona	Liver-related events	9	2.09	1.31	3.34	68.1	0.001
Barcelona	Overall death and/or LT	5	2.35	1.09	5.04	85.1	0.001
Rochester II	Composite clinical outcome	5	2.22	1.28	3.84	95.8	0.001
Rochester II	Liver-related events	3	2.58	1.73	3.86	0	0.856
Rochester II	Overall death and/or LT	2	2.52	2.12	2.99	0	0.516
Toronto I	Composite clinical outcome	15	1.87	1.44	2.43	91.4	0.001
Toronto I	Liver-related events	10	2.56	1.7	3.85	69.8	0.001
Toronto I	Overall death and/or LT	12	2.08	1.81	2.39	28.2	0.169
Toronto II	Composite clinical outcome	2	4.8	1.33	17.28	78.8	0.03
Toronto II	Liver-related events	2	4.8	1.33	17.28	78.8	0.03
Other ALP cut-offs	Composite clinical outcome	6	2.24	1.68	3	32.2	0.194
Other ALP cut-offs	Liver-related events	7	2.38	1.75	3.23	37.3	0.144
Other ALP cut-offs	Overall death and/or LT	6	2.26	2.18	2.34	0	0.552
**ALP Response (OR Estimates)**							
**Response**	**Outcomes**	**Number of studies**	**OR**	**LCI**	**UCI**	**I^2**	**p-value†**
ALP normalization	Composite clinical outcome	4	1.62	1.13	2.31	56.6	0.075
ALP normalization	Liver-related events	1	3.7	0.37	36.86	0	0.001
ALP normalization	Overall death and/or LT	4	1.6	1.16	2.22	50.8	0.107
Barcelona	Composite clinical outcome	12	2.52	1.53	4.14	65.5	0.001
Barcelona	Liver-related events	9	2.36	1.28	4.34	72.1	0.001
Barcelona	Overall death and/or LT	5	2.55	1.42	4.57	26.8	0.243

Rochester II	Composite clinical outcome	4	3.39	2.1	5.46	56.8	0.074
Rochester II	Liver-related events	2	2.81	1.6	4.93	0	0.895
Rochester II	Overall death and/or LT	2	4.73	1.35	16.62	85.6	0.009
Toronto I	Composite clinical outcome	11	3.21	2.28	4.53	67.2	<0.001
Toronto I	Liver-related events	10	3.04	2	4.61	66.9	0.001
Toronto I	Overall death and/or LT	7	3.54	2.24	5.6	63.4	0.012
Toronto II	Composite clinical outcome	4	4.22	1.93	9.23	59	0.063
Toronto II	Liver-related events	4	4.22	1.93	9.23	59	0.063
Other ALP cut-offs	Composite clinical outcome	3	2.68	1.66	4.33	0	0.597
Other ALP cut-offs	Liver-related events	2	2.57	1.51	4.37	0	0.344
Other ALP cut-offs	Overall death and/or LT	2	3.76	1.85	7.63	0	0.725
**Composite Response (HR Estimates)**							
**Response**	**Outcomes**	**Number of studies**	**HR**	**LCI**	**UCI**	**I^2**	**p-value†**
Modified Toronto	Composite clinical outcome	1	21.6	5.64	82.78	0	<0.001
Modified Toronto	Liver-related events	1	21.6	5.64	82.78	0	<0.001
Momah/Lindor	Composite clinical outcome	4	1.95	1.07	3.55	75.4	0.007
Momah/Lindor	Liver-related events	3	2.5	1.29	4.86	70.4	0.034
Momah/Lindor	Overall death and/or LT	1	0.94	0.53	1.66	0	<0.001
Paris I	Composite clinical outcome	15	3.83	2.67	5.49	80.6	<0.001
Paris I	Liver-related events	12	3.61	2.33	5.59	80.7	<0.001
Paris I	Overall death and/or LT	5	4.81	3.67	6.31	0	0.582
Paris II	Composite clinical outcome	21	3.73	2.91	4.78	56.6	<0.001
Paris II	Liver-related events	17	4.5	3.16	6.41	59.7	<0.001
Paris II	Overall death and/or LT	7	3.5	2.69	4.54	0	0.871
Poise	Composite clinical outcome	6	3.28	2.3	4.68	0	0.842
Poise	Liver-related events	2	4.35	2.4	7.89	0	0.999
Poise	Overall death and/or LT	4	3.05	2.02	4.61	0	0.942
Rochester	Composite clinical outcome	3	1.78	0.37	8.55	68.1	0.043
Rochester	Liver-related events	2	2.13	0.18	24.71	78.8	0.03
Rochester	Overall death and/or LT	1	1.05	0.22	5.07	0	<0.001

Rotterdam	Composite clinical outcome	11	4.1	2.92	5.76	58.9	0.007
Rotterdam	Liver-related events	9	3.9	2.46	6.19	67	0.002
Rotterdam	Overall death and/or LT	4	4.75	3.33	6.77	0	0.922
ALP + Other composite	Composite clinical outcome	5	3.66	2.8	4.78	35.4	0.185
ALP + Other composite	Liver-related events	2	5.2	3.6	7.53	0	0.349
ALP + Other composite	Overall death and/or LT	2	3.18	2.51	4.03	0	0.776
**Composite Response (OR Estimates)**							
**Response**	**Outcomes**	**Number of studies**	**OR**	**LCI**	**UCI**	**I^2**	**p-value†**
Modified Toronto	Composite clinical outcome	1	3.96	1.81	8.66	0	<0.001
Modified Toronto	Liver-related events	1	7.77	2.54	23.73	0	<0.001
Modified Toronto	Overall death and/or LT	1	2.93	1.09	7.82	0	<0.001
Paris I	Composite clinical outcome	15	5.43	4.18	7.04	19.2	0.239
Paris I	Liver-related events	11	5.48	3.92	7.65	23.1	0.224
Paris I	Overall death and/or LT	7	5.96	4.26	8.34	10.1	0.352
Paris II	Composite clinical outcome	13	4.26	2.99	6.09	43.9	0.045
Paris II	Liver-related events	11	4.38	2.93	6.55	32.5	0.139
Paris II	Overall death and/or LT	5	6.9	2.67	17.85	56.5	0.056
Poise	Composite clinical outcome	3	2.7	2.04	3.58	0	0.448
Poise	Liver-related events	2	3.2	1.56	6.57	53.9	0.141
Poise	Overall death and/or LT	2	2.65	1.53	4.59	0	0.816
Rotterdam	Composite clinical outcome	12	3.75	2.27	6.2	65.1	<0.001
Rotterdam	Liver-related events	9	3.6	1.87	6.92	70.1	<0.001
Rotterdam	Overall death and/or LT	4	5.08	2.32	11.09	54.7	0.085
Other Composite Response	Composite clinical outcome	3	9.68	3.1	30.27	0	0.627
Other Composite Response	Liver-related events	1	9.09	0.95	86.72	0	<0.001

†p-values corresponding to I^2

ALP: Alkaline phosphatase; HR: Hazard ratio; LCI: Lower confidence interval; LT: Liver transplantation; OR: Odds ratio;

UCI: Upper confidence interval

Figure S9: Funnel Plots for Studies Evaluating Associations Between ALP Response and (A) Composite Clinical Outcomes HR Estimates, (B) Composite Clinical Outcomes OR Estimates, (C) Any Liver-Related Events HR Estimates, (D) Any Liver-Related Events OR Estimates, (E) and Overall Death and/or LT HR Estimates

(A)

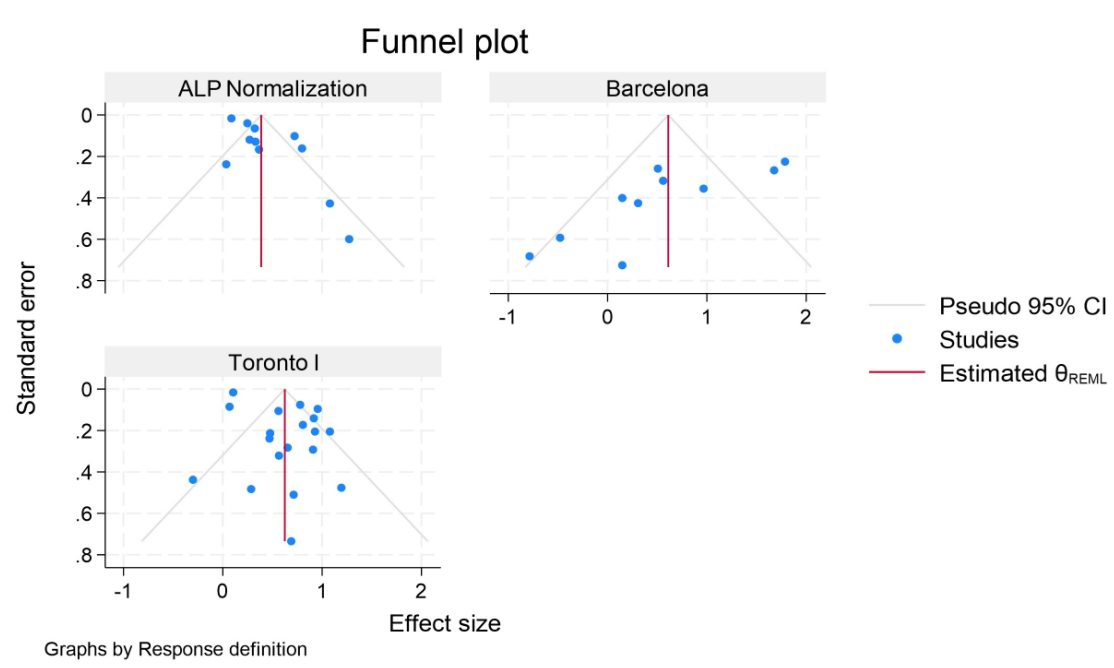

(B)

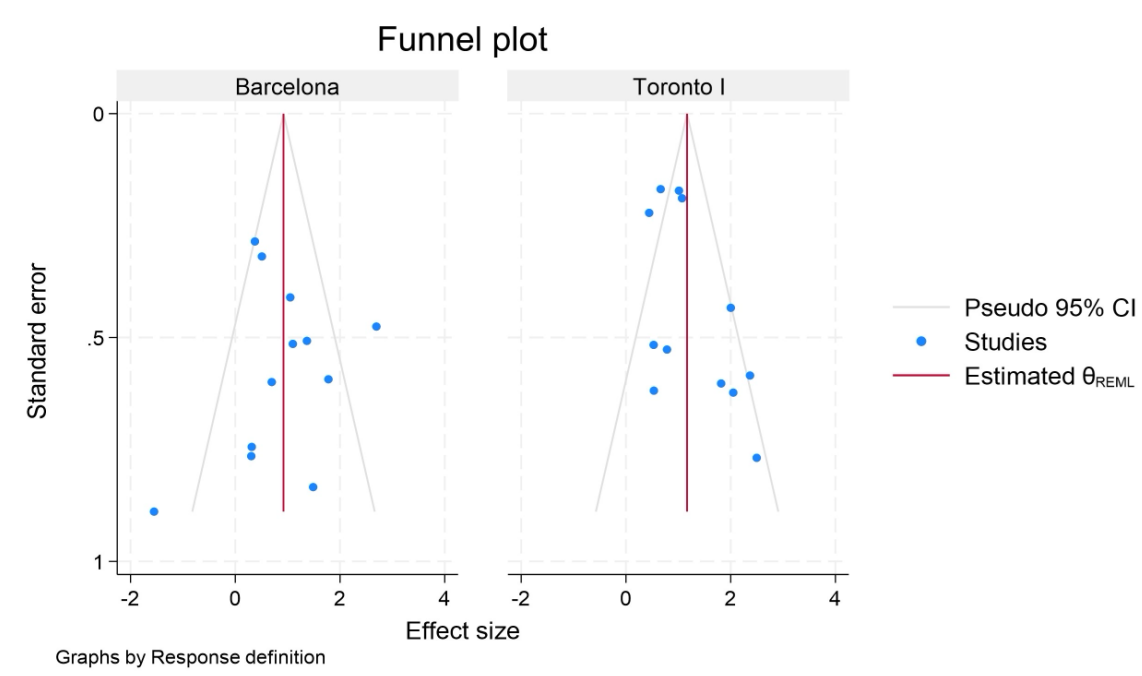

(C)

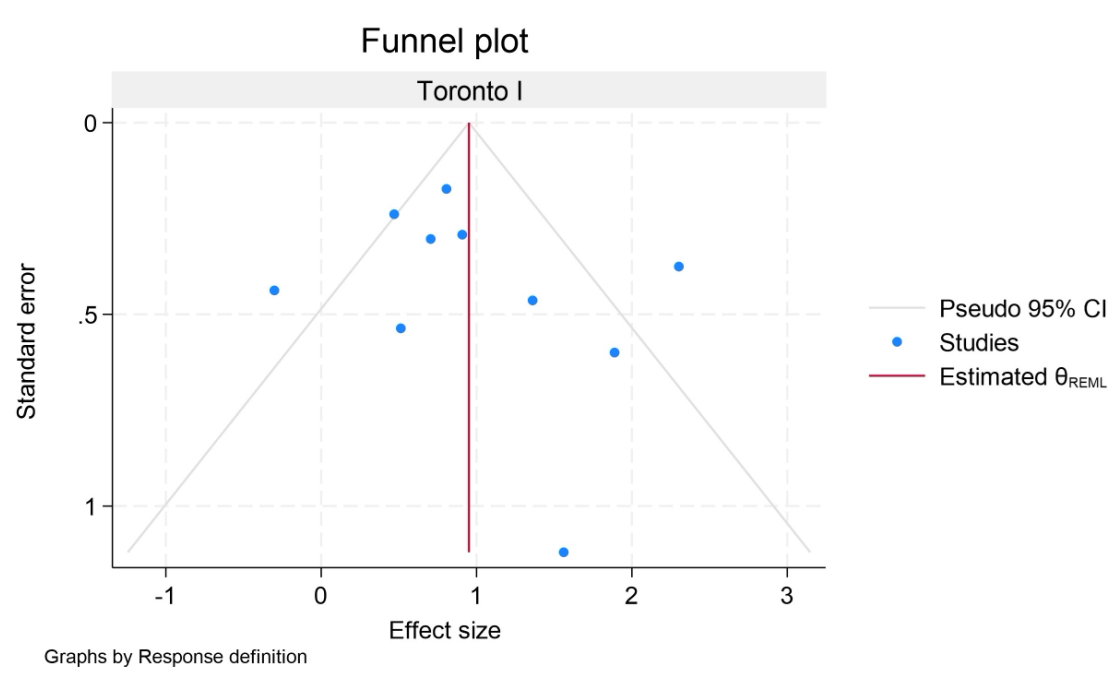

(D)

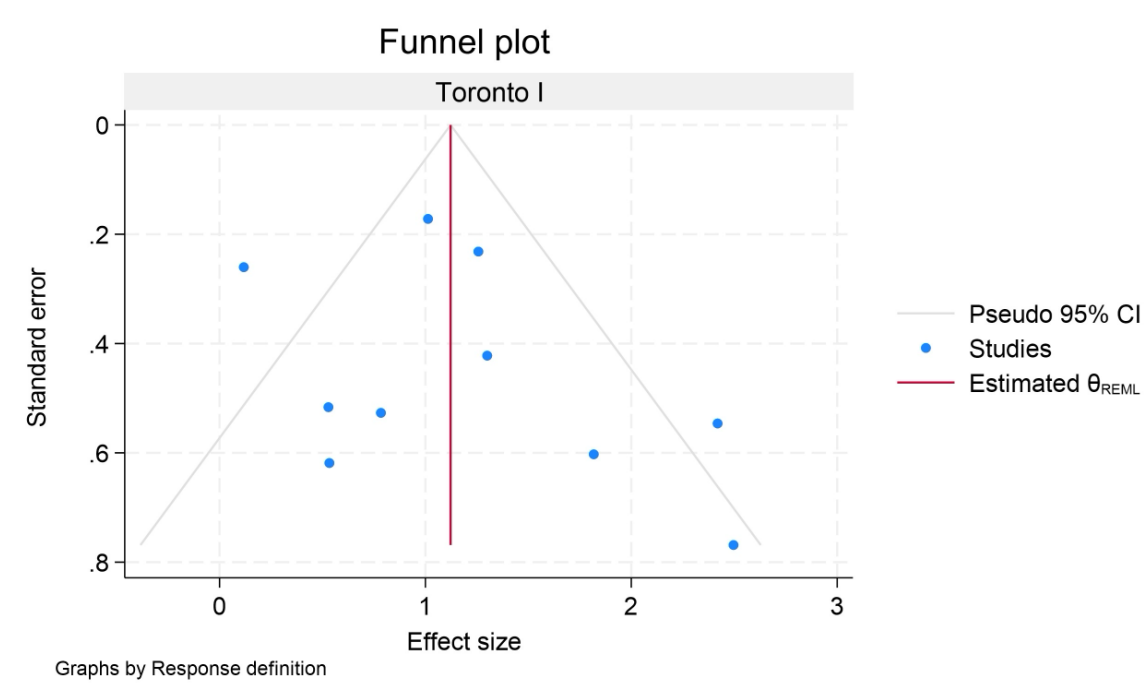

(E)

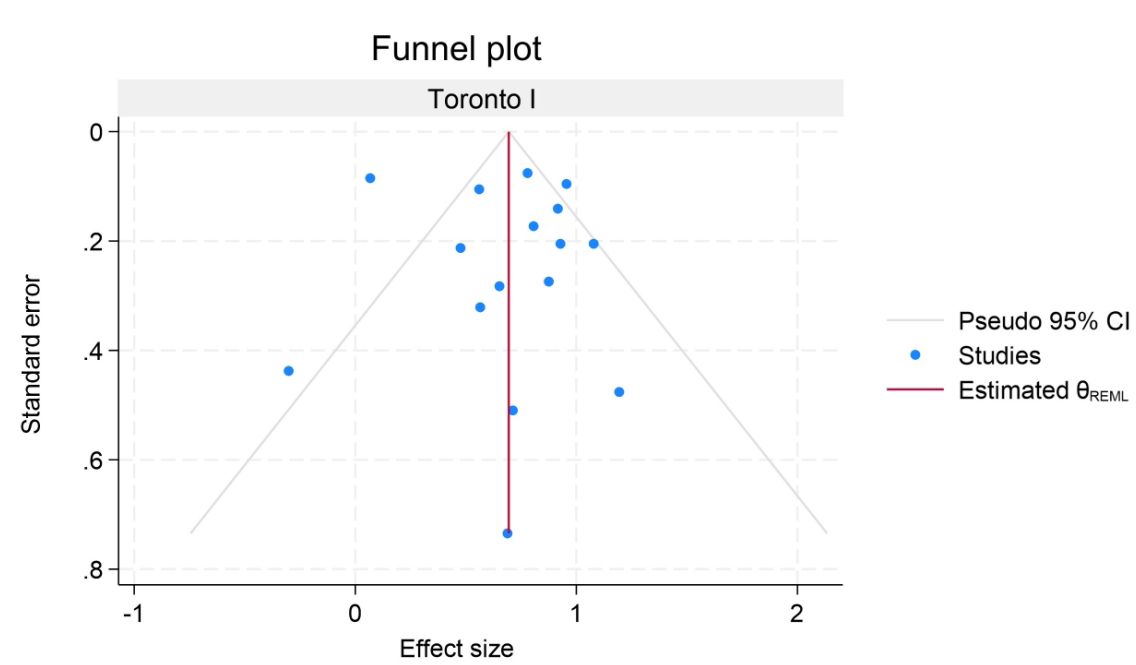

Figure S10: Funnel Plots for Studies Evaluating Associations Between Composite Response and (A) Composite Clinical Outcomes HR Estimates, (B) Composite Clinical Outcomes OR Estimates, (C) Any Liver-Related Events HR Estimates, (D) Any Liver-Related Events OR Estimates,

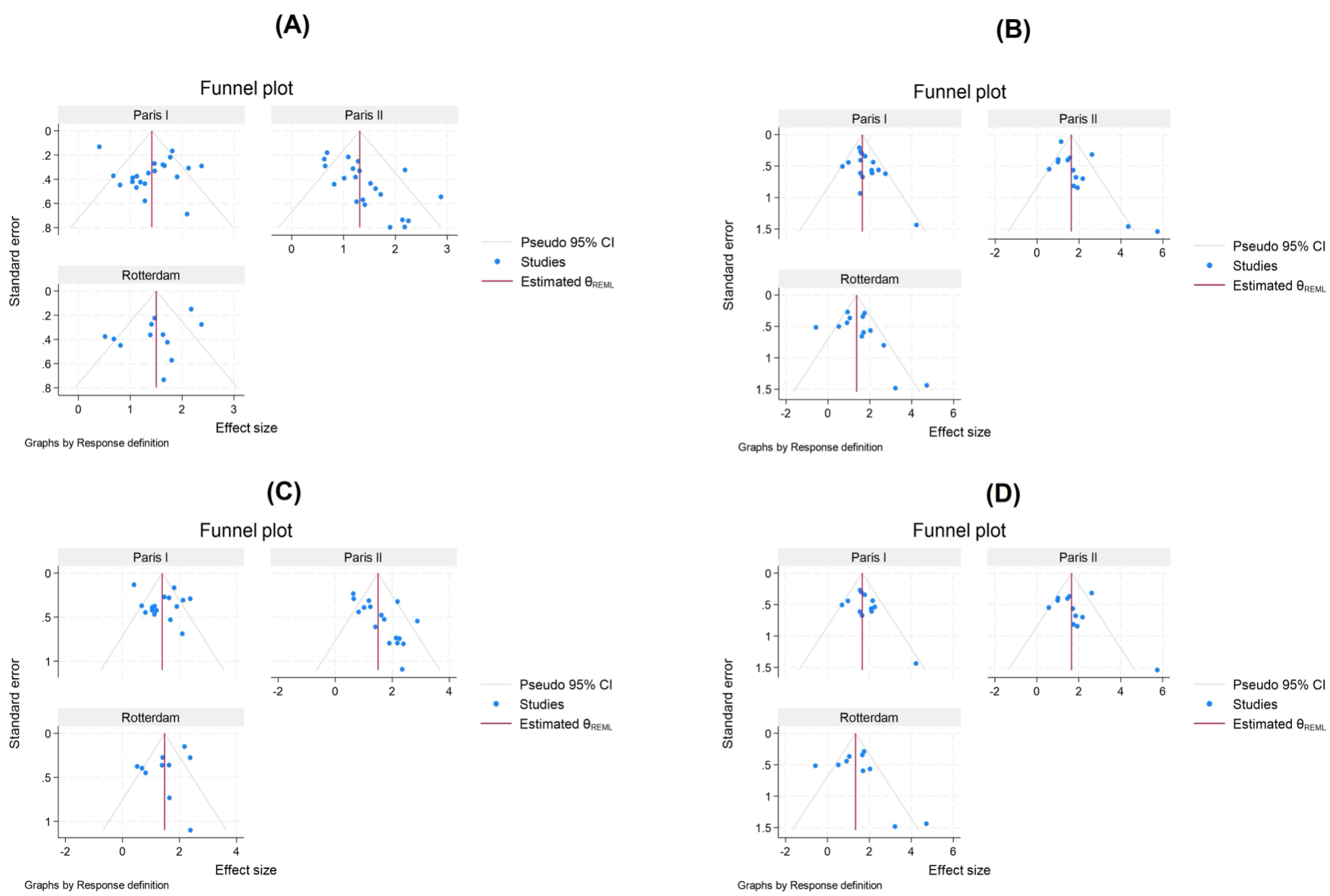

Table S14: Sensitivity Analysis Restricted to Studies with a Follow-up Duration Greater Than 12 Months, Excluding Studies with ≤12 Months of Follow-Up or No Reported Follow-up Duration

Response	Outcomes	Number of studies	Estimate (HR/OR)	LCI	UCI	I^2	p-value†
**ALP Response (HR Estimates)**							
ALP normalization	Composite clinical outcome	9	1.38	1.2	1.59	88.4	<0.001
ALP normalization	Liver-related events	5	1.68	1.26	2.26	75.3	0.003
ALP normalization	Overall death and/or LT	7	1.48	1.25	1.75	64.6	0.009
Barcelona	Composite clinical outcome	7	2.48	1.46	4.21	79.7	<0.001
Barcelona	Liver-related events	6	2.48	1.46	4.19	72.8	0.002
Barcelona	Overall death and/or LT	3	3.93	1.94	7.95	81.5	0.005
Rochester II	Composite clinical outcome	5	2.22	1.28	3.84	95.8	<0.001
Rochester II	Liver-related events	3	2.58	1.73	3.86	0	0.856
Rochester II	Overall death and/or LT	2	2.52	2.12	2.99	0	0.516
Toronto I	Composite clinical outcome	15	1.91	1.45	2.52	92.1	<0.001
Toronto I	Liver-related events	9	2.38	1.58	3.6	70	<0.001
Toronto I	Overall death and/or LT	13	2.28	2.03	2.56	22.3	0.218
Toronto II	Composite clinical outcome	1	9.24	4	21.35	0	<0.001
Toronto II	Liver-related events	1	9.24	4	21.35	0	<0.001
Other ALP cut-offs	Composite clinical outcome	6	2.26	1.66	3.07	36.5	0.164
Other ALP cut-offs	Liver-related events	6	2.26	1.66	3.07	36.5	0.164
Other ALP cut-offs	Overall death and/or LT	4	2.26	1.89	2.7	22.1	0.278
**ALP Response (OR Estimates)**							
ALP normalization	Composite clinical outcome	3	1.54	1.14	2.09	54.4	0.112
ALP normalization	Overall death and/or LT	3	1.54	1.14	2.09	54.4	0.112
Barcelona	Composite clinical outcome	7	1.92	1.15	3.19	35.1	0.16
Barcelona	Liver-related events	5	1.78	0.89	3.53	51.7	0.082
Barcelona	Overall death and/or LT	2	2.35	1.02	5.41	0	0.389

Response	Outcomes	Number of studies	Estimate (HR/OR)	LCI	UCI	I^2	p-value[†]
Rochester II	Composite clinical outcome	5	3.37	2.24	5.08	45	0.122
Rochester II	Liver-related events	2	2.81	1.6	4.93	0	0.895
Rochester II	Overall death and/or LT	2	4.73	1.35	16.62	85.6	0.009
Toronto I	Composite clinical outcome	9	2.89	2.13	3.91	48.9	0.047
Toronto I	Liver-related events	7	3.02	2.19	4.17	23.6	0.249
Toronto I	Overall death and/or LT	5	3.39	2	5.72	71.5	0.007
Toronto II	Composite clinical outcome	3	4.92	1.56	15.51	61.8	0.073
Toronto II	Liver-related events	3	4.92	1.56	15.51	61.8	0.073
Other ALP cut-offs	Composite clinical outcome	6	2.93	1.8	4.76	47	0.093
Other ALP cut-offs	Liver-related events	3	2.05	1.33	3.19	22.2	0.277
Other ALP cut-offs	Overall death and/or LT	2	3.76	1.85	7.63	0	0.725
**Composite Response (HR Estimates)**							
Modified Toronto	Composite clinical outcome	1	21.6	5.64	82.78	0	<0.001
Modified Toronto	Liver-related events	1	21.6	5.64	82.78	0	<0.001
Momah/Lindor	Composite clinical outcome	6	2.66	1.62	4.37	88.6	<0.001
Momah/Lindor	Liver-related events	4	2.98	1.61	5.5	85.5	<0.001
Momah/Lindor	Overall death and/or LT	3	2.77	1.42	5.42	92	<0.001
Paris I	Composite clinical outcome	17	4.37	3.51	5.44	43.5	0.029
Paris I	Liver-related events	13	4.23	3.19	5.6	55	0.009
Paris I	Overall death and/or LT	3	4.97	3.58	6.9	0	0.432
Paris II	Composite clinical outcome	18	3.77	2.89	4.92	57.5	0.001
Paris II	Liver-related events	13	4.47	3.04	6.58	60.2	0.003
Paris II	Overall death and/or LT	5	3.49	2.65	4.61	0	0.663
Poise	Composite clinical outcome	6	3.14	2.15	4.58	0	0.898
Poise	Liver-related events	2	4.45	2.16	9.15	0	0.951
Poise	Overall death and/or LT	3	2.87	1.74	4.74	0	0.892
Rochester	Composite clinical outcome	1	6.58	2.08	20.82	0	<0.001
Rochester	Liver-related events	1	6.58	2.08	20.82	0	<0.001
Rotterdam	Composite clinical outcome	9	4.91	3.3	7.31	70.4	<0.001

Response	Outcomes	Number of studies	Estimate (HR/OR)	LCI	UCI	I^2	p-value†
Rotterdam	Liver-related events	8	4.81	3.1	7.46	74.1	<0.001
Rotterdam	Overall death and/or LT	2	5.46	2.66	11.2	0	0.933
ALP + Other composite	Composite clinical outcome	4	3.89	2.76	5.48	42.9	0.154
ALP + Other composite	Liver-related events	2	5.2	3.6	7.53	0	0.349
ALP + Other composite	Overall death and/or LT	1	3.26	2.44	4.36	0	<0.001
**Composite Response (OR Estimates)**							
Modified Toronto	Composite clinical outcome	1	3.96	1.81	8.66	0	<0.001
Modified Toronto	Liver-related events	1	21.6	5.64	82.78	0	<0.001
Modified Toronto	Overall death and/or LT	1	2.93	1.09	7.82	0	<0.001
Paris I	Composite clinical outcome	14	5.16	4	6.66	16.2	0.277
Paris I	Liver-related events	11	3.7	2.3	5.95	82.5	<0.001
Paris I	Overall death and/or LT	6	5.17	3.78	7.09	0	0.506
Paris II	Composite clinical outcome	11	5.07	3.05	8.43	72.5	<0.001
Paris II	Liver-related events	15	4.5	3.07	6.6	64.1	<0.001
Paris II	Overall death and/or LT	3	5.48	1.88	15.96	65.1	0.057
Poise	Composite clinical outcome	2	3.15	1.95	5.08	0.4	0.316
Poise	Liver-related events	1	4.35	1.54	12.3	0	<0.001
Poise	Overall death and/or LT	2	2.65	1.53	4.59	0	0.816
Rotterdam	Composite clinical outcome	11	3.9	2.34	6.51	69.4	<0.001
Rotterdam	Liver-related events	8	3.75	2.21	6.36	70.7	0.01
Rotterdam	Overall death and/or LT	4	5.08	2.32	11.09	54.7	0.085
Rochester	Liver-related events	2	2.13	0.18	24.71	78.8	0.03
Other Composite Response	Composite clinical outcome	2	6.15	1.31	28.98	0	0.641
Other Composite Response	Liver-related events	2	5.2	3.6	7.53	0	0.349

†p-values corresponding to I^2

ALP: Alkaline phosphatase; HR: Hazard ratio; LCI: Lower confidence interval; LT: Liver transplantation; OR: Odds ratio;

UCI: Upper confidence interval

Table S15: Sensitivity Analysis Excluding Studies with Study Durations ≤12 Months or No Reported Study Duration

Response	Outcomes	Number of studies	Estimates (HR/OR)	LCI	UCI	I^2	p-value†
**ALP Response (HR Estimates)**							
ALP normalization	Composite clinical outcome	6	1.35	1.14	1.59	89	<0.001
ALP normalization	Liver-related events	7	1.82	1.41	2.34	79.5	<0.001
ALP normalization	Overall death and/or LT	5	1.5	1.14	1.99	57.8	0.05
Barcelona	Composite clinical outcome	9	2.04	1.22	3.4	79.8	<0.001
Barcelona	Liver-related events	8	2.38	1.52	3.73	63.8	0.007
Barcelona	Overall death and/or LT	5	2.35	1.09	5.04	85.1	<0.001
Rochester II	Composite clinical outcome	3	1.92	0.94	3.92	86.8	<0.001
Rochester II	Liver-related events	3	2.58	1.73	3.86	0	0.856
Toronto I	Composite clinical outcome	14	1.75	1.37	2.24	91.8	<0.001
Toronto I	Liver-related events	8	2.08	1.52	2.85	46.4	0.071
Toronto I	Overall death and/or LT	11	1.89	1.45	2.47	85.2	<0.001
Toronto II	Composite clinical outcome	1	9.24	4	21.35	0	<0.001
Toronto II	Liver-related events	1	9.24	4	21.35	0	<0.001
Toronto (not defined)	Overall death and/or LT	2	1.97	1.5	2.59	0	0.515
Other ALP cut-offs	Composite clinical outcome	6	2.24	1.68	3	32.2	0.194
Other ALP cut-offs	Liver-related events	6	2.24	1.68	3	32.2	0.194
Other ALP cut-offs	Overall death and/or LT	4	2.18	1.66	2.86	18.9	0.296
**ALP Response (OR Estimates)**							
ALP normalization	Composite clinical outcome	2	3.19	0.4	25.67	58.1	0.122
ALP normalization	Liver-related events	1	3.7	0.37	36.86	0	<0.001
ALP normalization	Overall death and/or LT	2	2.08	0.72	5.98	35.5	0.213
Barcelona	Composite clinical outcome	8	2.12	1.32	3.4	35.6	0.144
Barcelona	Liver-related events	6	2.06	1.12	3.78	50.4	0.073

Response	Outcomes	Number of studies	Estimates (HR/OR)	LCI	UCI	I^2	p-value†
Barcelona	Overall death and/or LT	3	2.64	1.18	5.9	0	0.415
Rochester II	Composite clinical outcome	3	2.99	1.79	4.99	0	0.866
Rochester II	Liver-related events	2	2.81	1.6	4.93	0	0.895
Toronto I	Composite clinical outcome	10	2.63	1.95	3.56	55.9	0.016
Toronto I	Liver-related events	9	2.8	1.86	4.21	65.7	0.003
Toronto I	Overall death and/or LT	6	3.03	1.97	4.66	53.8	0.055
Toronto II	Composite clinical outcome	2	4.61	0.73	29.28	80.8	0.023
Toronto II	Liver-related events	2	4.61	0.73	29.28	80.8	0.023
Other ALP cut-offs	Composite clinical outcome	3	3.85	1.72	8.64	55	0.108
Other ALP cut-offs	Liver-related events	1	2.27	1.26	4.09	0	<0.001
Other ALP cut-offs	Overall death and/or LT	1	4.18	1.65	10.58	0	<0.001
**Composite Response (HR Estimates)**							
Modified Toronto	Composite clinical outcome	1	21.6	5.64	82.78	0	<0.001
Modified Toronto	Liver-related events	1	21.6	5.64	82.78	0	<0.001
Momah/Lindor	Composite clinical outcome	3	2.18	0.81	5.85	83.2	0.003
Momah/Lindor	Liver-related events	2	3.44	1.72	6.86	38.1	0.204
Momah/Lindor	Overall death and/or LT	1	0.94	0.53	1.66	0	<0.001
Paris I	Composite clinical outcome	15	4.22	3.31	5.37	44.4	0.033
Paris I	Liver-related events	12	3.95	2.86	5.47	51.6	0.019
Paris I	Overall death and/or LT	5	4.81	3.67	6.31	0	0.582
Paris II	Composite clinical outcome	17	3.69	2.93	4.63	36.9	0.064
Paris II	Liver-related events	13	4.59	3.44	6.13	13	0.315
Paris II	Overall death and/or LT	6	3.45	2.65	4.49	0	0.835
Poise	Composite clinical outcome	3	3.77	2.25	6.33	0	0.861
Poise	Liver-related events	1	4.35	2.11	8.99	0	<0.001
Poise	Overall death and/or LT	2	3.37	1.87	6.09	0	0.917
Rochester	Composite clinical outcome	3	1.78	0.37	8.55	68.1	0.043
Rochester	Liver-related events	2	2.13	0.18	24.71	78.8	0.03

Response	Outcomes	Number of studies	Estimates (HR/OR)	LCI	UCI	I^2	p-value†
Rochester	Overall death and/or LT	1	1.05	0.22	5.07	0	<0.001
Rotterdam	Composite clinical outcome	10	4.53	3.3	6.21	48.3	0.043
Rotterdam	Liver-related events	8	4.41	2.82	6.9	60	0.014
Rotterdam	Overall death and/or LT	4	4.75	3.33	6.77	0	0.922
ALP + Other composite	Composite clinical outcome	4	3.76	2.78	5.07	48.2	0.122
ALP + Other composite	Liver-related events	2	5.2	3.6	7.53	0	0.349
ALP + Other composite	Overall death and/or LT	2	3.18	2.51	4.03	0	0.776
**Composite Response (OR Estimates)**							
Modified Toronto	Composite clinical outcome	1	3.96	1.81	8.66	0	<0.001
Modified Toronto	Liver-related events	1	7.77	2.54	23.73	0	<0.001
Modified Toronto	Overall death and/or LT	1	2.93	1.09	7.82	0	<0.001
Paris I	Composite clinical outcome	13	5.65	4.11	7.76	30.4	0.14
Paris I	Liver-related events	9	5.22	3.56	7.64	32.5	0.158
Paris I	Overall death and/or LT	6	5.64	3.81	8.34	11.5	0.342
Paris II	Composite clinical outcome	9	3.84	2.69	5.47	27.3	0.202
Paris II	Liver-related events	9	5.16	2.8	9.51	68.6	0.001
Paris II	Overall death and/or LT	4	5.34	2.14	13.33	50.9	0.106
Poise	Composite clinical outcome	2	2.91	1.82	4.65	36.3	0.21
Poise	Liver-related events	1	5.46	2.04	14.62	0	<0.001
Poise	Overall death and/or LT	1	2.98	0.96	9.23	0	<0.001
Rotterdam	Composite clinical outcome	10	3.74	2.1	6.66	69.5	<0.001
Rotterdam	Liver-related events	8	3.72	1.9	7.26	74.7	0.001
Rotterdam	Overall death and/or LT	4	5.08	2.32	11.09	54.7	0.085
Other Composite Response	Composite clinical outcome	1	9.09	0.95	86.72	0	<0.001
Other Composite Response	Liver-related events	2	3.16	0.82	12.22	23.7	0.252

†p-values corresponding to I^2

ALP: Alkaline phosphatase; HR: Hazard ratio; LCI: Lower confidence interval; LT: Liver transplantation; OR: Odds ratio; UCI: Upper confidence interval

Table S16: List of Studies Excluded from Meta-analysis

S.No.	Study name	Reason of exclusion from meta-analysis
1	John 2021	Study removed from meta-analysis due to weight issue
2	Kumagi 2010a	Qualitative only (p-value)
3	Reiberger 2019	Qualitative only (p-value)
4	Murillo 2020; Perez 2019	Outcome NOI*
5	Carbone 2016	Qualitative only
6	Bonnand 1999	Outcome NOI*
7	Momah 2012; Momah 2011	Only % reported, N not given
8	Hofer 2024	Outcome NOI*
9	Huet 2008	Outcome NOI*
10	Copaci 2011	Outcome NOI*
11	Leeman 2014	Outcome NOI*
12	Azemoto 2009	Outcome NOI*
13	Lammers 2018	Outcome NOI*
14	Liu 2016	Outcome NOI*
15	Tanaka 2015	Outcome NOI*
16	Takano 2020	Outcome NOI*
17	Lam 2023	Outcome NOI*
18	Haddadin 2019	Qualitative
19	Teh 2014	Qualitative only (p-value)
20	Reig 2019	Qualitative only (p-value)
21	Perez 2018; Perez 2017	Outcome NOI*
22	Zhang 2015; Li 2016	Qualitative only (p-value)

S.No.	Study name	Reason of exclusion from meta-analysis
23	Corpechot 2016	Qualitative only (p-value)
24	Fujinaga 2017	Outcome NOI*
25	Gerussi 2020a	Outcome NOI*
26	Roberts 2024	Outcome NOI* (relative rates reported)
27	Wong 2025	Outcome NOI*
28	Wang 2024	Outcome NOI*
29	Kugiyama 2025	Outcome NOI*
30	Ding 2025a	Outcome NOI*
31	Iwadare 2024	Outcome NOI*
32	Hayashi 2024	Outcome NOI*
33	Qu 2019	Outcome NOI*
34	Zhang 2025	Outcome NOI*
35	Tadros 2024	Outcome NOI*
36	Ritter 2025	Outcome NOI*
37	Okumura 2025	Outcome NOI*
38	Rachel 2026	Outcome NOI*
39	Arvaniti 2025	Outcome NOI*
40	Padilla-Lopez 2026	Outcome NOI*

*Meta-analysis was not feasible for these studies because appropriate surrogate endpoints were unavailable and the response criteria were defined inconsistently across studies, precluding meaningful pooling of results.

NOI: Not of interest
